# Site-Specific Histidine Aza-Michael Addition in Proteins Enabled by a Ferritin-Based Metalloenzyme

**DOI:** 10.1021/jacs.4c14446

**Published:** 2024-11-05

**Authors:** Jo-Chu Tsou, Chun-Ju Tsou, Chun-Hsiung Wang, An-Li A. Ko, Yi-Hui Wang, Huan-Hsuan Liang, Jia-Cheng Sun, Kai-Fa Huang, Tzu-Ping Ko, Shu-Yu Lin, Yane-Shih Wang

**Affiliations:** † Institute of Biological Chemistry, Academia Sinica, Taipei 11529, Taiwan; ‡ Institute of Biochemical Sciences, National Taiwan University, Taipei 10617, Taiwan

## Abstract

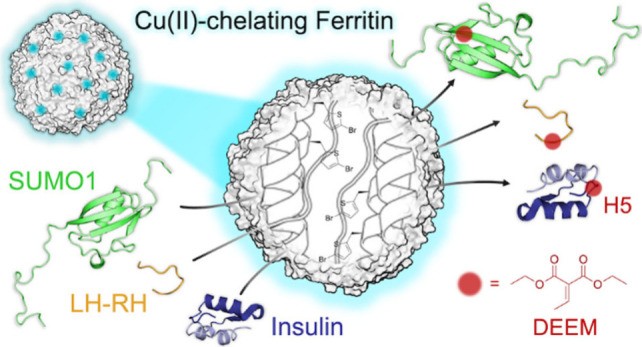

Histidine modifications of proteins are broadly based on chemical methods triggering N-substitution reactions such as aza-Michael addition at histidine’s moderately nucleophilic imidazole side chain. While recent studies have demonstrated chemoselective, histidine-specific modifications by further exploiting imidazole’s electrophilic reactivity to overcome interference from the more nucleophilic lysine and cysteine, achieving site-specific histidine modifications remains a major challenge due to the absence of spatial control over chemical processes. Herein, through X-ray crystallography and cryo-electron microscopy structural studies, we describe the rational design of a nature-inspired, noncanonical amino-acid-incorporated, human ferritin-based metalloenzyme that is capable of introducing site-specific post-translational modifications (PTMs) to histidine in peptides and proteins. Specifically, chemoenzymatic aza-Michael additions on single histidine residues were carried out on eight protein substrates ranging from 10 to 607 amino acids including the insulin peptide hormone. By introducing an insulin-targeting peptide into our metalloenzyme, we further directed modifications to be carried out site-specifically on insulin’s B-chain histidine 5. The success of this biocatalysis platform outlines a novel approach in introducing residue- and, moreover, site-specific post-translational modifications to peptides and proteins, which may further enable reactions to be carried out *in vivo*.

Post-translational protein modifications (PTMs) are highly regulated and efficient mechanisms involved in coordinating most if not all known cellular processes.^1^ Being able to biochemically reproduce PTMs both chemo- and site-selectively,^2^ as nature would, would serve a broad range of applications, specifically in probing proteins and producing homogeneous bioconjugates^3^ such as antibody– and protein–drug conjugates for therapeutic use.^4^ To achieve this in proteins, biocatalysis platforms supported by the development of novel, efficient enzymes are much in need.^5^ While it is common to target the highly nucleophilic lysine and cysteine side chains to elicit chemoselectivity, their higher reactivity entails lower selectivity for residues to be targeted in a site-selective manner.^2^ Moreover, modifying residues that often host a vast array of native PTMs is likely to compromise protein activity.^1^

Targeting of the less yet still nucleophilic and also less frequently modified histidine imidazole side chain^6,7^ (that may still be, for instance, phosphorylated for mediating protein–protein interactions^8^ or methylated for histone epigenetic regulation^9^) (Scheme 1) can therefore be of great interest for the purposes of generating biologically active, homogeneous products, with modifications more likely to be bioorthogonal to native PTMs. Histidine’s similar abundance (∼2%) to cysteine in proteins^10^ also poses it as a desirable target for modification when presented on protein surfaces for temporary functional regulation, considering its various indispensable roles in catalyzing nucleophilic reactions,^11^ coordinating metals,^6^ hydrogen bonding, and transferring protons within enzymes.^12^ In this study, we thus aim to design a site-selective histidine modification biocatalysis platform inspired by natural PTM mechanisms, using bioorthogonal adaptors to guide site-specificity.

**Scheme 1:**
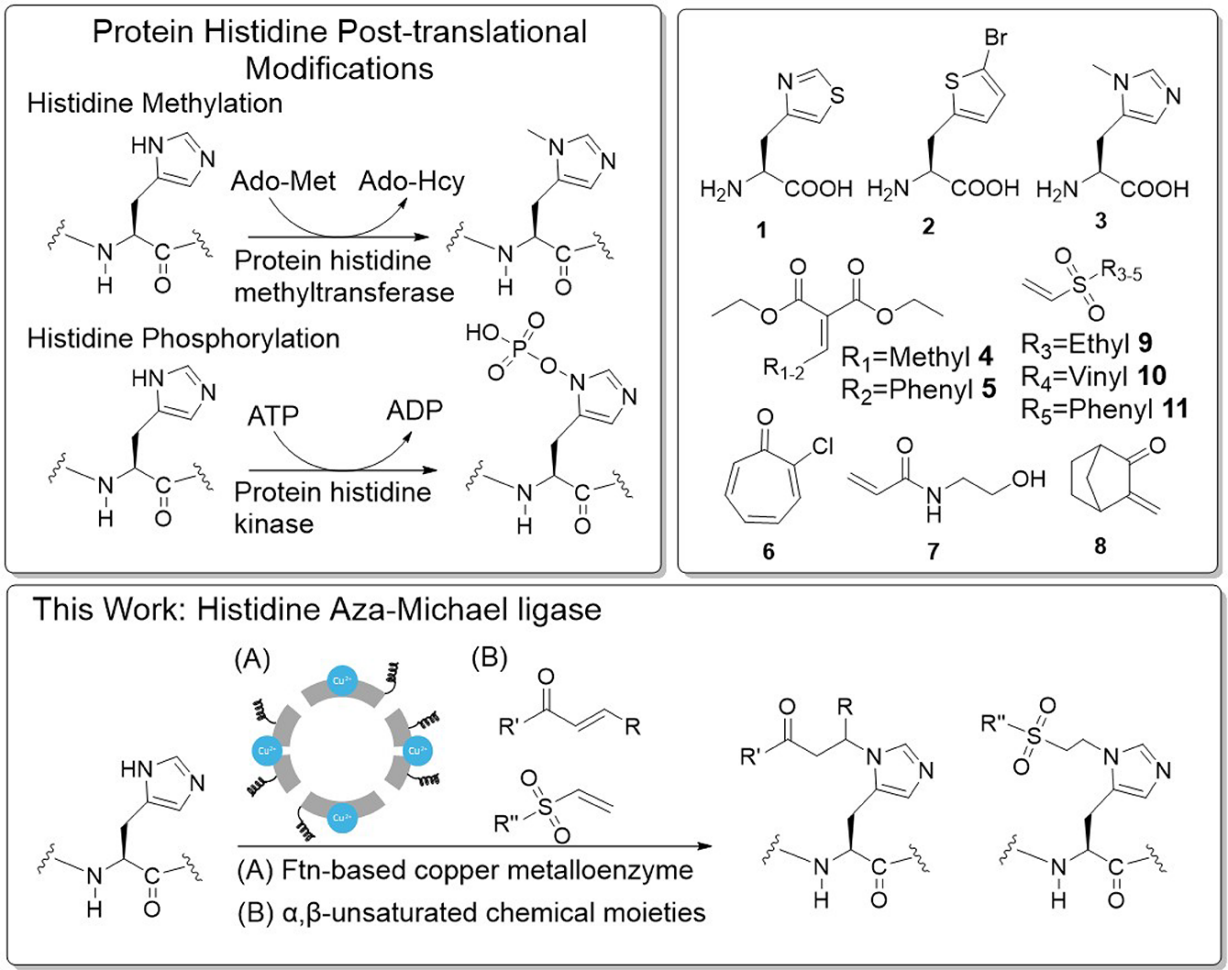
Protein Histidine PTMs, ncAAs and α,β-Unsaturated Chemicals, and Rational Design of a Ferritin-Based aza-Michael Ligase for Site-Specific Protein Histidine Modifications

Although increasing electrophile concentrations^2,13^ and tunable metallaphotoredox potentials^14^ can help obtain higher yield, it is often at the cost of selectivity. The presence of the many interfering, more reactive, and repetitive functional groups within proteins further impedes selective formation of covalent bonds with histidine’s less nucleophilic side chain. Hence, histidine-specific modifications are often dependent on ligand- and/or sequence-directed chemistry in addition to harsh reaction conditions to achieve chemoselectivity.^15^ While novel reagents have also been explored for these to be performed under relatively mild reaction conditions^16^ and chemical derivatization followed by further HPLC purification may help achieve protein single-site histidine modification,^17^ methodologies to achieve site-selectivity remain underexplored. With the ultimate objective to create a biocatalysis platform that could be expressed *in vivo*, we report the rational design of an artificial human ferritin heavy chain (FTH1)-based metalloprotein to achieve histidine-specific aza-Michael addition with α,β-unsaturated chemical moieties (Scheme 1).

Artificial metalloproteins are powerful biocatalysts for their metal-chelating properties. In fact, many of those reported are based on scaffold proteins engineered to anchor transition metals.^18^ In search of a protein scaffold for chelating Cu(II), the Lewis acid that is commonly applied to catalyze aza-Michael addition, to modify proteins, we came across a L56H/R63H/E67H ferritin (Ftn) mutant (Figure S1B) that assembles only upon copper binding between the above three histidine residues alongside a native H60^19^ (Figures 1A and S1A). As an iron storage and release self-assembling protein cage, wild-type Ftn (wt-Ftn) comprises 24 FTH1 subunits with 12 C_2_ axes^20^ (Figure S2). Utilizing the large area the C_2_ interfaces cover altogether, mutated FTH1 subunits were designed to assemble only upon divalent copper binding at the C_2_ interfaces hosting their histidine mutations. To still allow these sites and/or structurally induced nearby residues to chelate Cu(II) for catalyzing histidine modifications while allowing the ferritin cage to reserve its self-assembling property, instead of modifying all of the above-mentioned four copper-chelating histidines, we attempted to replace only one (R63) or two (R63/E67), which are noncopper-chelating, with histidine analogs **1**–**3** (Scheme 1) (4-thiazolyl-l-alanine, **1**; 2-(5-bromothienyl)-l-alanine (BtA), **2**; 3-methyl-l-histidine (MeH), **3**), separately, via genetic code expansion^21^ (Figure S3). These noncanonical amino acids (ncAAs) were previously reported to chelate Cu(II) in either nature or synthesized ligands. Using the *Mm*PylRS-N346A/C348A·tRNA^Pyl^ pair^22^ bioorthogonal to the *Escherichia coli* system, we generated six Ftn variants (Figure 1B).

**Figure 1:**
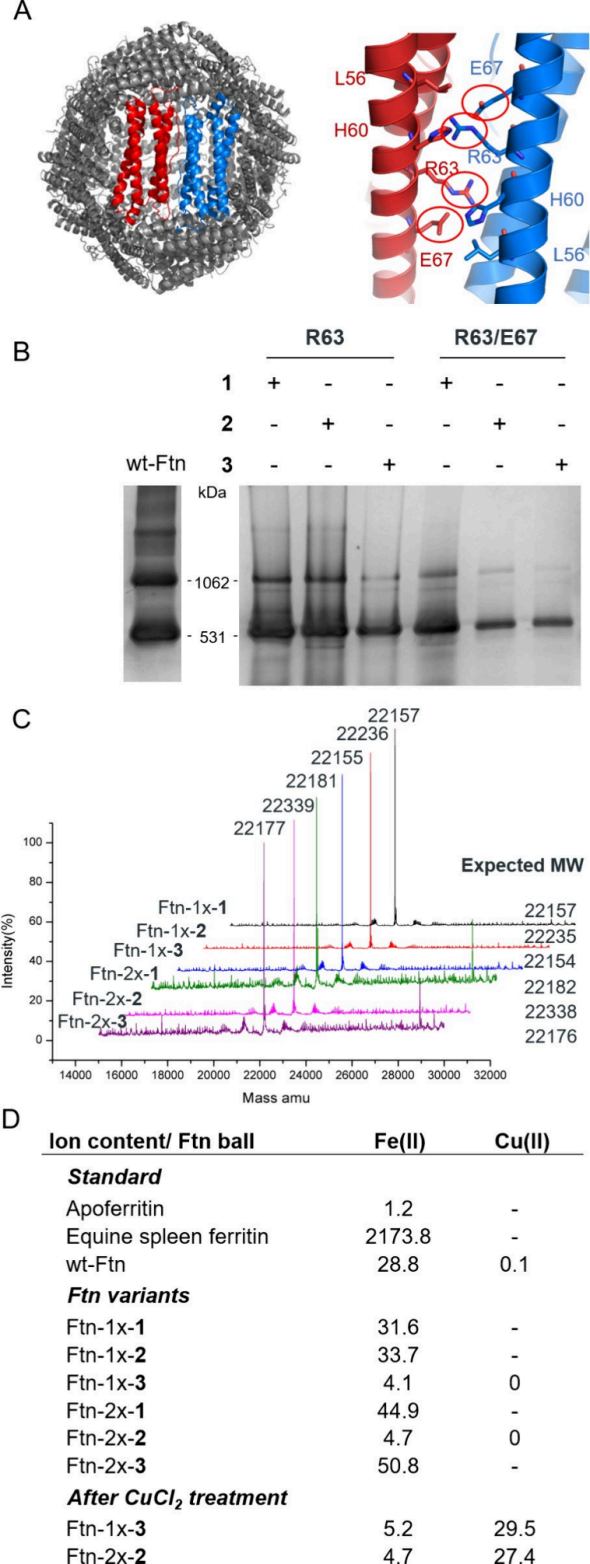
(A) R63 and E67 of wt-Ftn (PDB code: 2FHA) are targeted for histidine analogs **1**–**3** (Scheme 1). Incorporation at the C_2_ interface, as shown between two FTH1 monomers (red and blue). (B) 4–12% native PAGE analyses of the six ncAA-incorporated Ftn variants, with (C) ESI-MS analyses of the six ncAA-incorporated Ftn variants and (D) their Fe(II) and Cu(II) contents measured by ICP-MS alongside those of apoferritin, equine spleen ferritin, and wt-Ftn.

A native PAGE analysis of the Ftn variants alongside wt-Ftn was first carried out to verify their assembly (Figure 1B). Individual molecular weights (MWs) were then confirmed by ESI-MS (Figure 1C). ICP-MS analyses were further performed to measure individual Fe(II) contents (Figure 1D), alongside commercial standards of apoferritin and equine spleen ferritin. The large disparity between Fe(II) contents in the commercial standards can be explained by Fe(II) binding only to apoferritin’s surface and not stored inside its cavity. Fe(II) content between Ftn variants and in comparison with wt-Ftn also varied significantly with signature feature amounts of 0, 24, and 48 Fe(II) ions along with the FTH1 24mers stoichiometry (Figure 1D). In the crystal structure of Ftn-1x-**3** (Table S1, Figures S1C–D) without Fe(II) or Cu(II) content, additive bending hexaethylene glycol in the C2 interface binds to MeH63 (Figure S1D) and generates another potential metal binding pocket, along with the ^59^SHEE^63^ MeHEHA^67^E sequence that can be found in the Ftn-L56H/R63H/E67H mutant (Figures S1A, S1C).

The cryo-EM structural models (Table S2, Figures S4–S7) of Ftn-2X-**2** (Figures 2A,B) and Ftn-2x-**3** (Figures 2E,F) and these two Ftns with CuCl_2_ supplement (Figures 2C,D, 2G,H) were solved and revealed two detailed metal binding environments. Instate of binding metal, either Fe(II) or Cu(II), at the C2 interface of Ftn-2x-**2**, 63BtA, 67BtA, and H60 form an aromatic cluster of pi–pi interactions (Figures 2A,C). Thus, E27/E62/H65 turns to bind to Cu(II) in Ftn-2x-**2**-Cu(II) (Figure 2D) by replacing the sodium ion (Figure 2B) originally. In fully Fe(II)-bonded Ftn-2x-**3**, S59/H60/63MeH at the C2 interface (Figure 2E) and E27/E62/H65 (Figure 2F) binding sites are identified. In a comparison to the ICP-MS data and structural models of Ftn-1x-**3** (Figure S1D) and Ftn-2x-**3** (Figure 2E), the E67MeH mutation displays dual roles in aromatic ring stacking and metal chelating in controlling Fe(II) and Cu(II) binding. After Cu(II) supplementation, S59/H60/63MeH at the C2 interface loses the binding to Fe(II) and forms stable hydrophobic interactions (Figure 2G). The local interactions make the binding distance of E27 slightly longer, 3.1 Å, and lead to another Cu(II) binding site at E62 and E107. With the original E27/E62/H65 binding motif, Ftn-2x-**3**-Cu(II) forms a nonheme dicopper center (Figure 2H).

**Figure 2:**
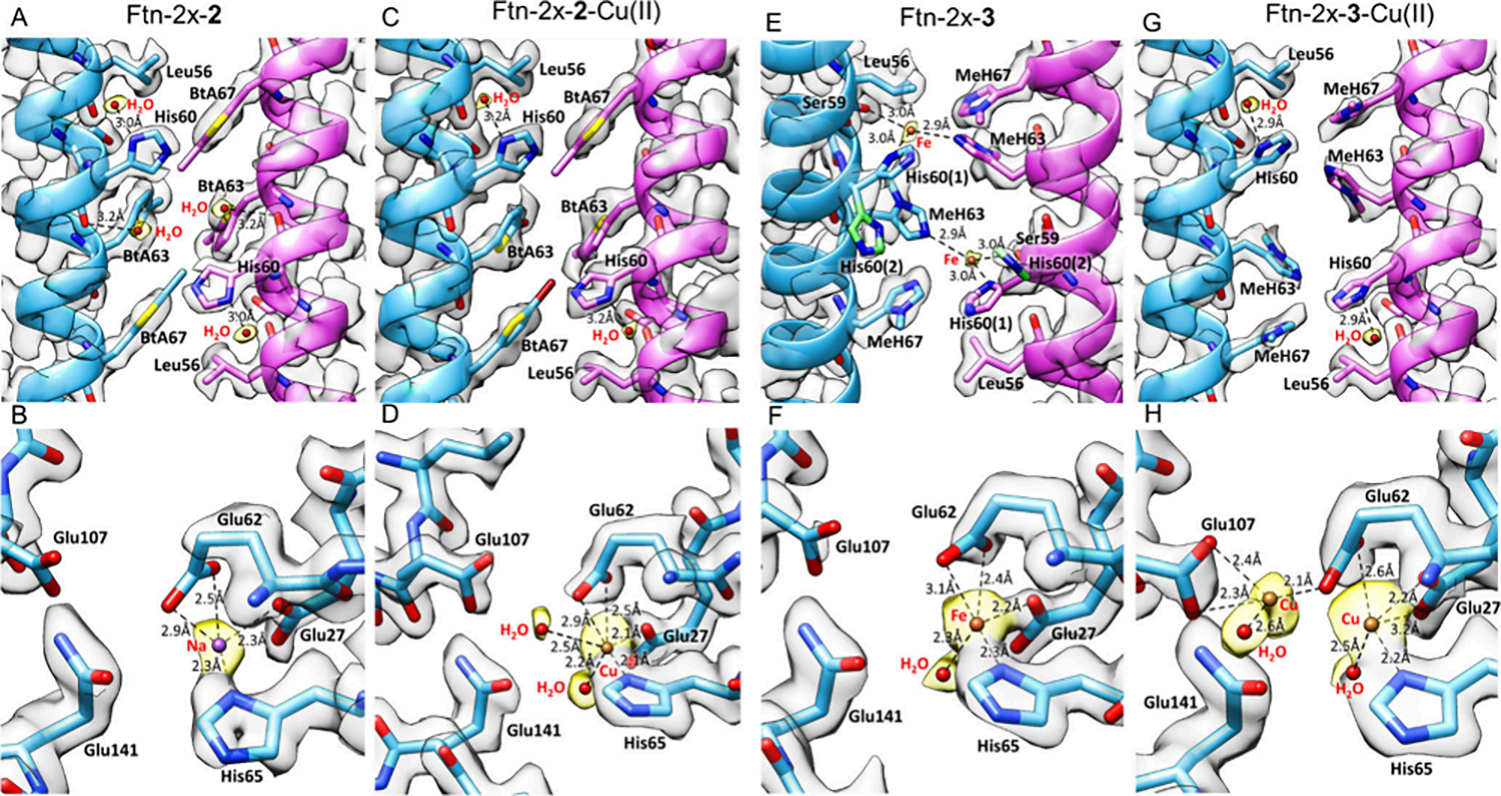
Cryo-EM structures of metal binding sites at the C2 engineered interface and monomer of (A, B) Ftn-2x-**2** (PDB code 9JQB), (C, D) Ftn-2x-**2-**Cu(II) (PDB code: 9JQC), (E, F) Ftn-2x-**3** (PDB code: 9JQD), and (G, H) Ftn-2x-**3**-Cu(II) (PDB code: 9JQE). Protein residues are depicted as light blue sticks, sodium ions in purple, copper in yellow, and iron in orange. Cryo-EM density maps are colored transparent gray.

In order to minimize interference of Fe(II) during catalysis, we selected the two variants with the lowest iron contents, Ftn-1x-**3** and Ftn-2x-**2** (Figure 1D), for further treatment and characterization in the catalytic property. Upon CuCl_2_ treatment, Cu(II) contents in the two selected variants notably increased compared with wt-Ftn, whereas there were no significant changes to their Fe(II) contents. With the structural analysis of the Cu(II) binding site at E27/E62/H65 of Ftn-2x-**2**-Cu(II) and Ftn-2x-**3**-Cu(II), these results indicated that our engineering is responsible for the impairment of Ftn’s Fe(II) binding. To examine the new metalloenzymes’ viability as aza-Michael ligases, we attempted to modify the human insulin protein with diethyl ethylidenemalonate (DEEM, **4**, Scheme 1).

Out of insulin’s 51 amino acids, two are histidine (B-chain H5 and H10)^23^ (Figure 3A). Being an essential medication for treating diabetes, much effort has been made to chemically modify this large peptide hormone to fine-tune its time–action profile. Though existing technologies have indeed increased its varieties considerably, most modifications target the most reactive lysine residue. Hence, establishing a biocatalysis platform capable of site-selectively modifying histidine is without doubt invaluable for further enriching the diversity of insulin analogs. Single modifications of DEEM (1x-DEEM), as represented by 186 Da shifts on MALDI-TOF-MS spectra, suggested our newly designed metalloenzymes to be carrying out modifications with specificity (Figures 3A–C and S8–S10). Conversion rates (CVRs) carried out by Ftn-1x-**3** and Ftn-2x-**2** with 2 equiv of DEEM were 20% and 33%, respectively, making the latter more desirable for further characterization. For Ftn-2x-**2**, seven other α,β-unsaturated reagents (**5**–**11**) (Scheme 1) were tested to modify insulin, yielding CVRs between 0 and 44% (Figure 4A and Figures S11–S12). Quite interestingly, with α,β-unsaturated sulfones (**10**, **11**), the enzyme exhibited comparably higher activity yet less selectivity in modifying histidine.

**Figure 3:**
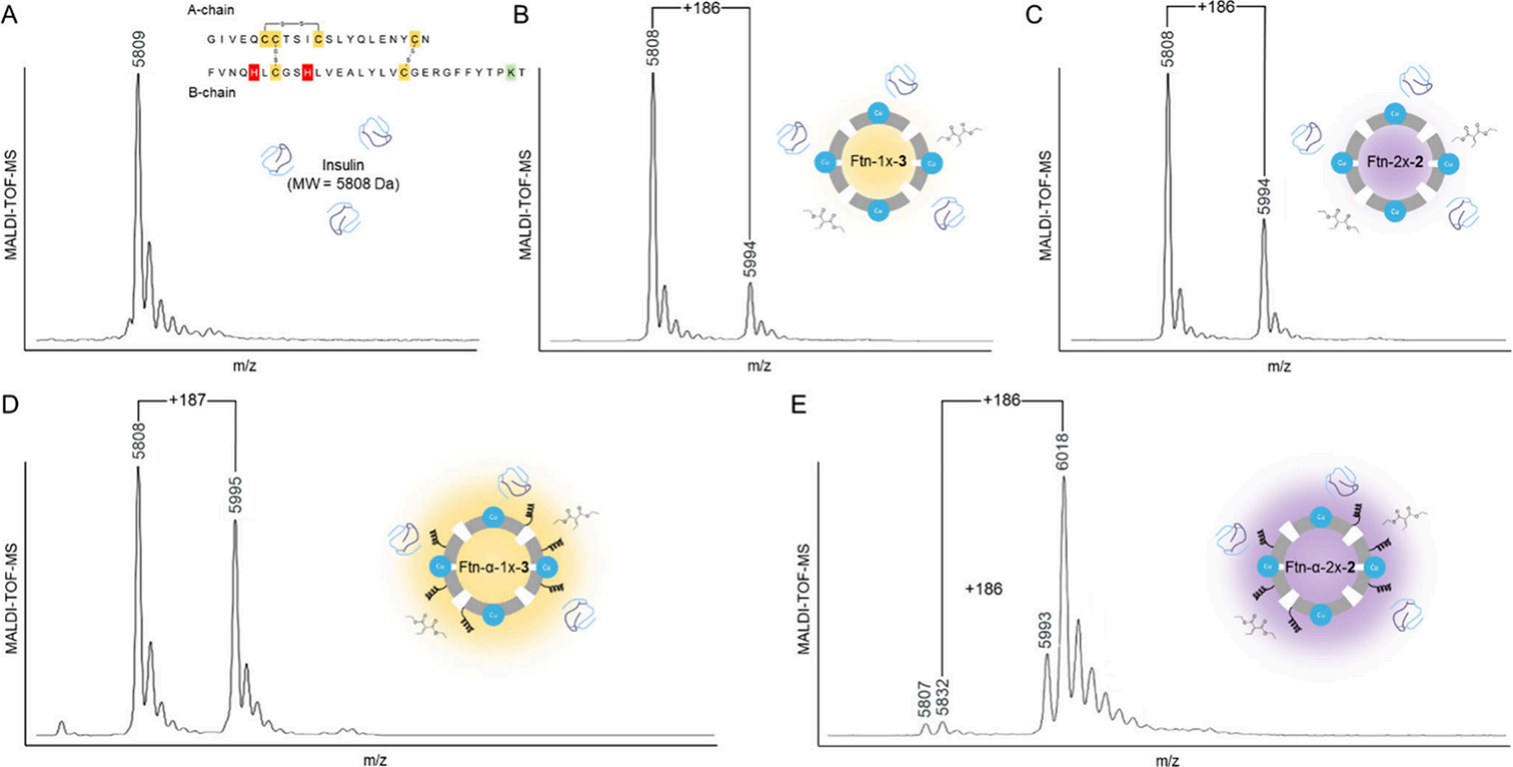
MALDI-TOF-MS analyses of the insulin protein either (A) unmodified or modified with a single DEEM adduct (+186 *m*/*z* or Da) by CuCl_2_-treated (B) Ftn-1x-**3** (CVR = 20%, turnover number (TON) = 4.0 h^–1^ ball^–1^), (C) Ftn-2x-**2** (CVR = 33%, TON = 6.6 h^–1^ ball^–1^), (D) Ftn-α-1x-**3** (CVR = 46%, TON = 9.2 h^–1^ ball^–1^), and (E) Ftn-α-2x-**2** (CVR = ∼100%, TON = 24 h^–1^ ball^–1^).

**Figure 4:**
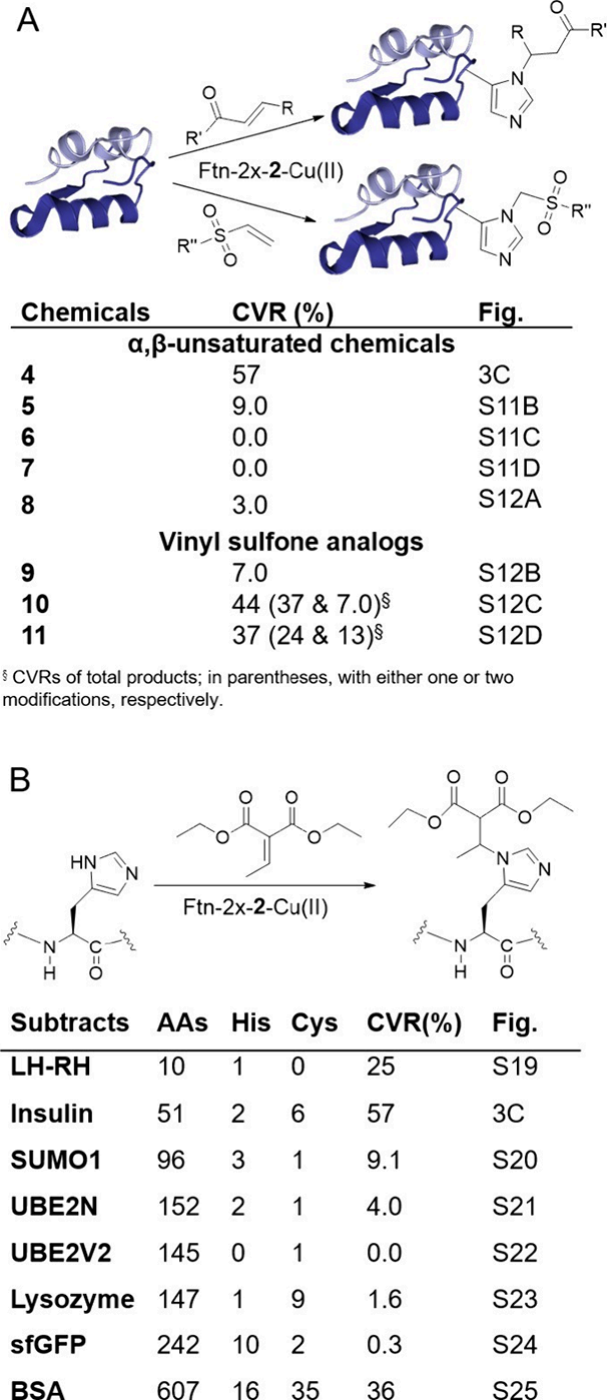
(A) Yield of Ftn-2x-**2**’s α,β-unsaturated chemical moiety-modified insulin products; (B) Amino acid, histidine and cysteine count, and yield of Ftn-2x-**2**’s DEEM-modified peptide and protein products.

To investigate Ftn-2x-**2**’s substrate scope as an aza-Michael ligase, we examined its catalytic activity alongside DEEM treatment on seven additional protein substrates that ranged in length from 10 to 607 residues: a luteinizing hormone-releasing hormone analog ([d-Ala^6^, *N*-Me-Leu^7^]-LH-RH), the small ubiquitin-related modifier 1 (SUMO1), lysozyme, human ubiquitin conjugating enzymes UBE2 V2 and UBE2N, superfolder green fluorescent protein (sfGFP), and bovine serum albumin (BSA). Aside from UBE2 V2 hosting no histidine, again, only adducts with single modifications were observed (Figures 3C, 4B, and S19–S25). MALDI-TOF-MS/MS further confirmed site-specific DEEM modification on [d-Ala^6^, *N*-Me-Leu^7^]-LH-RH’s H2, whereas due to the lower yield of SUMO1–1x-DEEM, however, no DEEM modifications were revealed in its N-terminal and three histidine-containing peptides during ESI-MS/MS analyses (Figures S26–S30). Nonetheless, a carbamidomethyl modification commonly observed in in-gel digestion samples has been identified on SUMO1’s C51, indicating that its only cysteine is not DEEM-modified (Figure S31).

With these promising preliminary data, we continued to characterize DEEM’s location in Ftn-2x-**2**’s modified insulin products by reducing all disulfide bonds with 30 equiv of DTT (Figure 5A). In both modified and unmodified insulin, A-chains were found to remain soluble, whereas B-chains precipitated. MALDI-TOF-MS analyses further revealed two DEEM-modified species (insulin B chain-1x-DEEM and −2x-DEEM) to be present, with the second only in trace amounts, but are both located only on the insulin B-chain, where the two histidine residues (Figures 5B,C and S32).

**Figure 5:**
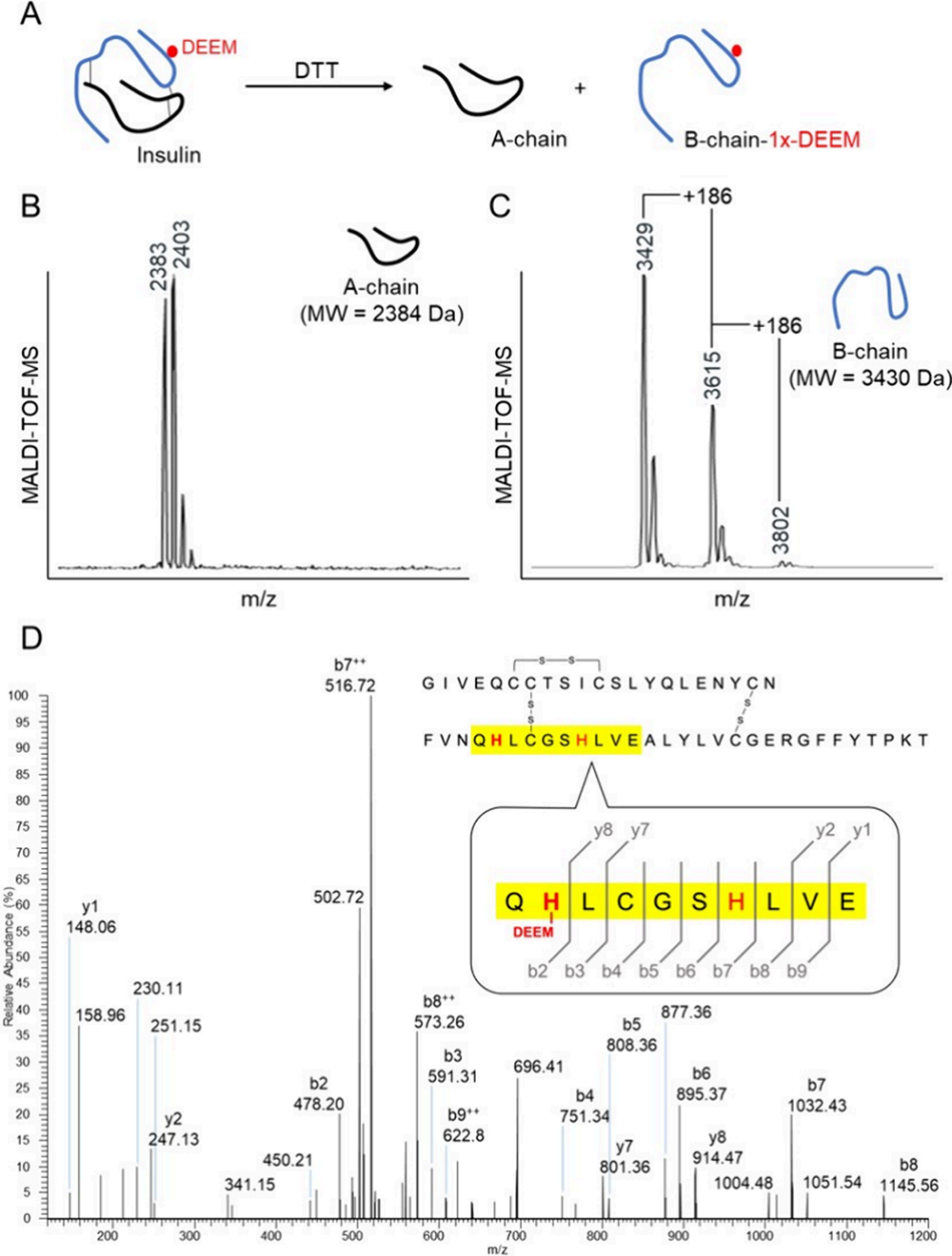
(A) Ftn-2x-**2**’s DEEM-modified insulin product is treated with DTT to be digested into (B) A- and (C) B-chains, which are then separately analyzed by MALDI-TOF-MS to determine DEEM adduct’s localization. (D) MALDI-TOF-MS/MS analysis of Ftn-α-2x-**2**’s DEEM-modified insulin product revealed the site-selective modification to be carried out on only insulin’s B-chain H5.

In cells, chemo- and site-selective PTM is guided by the enzyme recognition of target sequences in protein substrates. Based on this rationale, to achieve site-selectivity and increase the catalytic efficiency of our metalloenzymes, we intend to engineer a target recognition peptide (TRP) into our Ftn variants. αCT is a 16-amino acid peptide isolated from the insulin receptor’s substrate-binding domain, and is known to build contacts with both insulin chains^24^ (Figure S33). We noted that H5 of insulin B-chain is located within its N-terminal nonstructural region, whereas H10 from the same chain is located within its first α-helical region, which would be stabilized upon insulin’s interaction with αCT. Hence, we hypothesize that by utilizing αCT as a TRP, we could site-selectively target H5 for modification, taking advantage of its flexibility to further interact with Cu(II). To optimize the design of our meta-lloenzymes, we generated a series of His-tagged Ftn-αCT-fusion variants (Figures 3D,E), with the peptides fused in distinct orders (Figure S34A) and via flexible linkers of varying lengths (Figure S35A). The N-termini of wt-FTH1 subunits are known to be surface-exposed, while the C-termini are buried within the protein (Figure S2). Interestingly, native and SDS-PAGE results revealed that the fusion of αCT at FTH1’s N-terminus (α-Ftn) would impair Ftn’s assembly, making the protein insoluble in buffer, whereas the C-terminally fused variant (Ftn-α) remained soluble, assembled, and could be readily purified through Ni^2+^-NTA columns via its C-terminal 6xHis-tag (Figures S34B–S34D).

To then adjust the linker’s length so to understand its correlation with the 6xHis-tag’s accessibility, three constructs with linkers of diverging lengths (S(GGGGS)_1–3_, L1–3) were produced (Figure S35A). A native PAGE analysis confirmed all variants’ assembly (Figure S34E). Through SDS-PAGE visualization of elution profiles, we observed that among the three variants, the longer the linker, the higher the binding affinity (Figures S35B–S35D). Thus, further engineering of Ftn-α-1x-**3** and Ftn-α-2x-**2** variants were completed with the L3 linker. Homogenous ncAA incorporations in both variants were first corroborated via ESI-MS (Figure S36). MALDI-TOF-MS analyses then confirmed both variants to yield only single DEEM modification peaks and near 26% and 57% CVR increases, respectively, as compared to their respective counterparts without αCT-fusions (Figures 3B,E and S37–S38). These increases indicated our design of a TRP to be successful in increasing the ligases’ catalytic efficiency. Moreover, one equivalent of insulin and two equiv of DEEM, the Michael acceptor, were used in enzymatic reactions ([insulin]:[DEEM]:[Ftn-a-2x-**2**] = 1:2:0.04) to achieve near quantitative conversion by Ftn-α-2x-**2**, although it is with a lower TON. Ftn-α-2x-**2**’s modified products were further digested by endoproteinase Glu-C and analyzed by MALDI-TOF-MS/MS, demonstrating DEEM modifications to be carried out site-selectively on only H5 of insulin’s B-chain, as we intended for (Figure 5D). DLS and FEG-TEM analyses were additionally carried out to confirm Ftn-α-2x-**2**’s similar morphology with wt-Ftn in terms of size and shape when mixed with insulin (Figures S39–S40). Combining these results, we report Ftn-α-2x-**2** to be an effective aza-Michael ligase that site-specifically targets H5 of the insulin B-chain. Further research removing Fe(II) from Ftn variants prior to CuCl_2_ treatment will be reported in due course to elucidate factors that contribute to the catalysis with new Cu(II) binding site.

In conclusion, we have reported a biocatalysis platform inspired by native PTM mechanisms that can carry out site-selective histidine modifications on proteins. Complementing existing methods for chemoselective histidine modification, we envision this platform to empower us to produce a wider array of homogeneous bioconjugates. This human protein-based platform further sets the stage for developing novel enzyme therapies, for instance, to modify native insulin of diabetic patients by fine-tuning its action profile *in vivo*.

## Supporting Information Available

List of abbreviations, Ftn variants, peptide/protein sequences, plasmids and primers, Materials and Method, Supplementary Tables S1–S4, and Supplementary Figures S1–S35, including results from MS, X-ray crystallography, Cryo-EM, PAGE, DLS and TEM analyses (PDF)
