# Supplementary material for: Site-Specific Histidine Aza-Michael Addition in Proteins Enabled by a Ferritin-Based Metalloenzyme
Source: J Am Chem Soc. 2024 Nov 5;146(49):33309–15. doi: 10.1021/jacs.4c14446 (PMC11638945; doi:10.1021/jacs.4c14446)
Supplement: Supplementary file 1 — ja4c14446_si_001.pdf [file ja4c14446_si_001.pdf]

Site-specific histidine aza-Michael addition in proteins enabled by a ferritin-based metalloenzyme

SUPPORTING INFORMATION

Jo-Chu Tsou[1,†], Chun-Ju Tsou[1,2,†], Chun-Hsiung Wang[1, †], An-Li A. Ko[1,†], Yi-Hui Wang[1], Huan-Hsuan Liang[1,2], Jia-Cheng Sun[1], Kai-Fa Huang[1], Tzu-Ping Ko[1], Shu-Yu Lin[1] and Yane-Shih Wang[1,2,*]

[1] Institute of Biological Chemistry, Academia Sinica, Taipei 11529, Taiwan

[2] Institute of Biochemical Sciences, National Taiwan University, Taipei 10617, Taiwan

[†] These authors have contributed equally to this work and share first authorship.

[*] Corresponding Author: yaneshihwang@gate.sinica.edu.tw, ericyswang@gmail.com

TABLE OF CONTENTS

LIST OF ABBREVIATIONS……………………………………………………………………3

LIST OF FERRITIN VARIANTS……………………………………………………………5

PEPTIDE AND PROTEIN SEQUENCES……………………………………………………6

LIST OF PLASMIDS AND PRIMERS………………………………………………………8

MATERIALS AND METHOD………………………………………………………………10

SUPPLEMENTARY TABLES………………………………………………………………18

SUPPLEMENTARY FIGURES………………………………………………………………21

LIST OF ABBREVIATIONS

**1**, ThzA	4-thiazolyl-L-alanine
**2**, BrThA	2-(5-bromothienyl)-L-alanine
**3**, MeH	3-methyl-histidine
**4**, DEEM	Diethyl ethylidenemalonate
**5**, DEBM	Diethyl benzylidenemalonate
Amp	Ampicillin
CVR	Conversion rate
DHB	2,5-dihydroxybenzoic acid
DLS	Dynamic light scattering
DTT	Dithiothreitol
E. coli	*Escherichia coli*
ESI-MS	Electrospray ionization mass spectrometry
FEG-TEM	Field emission gun transmission electron microscope
FTH1	Human ferritin heavy chain
Ftn	Ferritin
ICP-MS	Inductively coupled plasma mass spectrometry
IPTG	Isopropyl β-D-thiogalactoside
L1	SGGGGS linker
L2	$S(GGGGS)_2$ linker
L3	$S(GGGGS)_3$ linker
LB	Lysogeny broth
LH-RH	Luteinizing hormone-releasing hormone
MALDI-TOF-MS	Matrix assisted laser desorption ionization-time of flight mass spectrometry
MALDI-TOF-MS/MS	Matrix assisted laser desorption ionization-time of flight tandem mass spectrometry
*Mm*PylRS	*Methanosarcina mazei* pyrrolysyl-tRNA synthetase
MW	Molecular weight
ncAA	Non-canonical amino acid
PCR	Polymerase chain reaction
PAGE	Polyacrylamide gel electrophoresis
PTM	Post-translational protein modification
SDS	Sodium dodecyl sulfate
SEC	Size exclusion chromatography

sfGFP	Superfolder green fluorescent protein
Sp	Streptomycin
SUMO1	Small ubiquitin-related modifier 1
TFA	Trifluoroacetic acid
TON	Turnover number
Tris	Trisaminomethane
TRP	Target recognition peptide
$tRNA^{Pyl}$	Pyrrolysine transfer RNA
Wt-Ftn	Wild-type ferritin

LIST OF FERRITIN VARIANTS

Non-canonical amino acid (ncAA)-incorporated Ferritin (Ftn) variants

Ftn-1x-**1**	FTH1-R63ThzA
Ftn-1x-**2**	FTH1-R63BrThA
Ftn-1x-**3**	FTH1-R63MeH
Ftn-2x-**1**	FTH1-R63ThzA/E67ThzA
Ftn-2x-**2**	FTH1-R63BrThA/E67BrThA
Ftn-2x-**3**	FTH1-R63MeH/E67MeH

Ftn variants with αCT-fusion

α-Ftn	αCT-L3- FTH1-6xHis-tag
Ftn-α	FTH1-L3-αCT-6xHis-tag
Ftn-L1-α	FTH1-L1-αCT-6xHis-tag
Ftn-L2-α	FTH1-L2-αCT-6xHis-tag
Ftn-α-1x-**1**	FTH1-R63ThzA-L3-αCT-6xHis-tag
Ftn-α-1x-**2**	FTH1-R63BrThA-L3-αCT-6xHis-tag
Ftn-α-1x-**3**	FTH1-R63MeH-L3-αCT-6xHis-tag
Ftn-α-2x-**1**	FTH1-R63ThzA/E67ThzA-L3-αCT-6xHis-tag
Ftn-α-2x-**2**	FTH1-R63BrThA/E67BrThA-L3-αCT-6xHis-tag
Ftn-α-2x-**3**	FTH1-R63MeH/E67MeH-L3-αCT-6xHis-tag

PEPTIDE AND PROTEIN SEQUENCES

**Name**	**Sequence**
αCT	TFEDYLHNVVFVPRPS
Human ferritin heavy chain (FTH1)	MTTASTSQVRQNYHQDSEAAINRQINLELYASYVYLSMS<br>YYFDRDDVALKNFAKYFLHQSHEEREHAEKLMKLQNQR<br>GGRIFLQDIKKPDCDDWESGLNAMECALHLEKNVNQSL<br>LELHKLATDKNDPHLCDFIETHYLNEQVKAIKELGDHVT<br>NLRKMGAPESGLAEYLFDKHTLGDSDNES
Luteinizing hormone-releasing hormone (LH-RH) analog ([D-Ala^6, *N*-Me-Leu^7]-LH-RH)	Glu-His-Trp-Ser-Tyr-D-Ala-(*N*-Me)Leu-Arg-Pro-Gly-NH_2
Small ubiquitin-related modifier 1 (SUMO1)	SDQEAKPSTEDLGDKKEGEYIKLKVIGQDSSEIH(34)FKV<br>KMTTH(42)LKKLKESYCQRQGVPMNSLRFLFEGQRIADN<br>H(74)TPKELGMEEEDVIEVYQEQTGGG
Recombinant human ubiquitin conjugating enzyme UBE2N	MAGLPRRIIKETQRLLAEPVPGIKAEPDESNARYFHVVIA<br>GPQDSPFEGGTFKLELFLPEEYPMAAPKVRFMTKIYHPN<br>VDKLGRICLDILKDKWSPALQIRTVLLSIQALLSAPNPDD<br>PLANDVAEQWKTNEAQAIETARAWTRLYAMNNI
Recombinant human ubiquitin conjugating enzyme UBE2V2	MAVSTGVKVPRNFRLLEELEEGQKGVGDGTVSWGLEDD<br>EDMTLTRWTGMIIGPPRTNYENRIYSLKVECGPKYPEAPP<br>SVRFVTKINMNGINNSSGMVDARSIPVLAKWQNSYSIKV<br>VLQELRRLMMSKENMKLPQPPEGQTYNN
Lysozyme	MRSLLILVLCFLPLAALGKVFGRCELAAAMKRHGLDNY<br>RGYSLGNWVCAAKFESNFNTQATNRNTDGSTDYGILQIN<br>SRWWCNDGRTPGSRNLCNIPCSALLSSDITASVNCAKKIV<br>SDGNGMNAWVAWRNRCKGTDVQAWIRGCRL
Superfolder green fluorescent protein (sfGFP)	MSKGEELFTGVVPILVELDGDVNGHKFSVRGEGEGDAT<br>NGKLTLKFICTTGKLPVPWPTLVTTLTYGVQCFSRYPDH<br>MKRHDFFKSAMPEGYVQERTISFKDDGTYKTRAEVKFE<br>GDTLVNRIELKGIDFKEDGNILGHKLEYNFNSHNVYITAD<br>KQKNGIKANFKIRHNVEDGSVQLADHYQQNTPIGDGPV<br>LLPDNHYLSTQSVLSKDPNEKRDHMVLLEFVTAAGITHG<br>MDELYKGSEL
Bovine serum albumin (BSA)	MKWVTFISLLLLFSSAYSRGVFRRDTHKSEIAHRFKDLGE<br>EHFKGLVLIAFSQYLQQCPFDEHVKLVNELTEFAKTCVAD<br>ESHAGCEKSLHTLFGDELCKVASLRETYGDMADCCEKQ<br>EPERNECFLSHKDDSPDLPKLKPDPNTLCDEFKADEKKF<br>WGKYLYEIARRHPYFYAPELLYYANKYNGVFQECCQAE

DKGACLLPKIETMREKVLASSARQRLRCASIQKFGERAL

KAWSVARLSQKFPKAEFVEVTKLVTDLTKVHKECCHGD

LLECADDRADLAKYICDNQDTISSKLKECCDKPLLEKSH

CIAEVEKDAIPENLPPLTADFAEDKDVCKNYQEAKDAFL

GSFLYEYSRRHPEYAVSVLLRLAKEYEATLEECCAKDDP

HACYSTVFDKLKHLVDEPQNLIKQNCDQFEKLGEYGFQ

NALIVRYTRKVPQVSTPTLVEVSRSLGKVGTRCCTKPESE

RMPCTEDYLSLILNRLCVLHEKTPVSEKVTKCCTESLVN

RRPCFSALTPDETYVPKAFDEKLFTFHADICTLPDTEKQI

KKQTALVELLKHKPKATEEQLKTVMENFVAFVDKCCAA

DDKEACFAVEGPKLVVSTQTALA

LIST OF PLASMIDS AND PRIMERS

**Plasmid Name**	**Description**
pCDF-AA	pCDF-*Mm*PylRS-N346A/C348A
pET-Ftn-TAG	pET-$tRNA^{Pyl}$-FTH1-R63TAG
pET-Ftn-TAG-α	pET-$tRNA^{Pyl}$-FTH1-R63TAG-S(GGGGS)$_3$-αCT-6xHis-tag
pET-Ftn-2xTAG	pET-$tRNA^{Pyl}$-FTH1-R63TAG/E67TAG
pET-Ftn-2xTAG-α	pET-$tRNA^{Pyl}$-FTH1-R63TAG/E67TAG-S(GGGGS)$_3$-αCT-6xHis-tag
pET-α-L3-Ftn	pET-αCT-S(GGGGS)$_3$-FTH1-6xHis-tag
pET-Ftn-L3-α	pET- FTH1-S(GGGGS)$_3$-αCT-6xHis-tag
pET-Ftn-L1-α	pET- FTH1-SGGGGS-αCT-6xHis-tag
pET-Ftn-L2-α	pET- FTH1-S(GGGGS)$_2$-αCT-6xHis-tag
pET-SUMO1	pET-6xHis-tag-TEV-SUMO1
pET-sfGFP	pET-6xHis-tag-TEV-sfGFP

**Primer Name**	**Sequence (5' to 3')**
Ftn-F	ACCACCGCCTCTACCTCACAGGTGCGTCAGAATTAT
Ftn-R	ACTTTCGTTATCGCTATCGCCTAAGGTATG
pET-Ftn-NdeI-F	GATATACATATGACCACCGCCTCTACCTCACAGGTGC
pET-Ftn-SacI-R	GGTGATGGAGCTCACTTTCGTTATCGCTATCGCCTAAG
Ftn-TAG-F	CAGTCTCATGAAGAATAGGAACATGCCGAAAAAC
Ftn-TAG-R	GTTTTTCGGCATGTTCCTATTCTTCATGAGACTGATG
Ftn-2xTAG-F	GAAGAATAGGAACATGCCTAGAAACTGATGAAATTA
Ftn-2xTAG-R	CAGTTTCTAGGCATGTTCCTATTCTTCATGAGAC
pET-Ftn-α-SacI-R	GGTGATGGAGCTCAGATGGCCTGGGGACGAAAAC
pET-α-Ftn-NdeI-F	GAGATATACATATGACGTTTGAGGATTACCTGCACAACGTGGTTTTCG
Ftn-L1-α-F	GATAGCGATAACGAAAGTAGTGGTGGTGGTGGTAGC
Ftn-L2-α-R	ATCCTCAAACGTGCTTCCTCCCCCTCCGCTACCACCACC
Ftn-α-F	AGCGATAACGAAAGTAGTGGTGGTGGTGGTAGCGGAGGGGGAGGA
Ftn-α-R	GTGCAGGTAATCCTCAAACGTGCTACCACCACCACCAC
α-F	ACGTTTGAGGATTACCTGCACAACGTGGTTTTCGTCCCCAGG
L1-α-F	CGTCCCCAGGCCATCTAGCGGAGGAGGAGGCAGC
L1-α-R	GCTAGATGGCCTGGGGACGAAAACCACGTTGTG
L2-F	AGCGGAGGGGGAGGAAGCGGAGGAGGAGGCAGC
L2-α-R	TCCCCCTCCGCTAGATGGCCTGGGGACGAAAACCACGTTG

L3-F	AGTGGTGGTGGTGGTAGCGGAGGGGGAGGAAGCGGAGGAGGAGGCAGC
α-L3-R	ACCACCACCACTAGATGGCCTGGGGACGAAAACCACGTTGTG
L3-α-R	GTAATCCTCAAACGTGCTGCCTCCTCCTCCGCTTCCTCCCCCTCCGCT
L3-Ftn-R	CTGTGAGGTAGAGGCGGTGGTGCTGCCTCCTCCTCC

MATERIALS AND METHOD

Key resources table

REAGENT or RESOURCE	SOURCE	IDENTIFIER
**Chemicals, peptides, and recombinant proteins**		
2-(5-Bromothienyl)-L-alanine (BrThA, **2**)	Chem-Impex (Wood Dale, IL, United States)	Cat#15736
2-Chloro-2,4,6-cycloheptatrien-1-one (**6**)	Sigma-Aldrich (Burlington, MA, United States)	Cat#669571
3-Methyl-histidine (MeH, **3**)	Chem-Impex (Wood Dale, IL, United States)	Cat#11339
3-Methylene-2-norbornanone (**8**)	Sigma-Aldrich (Burlington, MA, United States)	Cat#M46055
4-Thiazolyl-L-alanine (ThzA, **1**)	Chem-Impex (Wood Dale, IL, United States)	Cat#07376
Apoferritin from equine spleen	Sigma-Aldrich (Burlington, MA, United States)	Cat#A3660
Bovine serum albumin (BSA)	Sigma-Aldrich (Burlington, MA, United States)	Cat#A7906
Diethyl benzylidenemalonate (DEBM, **5**)	Sigma-Aldrich (Burlington, MA, United States)	Cat#13471
Diethyl ethylidenemalonate (DEEM, **4**)	Sigma-Aldrich (Burlington, MA, United States)	Cat#D94801
Divinyl sulfone (**10**)	Sigma-Aldrich (Burlington, MA, United States)	Cat#V3700
[D-Ala^6, N-Me-Leu^7]-LH-RH	Sigma-Aldrich (Burlington, MA, United States)	Cat#L6884
Ethyl vinyl sulfone (**9**)	Sigma-Aldrich (Burlington, MA, United States)	Cat#282839
Ferritin from equine spleen	Sigma-Aldrich (Burlington, MA, United States)	Cat#F4503
Glu-C, Sequencing Grade	Promega Corporation (Madison, WI, United States)	Cat#V1651
Insulin, Human Recombinant	Sigma-Aldrich (Burlington, MA, United States)	Cat#91077C
KOD Hot Start Polymerase	Merck KGaA (Darmstadt, Germany)	Cat#71086
Lysozyme (from chicken egg white)	Sigma-Aldrich (Burlington, MA, United States)	Cat#L7651
N-hydroxyethyl acylamide (**7**)	Sigma-Aldrich (Burlington, MA, United States)	Cat#697931
Phenyl vinyl sulfone (**11**)	Sigma-Aldrich (Burlington, MA, United States)	Cat#241717
Recombinant human UBE2V2	SignalChem (Richmond, BC, Canada)	Cat#U239-30H
Recombinant human UBE2N	SignalChem (Richmond, BC, Canada)	Cat#U231-380H
(All restriction enzymes used)	New England Biolabs (Ipswich, MA, USA)	N/A
**Oligonucleotides**		
(All oligonucleotides used)	Genomics Inc. (Taipei, Taiwan)	N/A
**DNA sequencing services**		
(All DNA sequencing services used)	Genomics Inc. (Taipei, Taiwan)	N/A

All chemical reagents were of analytical grade, obtained from commercial suppliers, and used without further purification.

Plasmid construction

All polymerase chain reactions (PCRs) were performed with the KOD Hot Start Polymerase (Merck KGaA). The *Mm*PylRS-AA gene from the pBK-*Mm*PylRS-N346A/C348A plasmid was subcloned into the pCDF-1b vector using restriction enzymes NcoI at the 5' end and EcoRI the at 3' end. The SUMO1 gene with the N-terminal 6xHis-tag and TEV cutting site was installed through overlap-extension PCR. The synthesized 6xHis-tag-TEV-SUMO1 gene was double digested by NdeI and SacI-HF, and then subcloned into pET vector to generate pET-SUMO1. All FTH1 genes and mutants were subcloned into the pET-tRNA^{Pyl} vector using restriction enzymes NdeI at the 5' end and SacI-HF at the 3' end. Plasmid of the Ftn variant bearing a single amber codon, pET-Ftn-TAG, was generated using overlap-extension PCR from pET-tRNA^{Pyl}-Ftn. The primer pairs: pET-Ftn-NdeI-F and Ftn-TAG-R, and Ftn-TAG-F and pET-Ftn-SacI-R, were used to generate an amber mutation in the FTH1 gene. The amplified products were double digested with NdeI and SacI-HF, gel-purified, and ligated back into the pET-tRNA^{Pyl} vector to generate pET-Ftn-TAG. To construct pET-Ftn-2xTAG, the mutations were introduced by overlap extension PCR from pET-Ftn-TAG. The primer pairs: pET-Ftn-NdeI-F and Ftn-2xTAG-R, and Ftn-2xTAG-F and pET-Ftn-SacI-R, were used to yield the Ftn-2xTAG gene. The amplified product was double digested with NdeI and SacI-HF, gel-purified, and ligated back into the pET-tRNA^{Pyl} vector.

To introduce the αCT peptide and S(GGGGS)$_{1\text{-}3}$ (L1-3) linkers to the N- or C-termini of Ftn, the primer pairs: pET-α-Ftn-NdeI-F1 and α-L3-R, L3-F and L3-Ftn-R, and Ftn-F3 and pET-Ftn-SacI-R, were used to generate the α-Ftn gene, pET-Ftn-NdeI-F and Ftn-R1; Ftn-L3-F and L3-αCT-R, αCT-F and pET-Ftn-α-SacI-R, were used to generate the Ftn-α gene; pET-Ftn-NdeI-F and Ftn-L1-R; L-αCT-F and L-αCT-R; αCT-F2 and pET-Ftn-α-SacI-R3, were used to generate the Ftn-L1-α gene; pET-Ftn-NdeI-F and Ftn-L2-R, L2-F and L2-α-R, and α-F and pET-Ftn-α-SacI-R, were used to generate the Ftn-L2-α gene. The amplified products were double digested with NdeI and SacI-HF, gel-purified, and ligated back into pET-tRNA^{Pyl} vector to generate pET-α-Ftn, pET-Ftn-α, pET-Ftn-L1-α, and pET-Ftn-L2-α. pET-Ftn-TAG-α and pET-Ftn-2xTAG-α were generated with the same procedures as for pET-Ftn-TAG and pET-Ftn-2xTAG, but instead, used pET-Ftn-α as their DNA template for overlap-extension PCR.

Expression and purification of recombinant proteins

To express Ftn variants with αCT-fusion, sfGFP and SUMO1, pET-α-Ftn, pET-Ftn-α, pET-Ftn-L1-α, pET-Ftn-L2-α, pET-sfGFP and pET-SUMO1 plasmids containing target genes were respectively transformed into *Escherichia coli* BL21(DE3) competent cells. 25 µL of cells were thawed on ice and mixed with 0.5 µL (80 ng/µL) of each plasmid, separately. The mixtures were left to incubate on ice for 10 min, and heat shocked in a 42°C water bath for 1 min. Cells were then put back on ice to let incubate for 2 min. 1 mL of lysogeny broth (LB) was added to each mixture, which was then incubated at 37°C, shaking at 210 x rpm in an orbital shaking incubator for 1 hr. Cells were centrifuged at 16,000 x g for 1 min to be collected, and plated on LB agar plates with ampicillin (Amp) (100 µg/mL). Plates were incubated overnight at 37°C. A single colony from each plate was selected and inoculated in 5 mL LB broth with Amp (100 µg/mL), separately. Cultures were incubated overnight at 37°C, shaking at 210 x rpm. Fresh LB with Amp was added, making 1:100 dilutions, to expand the cultures at 37°C, again, shaking at 210 x rpm until OD_{600} reaches 0.6. Expression was induced by adding 1 mM IPTG (isopropyl β-D-thiogalactoside) and 50mM ncAA. 500 mL cultures were incubated at 37°C, shaking at 210 x rpm for 12 hr. Cells were then harvested by centrifugation at 6,000 x rpm for 30 min, re-suspended in lysis buffer (20 mM trisaminomethane (Tris) buffer, 100 mM NaCl, pH = 7.5), and lysed by sonication.

To express ncAA-incorporated Ftn variants, pET-tRNA^{Pyl} plasmids containing target genes: pET-Ftn-TAG, pET-Ftn-2xTAG, pET-Ftn-TAG-α, or pET-Ftn-2xTAG-α, were respectively co-transformed alongside pCDF-AA into *E. coli* BL21(DE3) competent cells. 25 µL of cells were thawed on ice and mixed with 0.5 µL (80 ng/µL) of each pET plasmid, separately, alongside 0.5 µL (80 ng/µL) of each pCDF plasmid. The mixtures were left to incubate on ice for 10 min, and heat shocked in a 42°C water bath for 1 min. Cells were then put back on ice to let incubate for 2 min. 1 mL of LB was added to each mixture, which was then incubated at 37°C, shaking at 210 x rpm in an orbital shaking incubator for 1 hr. Cells were centrifuged at 16,000 x g for 1 min to be collected, and plated on LB agar plates with Amp (100 µg/mL) and streptomycin (Sp) (100 µg/mL). Plates were incubated overnight at 37°C. A single colony from each plate was selected and inoculated in 5 mL LB broth with Amp, separately. Cultures were incubated overnight at 37°C, shaking at 210 x rpm. Fresh LB with Amp (100 µg/mL) and Sp (100 µg/mL) was added, making 1:100 dilutions, to expand the cultures at 37°C, again, shaking at 210 x rpm until OD_{600} reaches 0.6-0.8. 200 mL cultures were centrifuged at 4,500 x g for 10 min to be collected, and washed twice with equal volumes of M9 medium (67.8 g Na_2HPO_4, 30 g KH_2PO_4, 10 g NH_4Cl and 5 g NaCl in 1L ddH_2O). Cell pellets were re-cultured in 200 mL of GMML medium (M9 medium supplemented with 1% glycerol, 2 mM $MgSO_4$, 0.1 mM $CaCl_2$) with Amp (100 µg/mL) and Sp (100 µg/mL). Expression was induced by adding 1 mM IPTG and 1 mM ncAA (dissolved in 10-50 mM NaOH solution according to instructions provided by the commercial source). 200 mL cultures were incubated at 37°C, shaking at 210 x rpm for 12 hr. Cells were then harvested by centrifugation at 4,500 x g for 10 min, re-suspended in lysis buffer (20 mM Tris buffer, 100 mM NaCl, pH = 7.5), and lysed by sonication.

The cell lysates were clarified by centrifugation at 10,000 x g for 30 min at 4°C, and supernatants were collected. Ftn variants without αCT-fusion were heated in a hot water bath at 75°C for 15 min, and then left on ice for 30 min. Protein solutions, were, again, centrifugated at 8,000 x g for 15 min at 4°C. Supernatants were concentrated using Amicon® Ultra-15 Centrifugal Filters (MWCO 10 kDa; Merck KGaA), and analyzed by 12% sodium dodecyl sulfate (SDS)-polyacrylamide gel electrophoresis (PAGE). Ftn variants with αCT-fusion were allowed to bind with 1 mL of cOmplete His-Tag Purification Resin (Roche), whereas sfGFP and SUMO1 were allowed to bind with 2 mL of MAM-50 His_NTA resin (EBL Inc.), rotating at 4°C for 1 hr. Mixtures were then subjected to open columns, and washed by 10 CV of lysis buffer and 5 CV of wash buffer (10 mM imidazole, 20 mM Tris, 100 mM NaCl, pH = 7.5). Proteins were then eluted by 5 CV of elution buffer (300 mM imidazole, 20 mM Tris, 100 mM NaCl, pH = 7.5). Both concentration and buffer exchange to lysis buffer of elutions were performed using Amicon® Ultra-15 Centrifugal Filters (MWCO 10 kDa). Samples were digested with 10 equivalents of TEV protease, and analyzed by 12% SDS-PAGE.

Samples containing our proteins of interest were filtered through Minisart® Syringe Filters, Polyethersulfone (PES), Pore Size 0.22 µm, Non-Sterile (Sartorius Inc.) and subjected to purification via size exclusion chromatography (SEC) with the Superdex 200 Increase 10/300 GL column (GE HealthCare Technologies, Inc.) equilibrated in SEC buffer (20 mM Tris, 100 mM NaCl, pH = 7.5). Fractions were analyzed by 12% SDS-PAGE. Those containing our proteins of interest were then concentrated, and stored at -20°C.

Native and SDS-PAGE analyses

Concentrations of proteins were determined by the Quick Start™ Bradford Protein Assay Kit (Bio-Rad Laboratories, Inc.).

Samples for native PAGE analyses were prepared by mixing 10-20 µg of purified protein samples with 4x native dye (100 mM Tris-HCl, 0.02% bromophenol blue, and 20% glycerol, pH = 6.8), obtaining final volumes less than 20 µL. Each sample,

alongside 4 µL of the NativeMark™ Unstained Protein Standard (Thermo Fisher Scientific Inc.), were loaded onto either NuPAGE™ 4 to 12%, Bis-Tris, 1.0 mm, Mini Protein Gels (Thermo Fisher Scientific Inc.) or 7.5% Mini-PROTEAN® TGX™ Precast Protein Gels (Bio-Rad Laboratories, Inc.). Gels were run in 1x TG buffer (25 mM Tris-HCl, 192 mM glycine, pH = 8.5) at 60 V for 6 hr at 4°C.

Samples for SDS-PAGE analyses were prepared by mixing 16 µL of each (diluted) protein sample, respectively, and 4 µL of 5x sample dye (200 mM Tris-HCl, 400 mM dithiothreitol (DTT), 8% SDS, 0.04% bromophenol blue, 40% glycerol, pH = 6.8), obtaining final volumes of 20 µL. Mixtures were heated at 95°C for 5 min and spun down. 10 µL of each denatured sample, alongside 2 µL of the Precision Plus Protein Dual Color Standards (Bio-Rad Laboratories, Inc.), were loaded onto 1.0 mm 4% stacking, 12% resolving SDS-PAGE hand-cast gels. Gels were run in 1x TGS buffer (25 mM Tris-HCl, 192 mM glycine, 0.1% SDS, pH = 8.5 at 120 V for 85 min.

For visualization, gels were gently agitated for 30 min in InstantBlue™ Coomassie Stain (Abcam, Inc.) and destained overnight in ddH_2O.

Characterization of Ftn variants' catalytic activity

To charge Ftn variants with Cu(II), protein solutions were dialyzed in $CuCl_2$ buffer (6 µM $CuCl_2$, 20 mM Tris, 100 mM NaCl, pH = 7.5) at 4°C for 12 hr. Proteins were concentrated using Amicon® Ultra-0.5 Centrifugal Filters (MWCO 10 kDa) to yield concentrations of 2 mg/mL. 50 µL enzymatic reaction mixtures (1 mM substrate: insulin, [D-Ala^6, *N*-Me-Leu^7]-LH-RH, SUMO1, UBE2N, UBE2V2, lysozyme, sfGFP or BSA, 0.04 mM Ftn variants, 2 mM DEEM, **4**, or other chemicals with α,β-unsaturated moiety, **5**-**11**), 20 mM Tris, 100 mM NaCl, pH = 7.5) were prepared, and incubated at 37°C for 12 hr. Control experiments were performed with reaction mixtures using 0.1 mM $CuCl_2$ instead of 0.04 mM Ftn variants.

Reduction of disulfide bonds

To reduce the disulfide bonds of insulin, 30 equivalents of DTT were added to Ftn-α-2x-**2**'s modified insulin product. The mixture was incubated at 37°C for 30 min, and centrifuged at 10,000 x g for 2 min to separate the supernatant from aggregates. The supernatant and aggregates were dissolved, separately, in 20% (v/v) acetonitrile and 0.1% (v/v) formic acid and sent for matrix assisted laser desorption ionization-time of flight mass spectrometry (MALDI-TOF-MS) analyses.

In-gel digestion

DEEM-modified insulin samples were in-gel digested prior to MALDI-TOF-MS/MS analyses. Protein bands were first excised from SDS-PAGE gels, and cut into small pieces. Gel pieces were washed sequentially with, first, 25 mM NH_4HCO_3, then, 40% methanol, and lastly, 100% acetonitrile. Reduction and alkylation of proteins were performed using DTT and iodoacetamide, respectively. Gel pieces were washed again, dried in a vacuum centrifuge, and incubated in 25-30 µL of solution containing 65-100 ng of endoproteinase Glu-C, Sequencing Grade (Promega Corporation) in 25 mM NH_4HCO_3, 10% acetonitrile at 37°C for 12-16 hr. Reactions were quenched by adding 1-2 µL of 5% formic acid. DEEM-modified SUMO1 samples were in-gel digested by trypsin prior to ESI-MS/MS analyses.

ESI-MS(/MS), ICP-MS and MALDI-TOF-MS(/MS) analyses

For electrospray ionization mass spectrometry (ESI-MS) analyses, purified proteins were diluted with 20% (v/v) acetonitrile and 0.1% (v/v) formic acid, and concentrated using Amicon® Ultra-0.5 Centrifugal Filters (MWCO 10 kDa). Aliquots

corresponding to 1 pmol of the pure protein was injected via an ESI source (Waters LockSpray Exact Mass Ionization Source) with a syringe pump (Harvard Apparatus), holding a flow rate of 5 μL/min throughout the analyses. The masses of intact proteins were determined by the Synapt G2 High Definition MS System (Waters). Deconvolutions of acquired spectra to single-charged states of target proteins were determined by using the MaxEnt1 algorithm of the MassLynx 4.1 software (Waters).

For inductively coupled plasma mass spectrometry (ICP-MS) analyses, 100 μL of protein samples were first added to 3 mL of nitric acid 67 - 70%, ULTREX® II (J.T. Baker) in microwave digestion tubes, and digested with the MARS Microwave Digestion System (CEM Corporation). Temperatures was gradually ramped to 130°C in 15 min with the application of 30 W of power, held for 20 min, and then cooled down to room temperature. The digested samples were diluted in ddH_2O to reach 25 mL. Analyses were carried out with the Thermo Scientific XSERIES 2 ICP-MS system (Thermo Fisher Scientific Inc.) Direct nebulization was performed in optimized conditions with an Xs skimmer cone with a platinum sampler cone. The extraction voltage was set at -35 V, radio-frequency power at 510 W, focus voltage at 4.5 V, and the nebulizer gas flow rate at 1.04 L/min. With dwell times of 10 ms, 100 sweeps were acquired for each sample, for each, five replicates were performed.

For MALDI-TOF-MS(/MS) analyses, 0.5 μL of protein samples were carefully mixed with matrix solution (0.5 μL of 5 mg/ml DHB (2,5-dihydroxybenzoic acid) in 30% acetonitrile:0.1% trifluoroacetic acid (TFA)). 0.5 μL of the mixture were deposited onto MTP 600/384 AnchorChip™ Targets (Bruker Daltonics Inc.). Analyses were performed with the Autoflex III MALDI TOF/TOF mass spectrometer (Bruker Daltonics Inc.).

Dynamic light scattering (DLS) analyses

Purified protein samples were diluted to final concentrations of 0.5 mg/mL in lysis buffer, filtered through Minisart® Syringe Filters, Polyethersulfone (PES), Pore Size 0.22 μm, Non-Sterile (Sartorius Inc.), and analyzed with the Zetasizer Nano ZS (Malvern Panalytical Ltd) at 25°C in 1 mL disposable polystyrene cuvettes. Measurements were performed in triplicates. Size distributions derived from intensity distributions were presented.

Field emission gun transmission electron microscope (FEG-TEM) analyses

Purified protein samples were diluted to final concentrations of 75 μg/mL in lysis buffer. Glow discharge of the Formvar/Carbon Supported Copper Grids, size 400 mesh (Sigma-Aldrich) was performed with Emitech K100X (Quorum Technologies Ltd) at 25 mA for 30 sec. 5 μL of each sample were deposited onto the glow-discharged grids, and incubated for 90 sec. Excess solution was then wicked away with filter papers. Grids were then rinsed thrice with 5 μL of ddH_2O, with the solution wicked away immediately. Grids were stained with 5 μL of aqueous, freshly filtered 1% uranyl acetate for 1 min, with the solution then wicked away, and air-dried in a desiccator. Images were collected with the FEI Tecnai G^2 F20 Super TWIN FEG-TEM at an accelerating voltage of 120 kV.

X-crystal structural analysis

After screening ~800 crystallization conditions using the Phoenix RE crystallization robot (Rigaku), crystals of Ftn-1x-**3** (12 mg/mL) were grown at 293K via the sitting-drop vapor diffusion method from the condition: 0.1 M BICINE pH 8.5 and 20% (v/v) PEG 300. Crystals were then transferred to a cryoprotectant solution containing the crystallization buffer supplemented with 12% (v/v) glycerol and flash-cooled in liquid nitrogen. X-ray diffraction data were collected at the beamline 15A1 of the Taiwan Light Source, National Synchrotron Radiation Research Center, Hsinchu, Taiwan. The data were indexed and scaled using HKL2000[1], and the structure were solved by molecular replacement with Phaser within the PHENIX suite[2,3], using a

previously reported human heavy chain ferritin (PDB: 2FHA) structure as the search model. Final models were built through iterative rounds of refinement with phenix.refine and manual re-building with Coot[4]. Structural validation was carried out with MolProbity[5], and structural figures were generated using the PyMOL Molecular Graphics System (Version 1.7.4 Schrödinger, LLC). The validation statistics are provided in Extended Data Table S1.

Cryo-EM sample preparation

For cryo-EM sample preparation, a Vitrobot Mark IV (Thermo Fisher Scientific) was employed, set to a temperature of 4 °C and 100% humidity. Approximately 4 μl of purified sample solution was applied to a glow-discharged Quantifoil R1.2/1.3 UltrAuFoil Holey Gold grids (Quantifoil GmbH, Germany). Following a 10-second incubation, the grids were blotted for 4.0 seconds using filter paper and rapidly plunged into liquid ethane cooled by liquid nitrogen. The cryo-EM grids were subsequently stored in liquid nitrogen until further imaging.

Cryo-EM data acquisition

The cryo-EM grids were initially screened using a 200 kV Talos Arctica transmission electron microscope (Thermo Fisher Scientific) equipped with a Falcon III detector (Thermo Fisher Scientific). Images were acquired in linear mode at a nominal magnification of 120,000×, corresponding to a pixel size of 0.86 Å/pixel, with a defocus setting of -3.0 μm. Grids exhibiting optimal ice thickness and particle distribution were recovered and stored in liquid nitrogen for subsequent data collection on a Titan Krios transmission electron microscope (Thermo Fisher Scientific). High-resolution data collection was automated using EPU-3.6.0 software (Thermo Fisher Scientific) on a 300 kV Titan Krios equipped with an X-FEG electron source. Data acquisition was carried out using a K3 Summit detector (Gatan), equipped with a GIF Bio-Quantum Energy Filter, in super-resolution mode (gun lens 4, spot size 3, C2 aperture 50 μm). The defocus range was set between -0.8 and -1.6 μm, and the Energy Filter slit width was 15 eV. For ferritin variant Ftn-2x-**2** (R63BtA/E67BtA) with or without Cu(II), movie stacks were recorded at a nominal magnification of 130,000×, corresponding to a pixel size of 0.648 Å/pixel (super-resolution: 0.324 Å/pixel). Each movie stack comprised 60 frames of non-gain-normalized TIFF stacks, recorded at a dose rate of ~31.2 $e^{-}/Å^2$ per second. The total exposure time was set to 1.6 s, resulting in an accumulated dose of ~50 $e^{-}/Å^2$ (~0.83 $e^{-}/Å^2$ per frame). For ferritin variant Ftn-2x-**3** (R63MeH/E67MeH) with or without Cu(II), movie stacks were recorded at a nominal magnification of 64,000×, corresponding to a pixel size of 0.668 Å/pixel in super-resolution mode. Each movie stack comprised 50 frames of non-gain-normalized TIFF stacks, recorded at a dose rate of ~26.25 $e^{-}/Å^2$ per second. The total exposure time was set to 2.0 s, resulting in an accumulated dose of ~52.5 $e^{-}/Å^2$ (~1.05 $e^{-}/Å^2$ per frame). A detailed summary of the cryo-EM data acquisition parameters is provided in Extended Data Table S2.

Single-particle image processing and 3D reconstruction

For the ferritin variant Ftn-2x-**2 (**R63BtA/E67BtA), with or without Cu(II), the image stacks recorded in super-resolution mode were processed using the "Patch Motion Correction" function in cryoSPARC[6] with two-fold binning, yielding a pixel size of 0.648 Å/pixel. Contrast transfer function (CTF) estimation was performed on the motion-corrected and dose-weighted images using the "CTF estimation (CTFFind4)" function[7] in cryoSPARC. Initial particle picking was conducted using the "Blob Picker" function on a small dataset, followed by particle extraction with a box size of 420 pixels, rescaled to 128 pixels to expedite calculation. After 2D classification, the selected 2D classes were used as templates for the "Template Picker" function to select particles from the entire dataset. The selected particles were extracted and subjected to further 2D classification. After removing poor-quality class averages through 2D classification, the remaining particles were used for "Ab-initio reconstruction" and

“heterogeneous refinement” with O symmetry. A subset of particles belonging to a 3D class displaying better features was selected and re-extracted with a box size of 420 pixels for subsequent “heterogeneous refinement” and “homogeneous refinement,” both performed with O symmetry. For the ferritin variant Ftn-2x-**3** (R63MeH/E67MeH), with or without Cu(II), image stacks recorded in super-resolution mode were also processed using the “Patch Motion Correction” function in cryoSPARC without binning, resulting in a pixel size of 0.868 Å/pixel. The subsequent cryo-EM single-particle reconstruction procedures followed the same steps as described for the R63BtA/E67BtA variant, with or without Cu(II). Map sharpening and resolution estimation were carried out using cryoSPARC[6]. The overall resolution was determined based on the gold-standard Fourier Shell Correlation (FSC) = 0.143 criterion, and local resolution was calculated within cryoSPARC. The final 3D density maps were visualized using UCSF Chimera[8]. Detailed cryo-EM single-particle reconstruction and image processing procedures are presented in Extended Data Figures S4~S7. Additional statistical data related to the cryo-EM reconstructions can be found in Extended Data Table S2.

Structure determination and model building

For atomic model building based on the cryo-EM map, the atomic structure of the ferritin variant predicted by AlphaFold3[9] was initially fitted into the cryo-EM density map. The mutation sites were manually modified, and conformational differences were adjusted using COOT[10]. The model was then further optimized with the "real-space refinement" feature in PHENIX[11] under default settings. Validation of the atomic model was conducted using the "Comprehensive Validation (cryo-EM)" function in PHENIX. The validation statistics are provided in Extended Data Table S2.

Reference:

1. Otwinowski, Z.; Minor, W., Processing of X-ray diffraction data collected in oscillation mode. (1997) *Methods in enzymology*, 276, 307-26.
2. McCoy, A. J., et al., (2007) Phaser crystallographic software. *Journal of applied crystallography*, 40 (Pt 4), 658-674.
3. Adams, P. D., et al., PHENIX: a comprehensive Python-based system for macromolecular structure solution. (2010) *Acta crystallographica. Section D, Biological crystallography*, 66 (Pt 2), 213-21.
4. Emsley, P.; Lohkamp, B.; Scott, W. G.; Cowtan, K., Features and development of Coot. (2010) *Acta crystallographica. Section D, Biological crystallography*, 66 (Pt 4), 486-501.
5. Williams, C. J., et al., MolProbity: More and better reference data for improved all-atom structure validation. (2018) *Protein science: a publication of the Protein Society*, 27 (1), 293-315.
6. Ali Punjani, John L Rubinstein, David J Fleet & Marcus A Brubaker. cryoSPARC: algorithms for rapid unsupervised cryo-EM structure determination. (2017) *Nat. Methods.* 14: 290–296.
7. Alexis Rohou, Nikolaus Grigorieff. CTFFIND4: Fast and accurate defocus estimation from electron micrographs. 2015. J Struct Biol. 192(2):216-221.
8. Zheng Yang, Keren Lasker, Dina Schneidman-Duhovny, Ben Webb, Conrad C. Huang, Eric F. Pettersen, Thomas D. Goddard, Elaine C. Meng, Andrej Sali, and Thomas E. Ferrin. UCSF Chimera, MODELLER, and IMP: an Integrated Modeling System. (2012) *J Struct Biol.* 179(3): 269–278.
9. Josh Abramson, Jonas Adler, Jack Dunger, Richard Evans, Tim Green, Alexander Pritzel, Olaf Ronneberger, Lindsay Willmore, Andrew J Ballard, Joshua Bambrick, Sebastian W Bodenstein, David A Evans, Chia-Chun Hung, Michael O'Neill, David Reiman, Kathryn Tunyasuvunakool, Zachary Wu, Akvilė Žemgulytė, Eirini Arvaniti, Charles Beattie, Ottavia Bertolli, Alex Bridgland, Alexey Cherepanov, Miles Congreve, Alexander I Cowen-Rivers, Andrew Cowie, Michael Figurnov,

Fabian B Fuchs, Hannah Gladman , Rishub Jain, Yousuf A Khan, Caroline M R Low, Kuba Perlin, Anna Potapenko, Pascal Savy, Sukhdeep Singh, Adrian Stecula, Ashok Thillaisundaram, Catherine Tong, Sergei Yakneen, Ellen D Zhong, Michal Zielinski, Augustin Žídek, Victor Bapst, Pushmeet Kohli, Max Jaderberg, Demis Hassabis, John M Jumper. Accurate structure prediction of biomolecular interactions with AlphaFold 3. (2024) *Nature*. 630(8016):493-5008.

10. Paul Emsley, Kevin Cowtan. Coot: model-building tools for molecular graphics. (2004) *Acta Crystallogr D Biol Crystallogr.* 60(Pt 12 Pt 1): 2126-2132.

11. Pavel V Afonine, Billy K Poon, Randy J Read, Oleg V Sobolev, Thomas C Terwilliger, Alexandre Urzhumtsev, Paul D Adams. Real-space refinement in PHENIX for cryo-EM and crystallography. (2018) *Acta Crystallogr D Struct Biol.* 74(Pt 6):531-544.

SUPPLEMENTARY TABLES

Table S1. X-ray Data collection and refinement statistics

		**Ftn-1x-3 Ferritin-R63MeHis**
**Data collection**		
Wavelength (Å)		1.0
Space group		$P4_22_12$
Resolution (Å)		30-2.28 (2.36-2.28)*
Unit cell dimensions,	a, b, c (Å)	219.17, 219.17, 219.17
	α, β, δ (°)	90.0, 90.0, 90.0
Total observations		1,160,308
Unique reflections		159,010 (14,962)
Multiplicity		7.3 (7.2)
Completeness (%)		97.5 (93.1)
$I/\sigma(I)$		8.4 (2.0)
R_{merge}		0.207 (0.783)
R_{meas}		0.223 (0.843)
R_{pim}		0.080 (0.305)
$CC_{1/2}$		(0.949)
CC*		(0.987)
**Refinement**		
Resolution (Å)		29.885 - 2.28
Reflections [> 0σ(F)], working/test		152,641 (14,539)/ 1,980 (188)
R_{work}/R_{free}		0.2387 (0.2956)/ 0.2717 (0.3669)
R.m.s.d., bond lengths (Å)/ angles (°)		0.003/0.58
Average B factor (Å^2)/No. of atoms		
All non-hydrogen atoms		41.14/19,202
Protein		39.99/17,080
Ligands		55.53/118
Solvent		50.12/2004
Ramachandran plot, residues in (%)		
Favored regions		98.25
Allowed regions		1.75
Outliers		0.00
Rotamer outliers (%)		0.75
Clashscore		1.73
**PDB code**		**9JIU**

*Statistics for the highest-resolution shell are shown in parentheses.

Table S2. Cryo-EM data collection, refinement and validation statistics

	Ftn-2x-**2** (R63BtA/E67BtA) (EMD-61726) (PDB-9JQB)	Ftn-2x-**2** (R63BtA/E67BtA) with Cu(II) (EMD-61727) (PDB-9JQC)	Ftn-2x-**3** R63MeH/R67MeH (EMD-61728) (PDB-9JQD)	Ftn-2x-**3** R63MeH/R67MeH with Cu(II) (EMD-61729) (PDB-9JQE)
**Data collection**				
EM equipment	Titan Krios	Titan Krios	Titan Krios	Titan Krios
Voltage (kV)	300	300	300	300
Cs (mm)	2.7	2.7	2.7	2.7
Magnification (nominal)	130,000	130,000	64,000	64,000
Detector	K3	K3	K3	K3
Pixel size (Å)	0.648 (Counting)	0.648 (Counting)	0.668 (Super-resolution)	0.668 (Super-resolution)
Electron exposure (e^{-}/Å^2)	~ 50	~ 50	~ 50	~ 50
Exposure time (s)	1.6	1.6	2.0	2.0
Frames (no.)	60	60	50	50
Defocus range (μm)	-0.22 ~ -2.30	-0.20 ~ -2.88	-0.20 ~ -3.0	-0.20 ~ -2.74
**Reconstruction**				
Software	cryoSPARC	cryoSPARC	cryoSPARC	cryoSPARC
Micrographs stacks (no.)	4,946	4,717	7,338	3,623
Final particle images (no.)	669,487	472,193	1,566,061	2,502,206
Symmetry imposed	O	O	O	O
Map final resolution (Å) †	1.78	1.73	1.82	1.89
Map sharpening B-factor (Å2)	-66.7	-60.8	-69.4	-78.7
**Atomic modeling**				
Software	Coot & Phenix	Coot & Phenix	Coot & Phenix	Coot & Phenix
Number of protein residues	4,080	4,080	4,080	4,080
Number of ions	24	24	48	48
Number of atoms	37,416	36,864	37,104	36,888
Map CC (around atoms) *	0.88	0.89	0.89	0.88
RMSD bond lengths (Å)	0.005	0.004	0.004	0.004
RMSD bond angles (°)	0.930	0.912	0.830	0.814
Clash score *	1.98	3.89	3.01	3.33
Ramachandran favored (%) *	98.78	98.78	98.78	98.78

Ramachandran allowed (%) *	1.22	1.22	1.22	1.22
Ramachandran outliers (%) *	0	0	0	0
Rotamer outliers (%) *	0	0	0	0
C_β deviations *	0	0	0	0
MolProbity score *	0.97	1.18	1.09	1.12

†According to FSC=0.143

* According to the criterion of Chen *et al.*, 2010 (Chen et al., 2010).

Reference:

V. B. Chen, W. B. Arendall, J. J. Headd, D. A. Keedy, R. M. Immormino, G. J. Kapral, L. W. Murray, J. S. Richardson, D. C. Richardson, MolProbity: all-atom structure validation for macromolecular crystallography. Acta Crystallogr. D Biol. Crystallogr. 66, 12–21 (2010).

SUPPLEMENTARY FIGURES

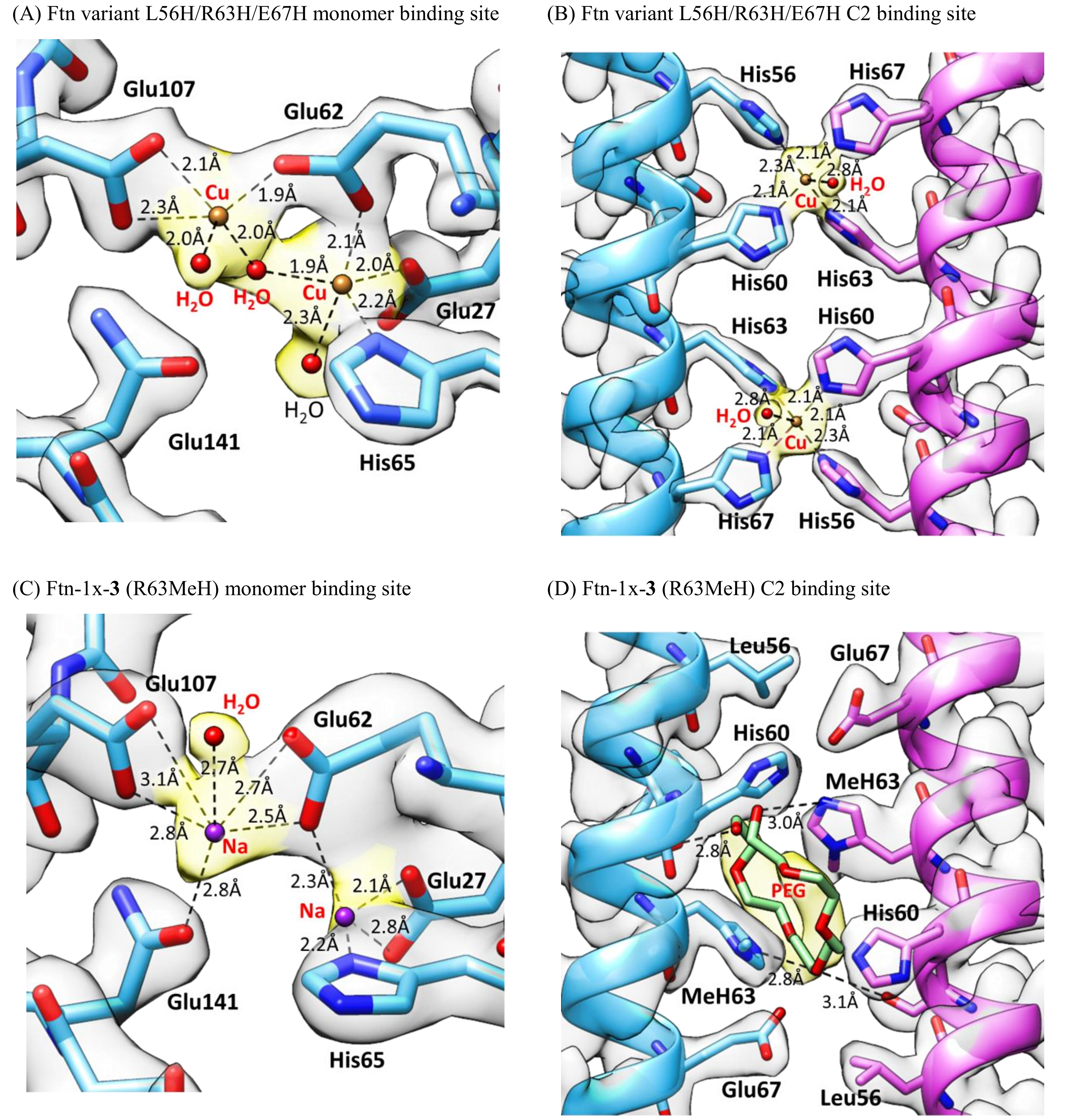

Figure S1. The metal binding sites of ferritin variants. X-ray crystal structures of (A) monomer metal binding site and (B) C2 binding site of Ftn variant L56H/R63H/E67H (PDB:4DYX); x-ray crystal structures of (C) monomer metal binding site and (D) C2 binding site of Ftn-1x-**3** (R63MeH) (PDB code: 9JIU). Protein residues are depicted as light blue sticks, sodium ions in purple, copper in yellow, and iron in orange. The 1.0σ electron densities are shown in transparent gray overlaid with the stick models of residues.

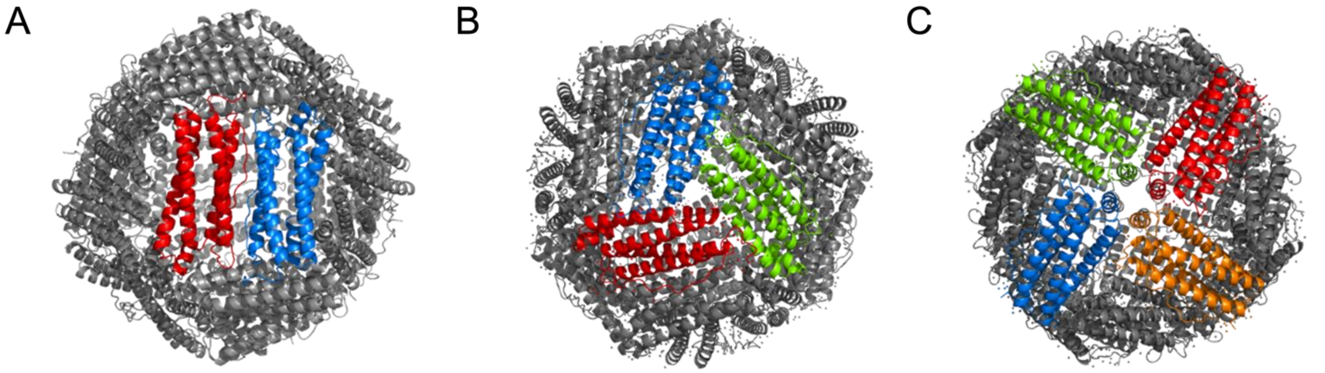

Figure S2. Every 24 FTH1 subunits are able to self-assemble to form an Ftn (PDB code: 1MFR) cage, as shown, which exhibits (A) 12 C_2 axes, (B) 8 C_3 axes and (C) 6 C_4 axes.

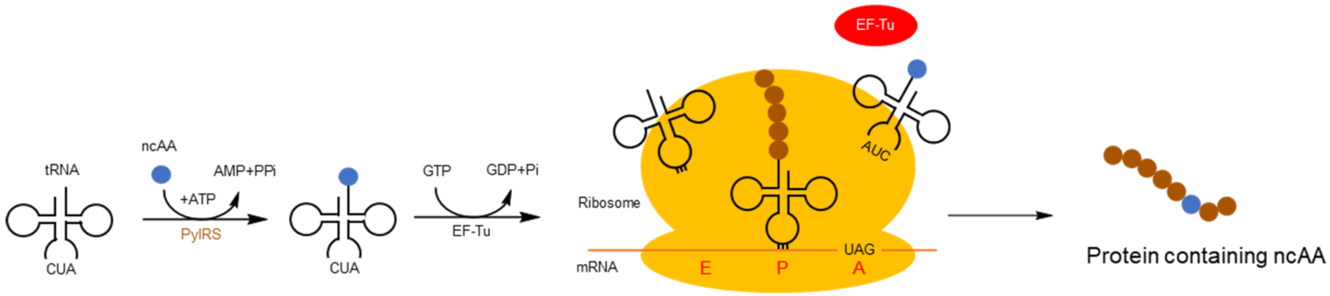

Figure S3. Utilizing the genetic expansion approach, ncAA incorporation could be carried out via amber codon suppression with a pyrrolysyl-tRNA synthetase (PylRS)•pyrrolysine transfer RNA ($tRNA^{Pyl}$) pair, in which the PylRS is able to first charge its cognate tRNA with an ncAA of interest, and the charged tRNA is then able to recognize the amber stop codon to incorporate the ncAA through ribosomal synthesis.

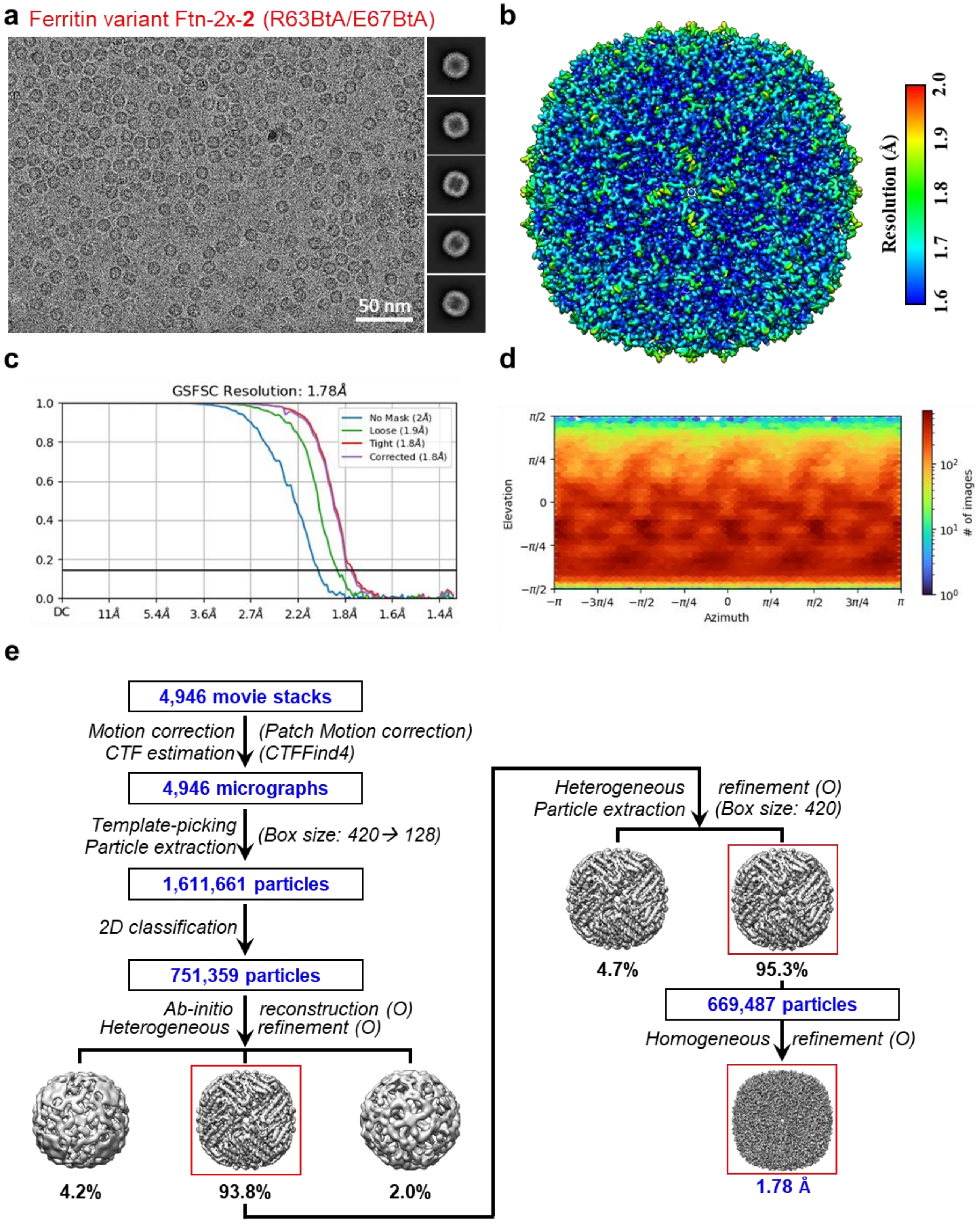

Figure S4. Cryo-EM structure determination of Ferritin variant Ftn-2x-2. a. Representative motion-corrected cryo-EM images and representative 2D class averages. **b.** Local resolution analysis of the cryo-EM map. The map was color-coded

based on their respective local resolution values. **c.** Gold-standard FSC curves (FSC= 0.143). **d.** The angular distributions of all particle projections in the final 3D reconstructions. The heat maps depict the number of particles observed for each viewing angle. Regions colored in red indicate a higher particle count, indicating a more frequent occurrence of those specific viewing angles. **e.** The flow chart for the cryo-EM data processing of Ferritin variant Ftn-2x-**2** (R63BtA/E67BtA). PDB code: 9JQB.

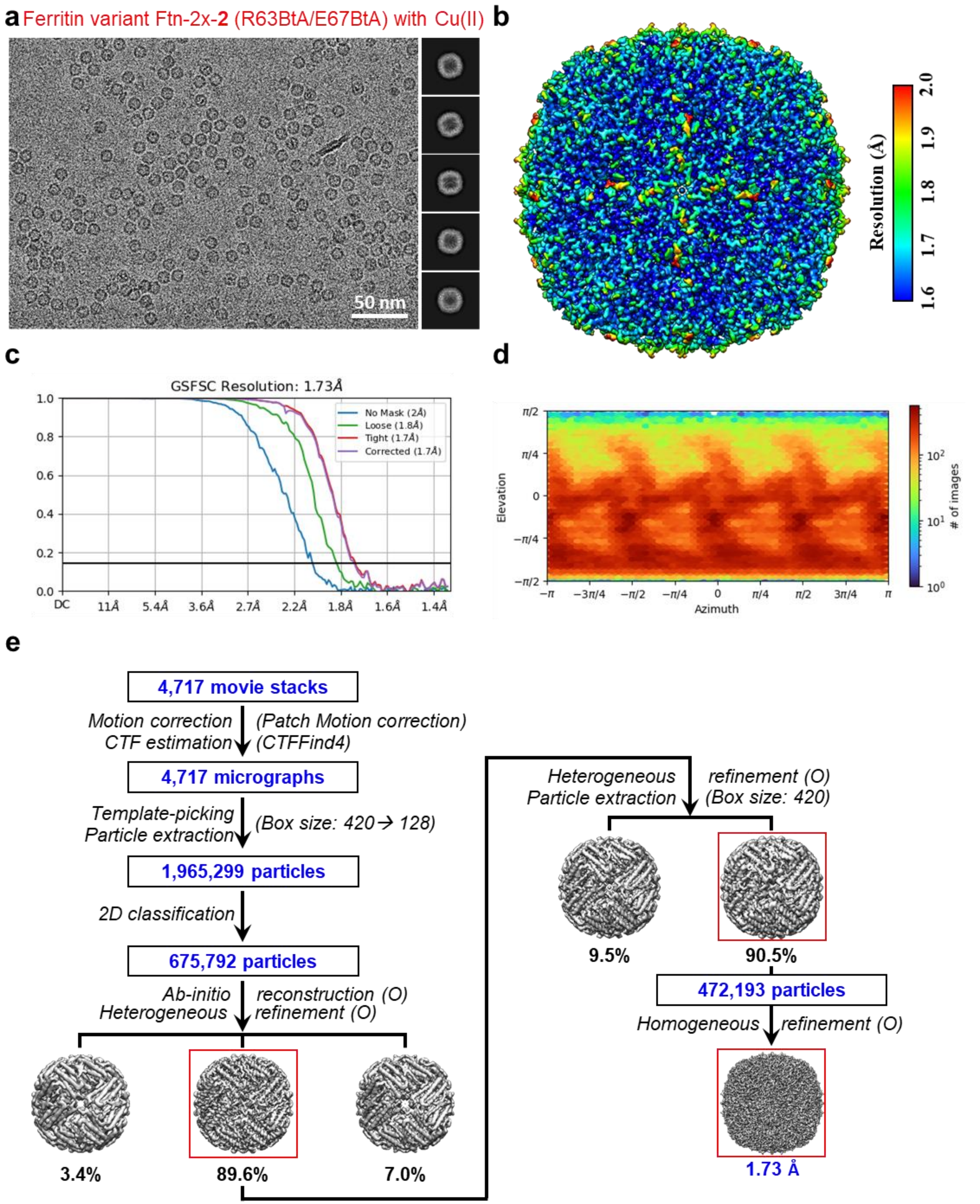

Figure. S5. Cryo-EM structure determination of Ferritin variant with Ftn-2x-2 with Cu(II). a. Representative motion-corrected cryo-EM images and representative 2D class averages. **b.** Local resolution analysis of the cryo-EM map. The map was color-coded based on their respective local resolution values. **c.** Gold-standard FSC curves (FSC= 0.143). **d.** The angular

distributions of all particle projections in the final 3D reconstructions. The heat maps depict the number of particles observed for each viewing angle. Regions colored in red indicate a higher particle count, indicating a more frequent occurrence of those specific viewing angles. **e.** The flow chart for the cryo-EM data processing of Ferritin variant Ftn-2x-**2** (R63BtA/E67BtA) with Cu(II). PDB code: 9JQC.

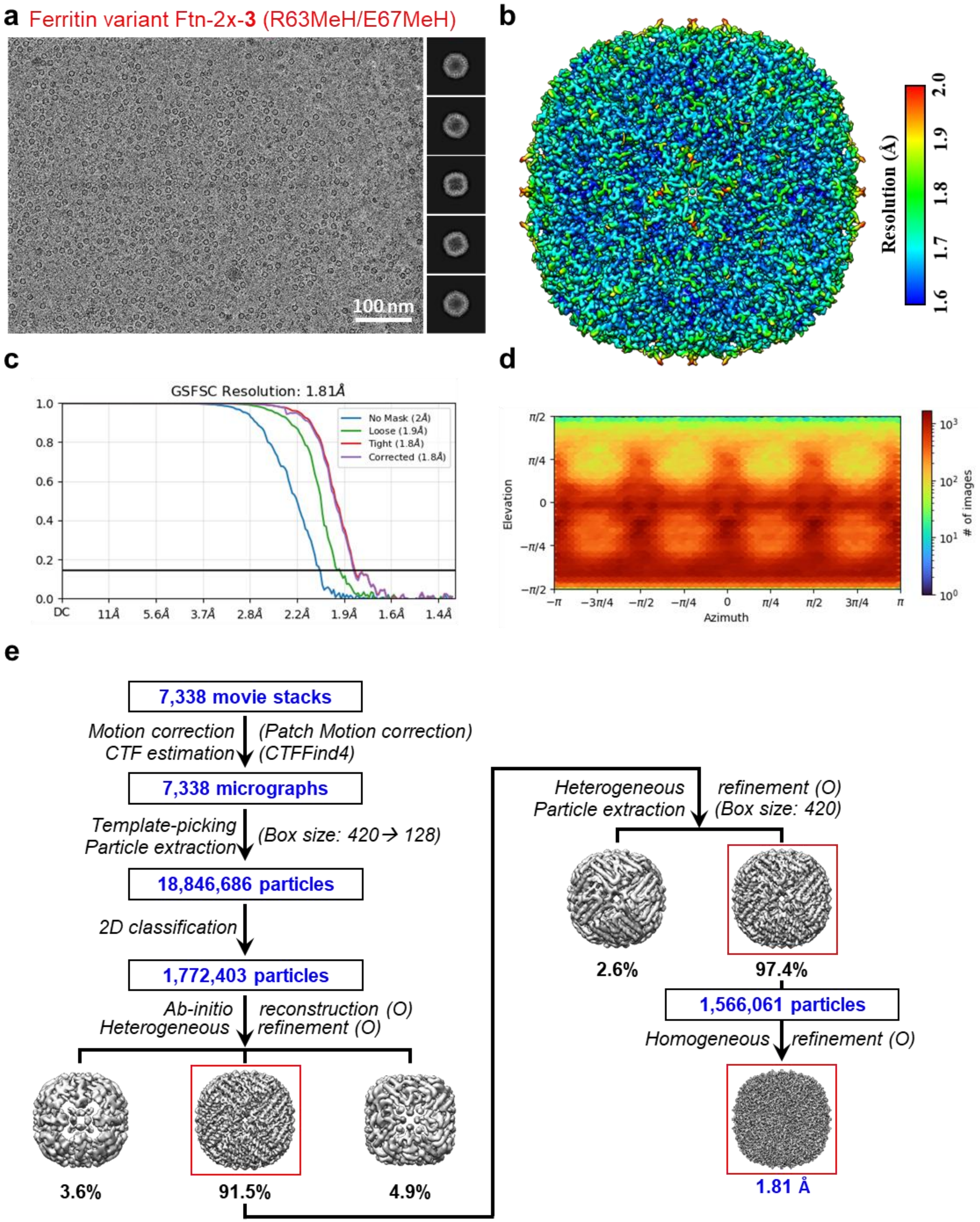

Figure S6. Cryo-EM structure determination of Ferritin variant Ftn-2x-3. a. Representative motion-corrected cryo-EM images and representative 2D class averages. **b.** Local resolution analysis of the cryo-EM map. The map was color-coded based on their respective local resolution values. **c.** Gold-standard FSC curves (FSC= 0.143). **d.** The angular distributions of all particle projections in the final 3D reconstructions. The heat maps depict the number of particles observed for each

viewing angle. Regions colored in red indicate a higher particle count, indicating a more frequent occurrence of those specific viewing angles. **e.** The flow chart for the cryo-EM data processing of Ferritin variant Ftn-2x-**3** (R63MeH/E67MeH). PDB code: 9JQD.

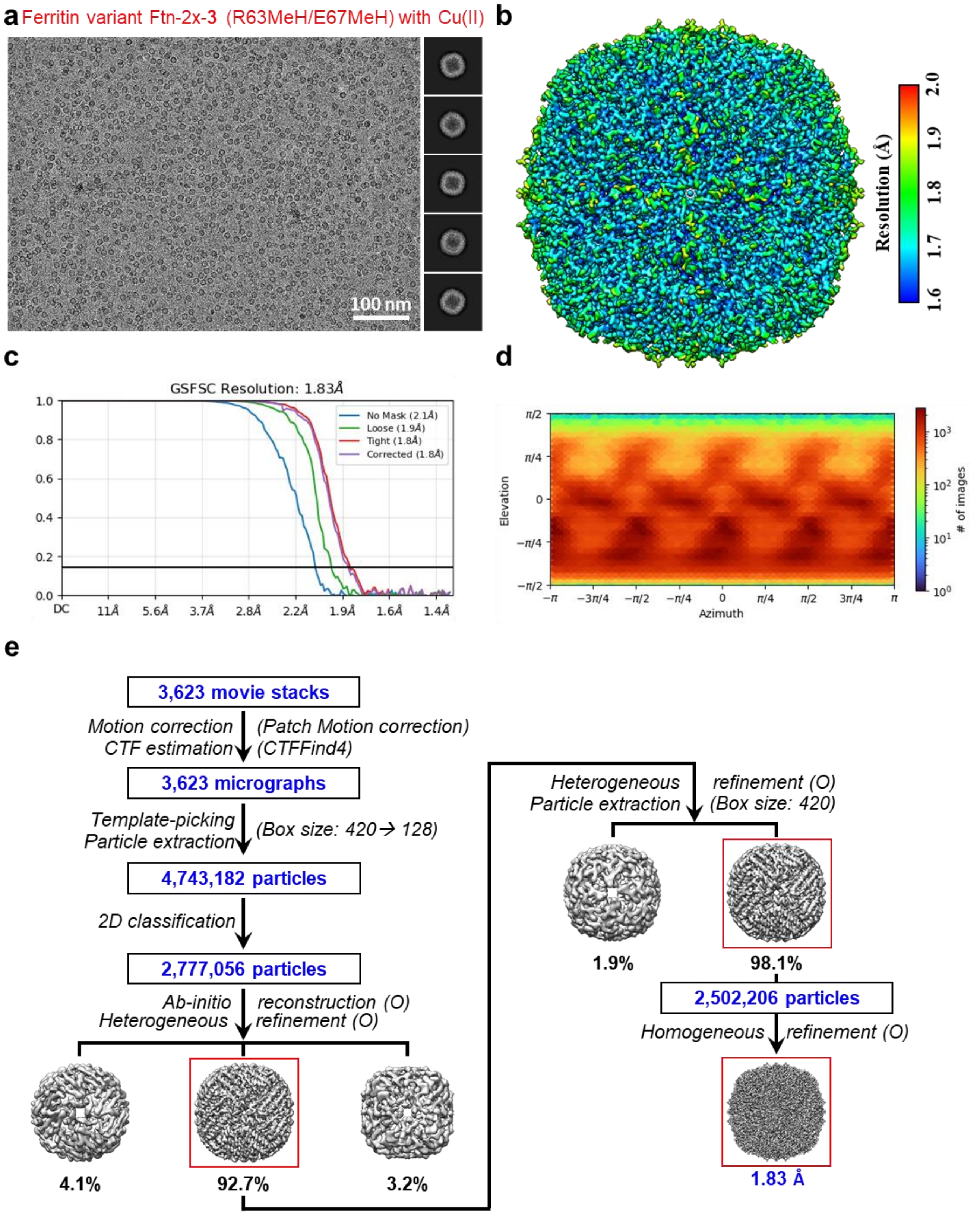

Figure S7. Cryo-EM structure determination of Ferritin variant Ftn-2x-3 with Cu(II). a. Representative motion-corrected cryo-EM images and representative 2D class averages. **b.** Local resolution analysis of the cryo-EM map. The map was color-coded based on their respective local resolution values. **c.** Gold-standard FSC curves (FSC= 0.143). **d.** The angular distributions of all particle projections in the final 3D reconstructions. The heat maps depict the number of particles observed

for each viewing angle. Regions colored in red indicate a higher particle count, indicating a more frequent occurrence of those specific viewing angles. **e.** The flow chart for the cryo-EM data processing of Ferritin variant Ftn-2x-**3** (R63MeH/E67MeH) with Cu(II). PDB code: 9JQE.

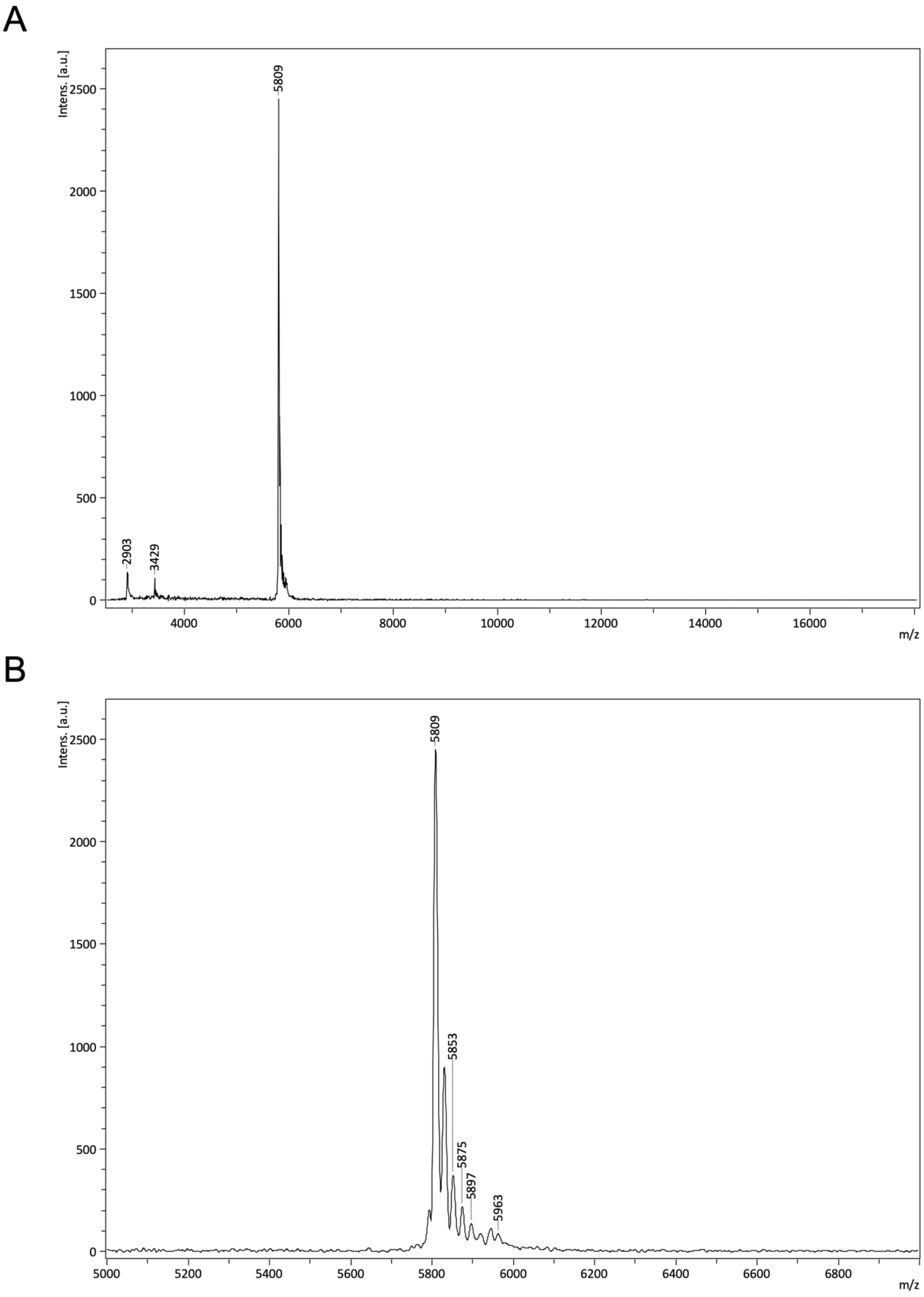

Figure S8. (A) MALDI-TOF-MS analysis of the human insulin protein (molecular weight (MW) = 5,808 Da), (B) zoomed in, with the most significant peak in the mass spectrum representing 5,809 Da.

A

B

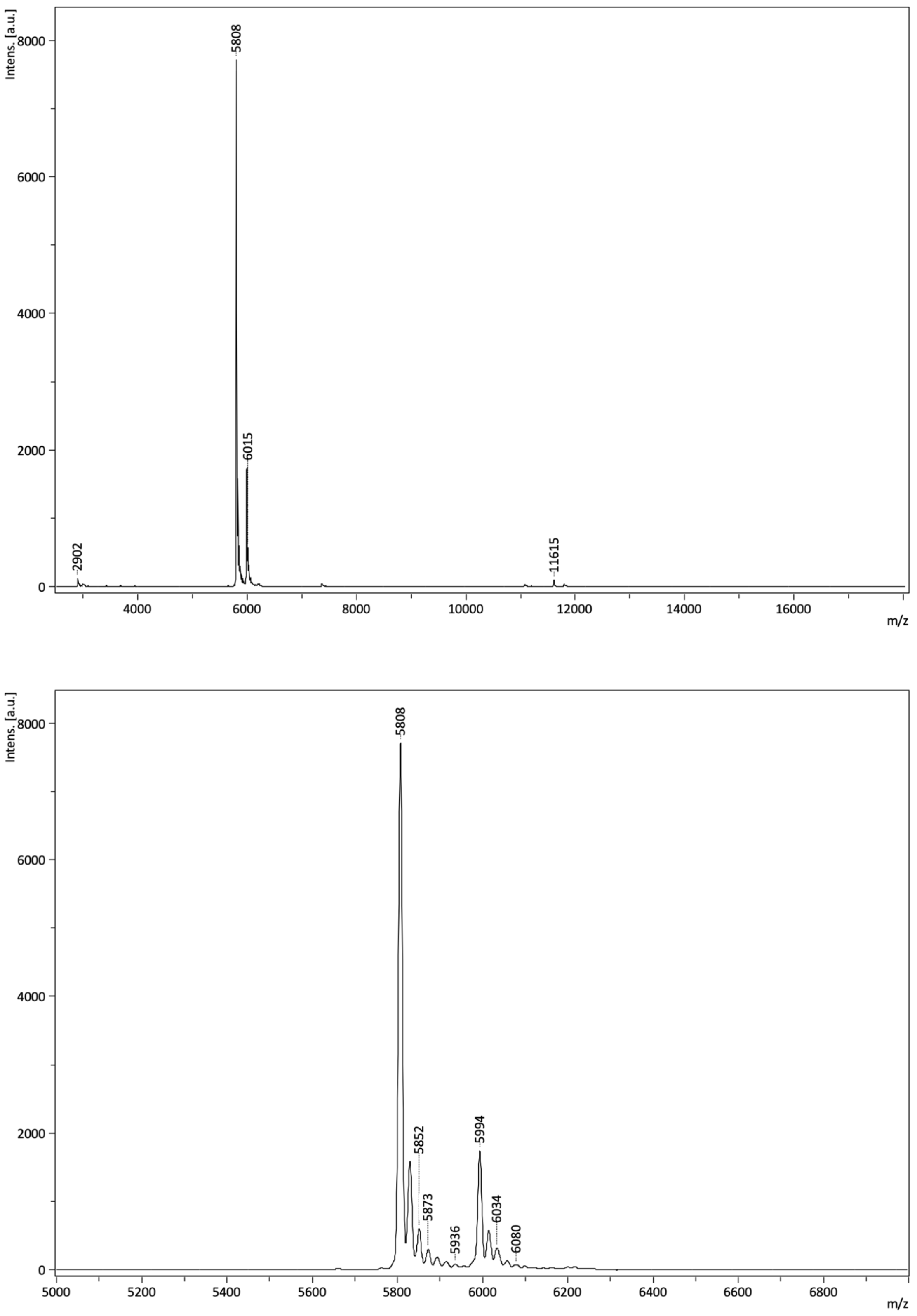

Figure S9. (A) MALDI-TOF-MS analysis of $CuCl_2$-treated Ftn-1x-**3**'s modified insulin product, (B) zoomed in, with only two significant peaks identified, representing 5,808 Da (insulin MW = 5,808 Da) and 5,994 Da (insulin-1x-DEEM MW = 5,994 Da; conversion rate (CVR) = 20%), respectively.

A

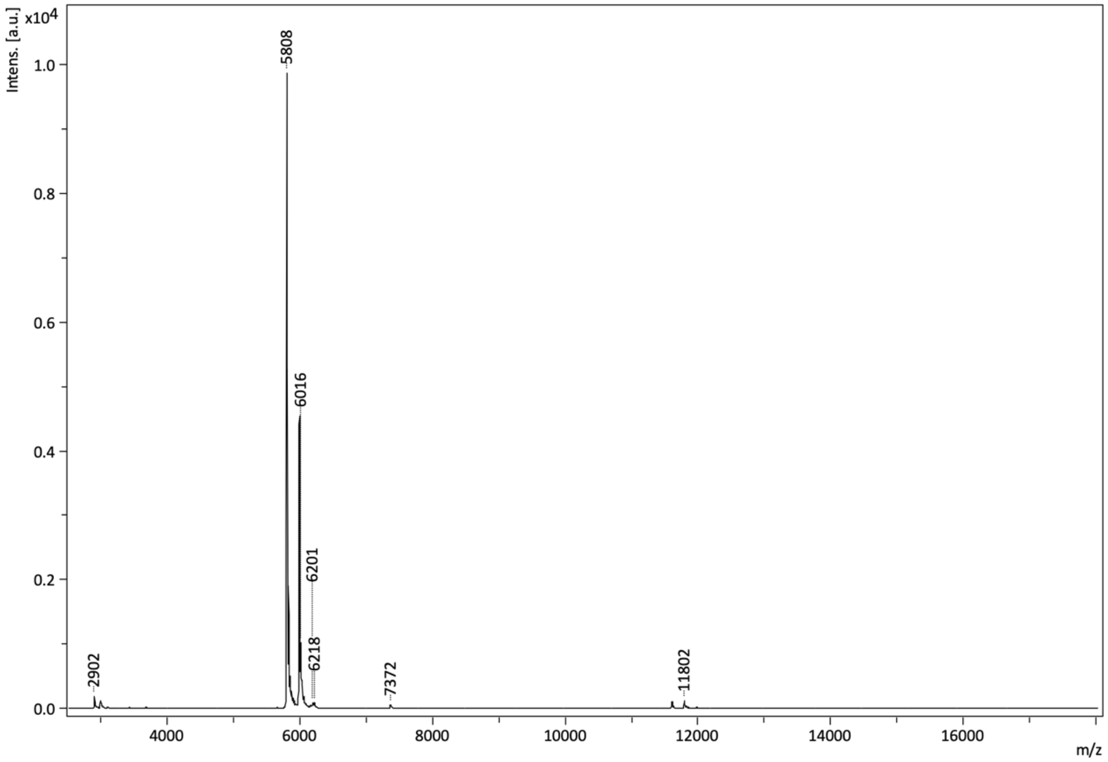

B

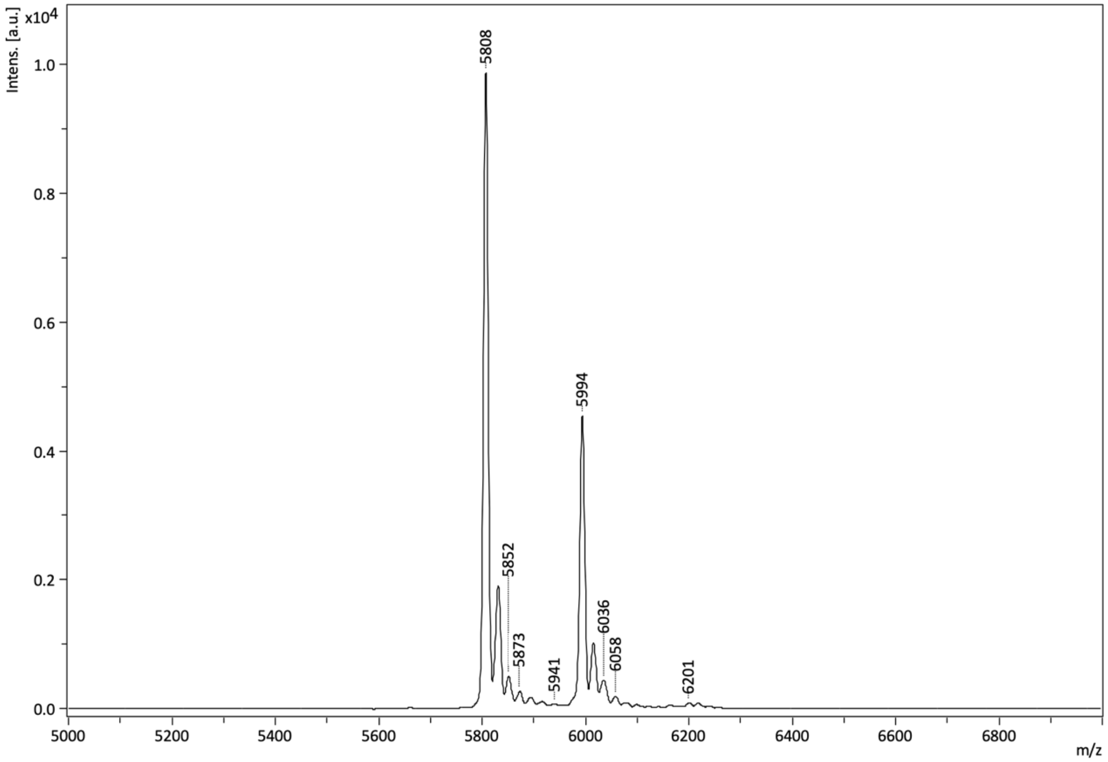

Figure S10. (A) MALDI-TOF-MS analysis of $CuCl_2$-treated Ftn-2x-**2**'s modified insulin product, (B) zoomed in, again, with only two significant peaks identified, representing insulin (MW = 5,808 Da) and insulin-1x-DEEM (MW = 5,994 Da; CVR = 33%), respectively.

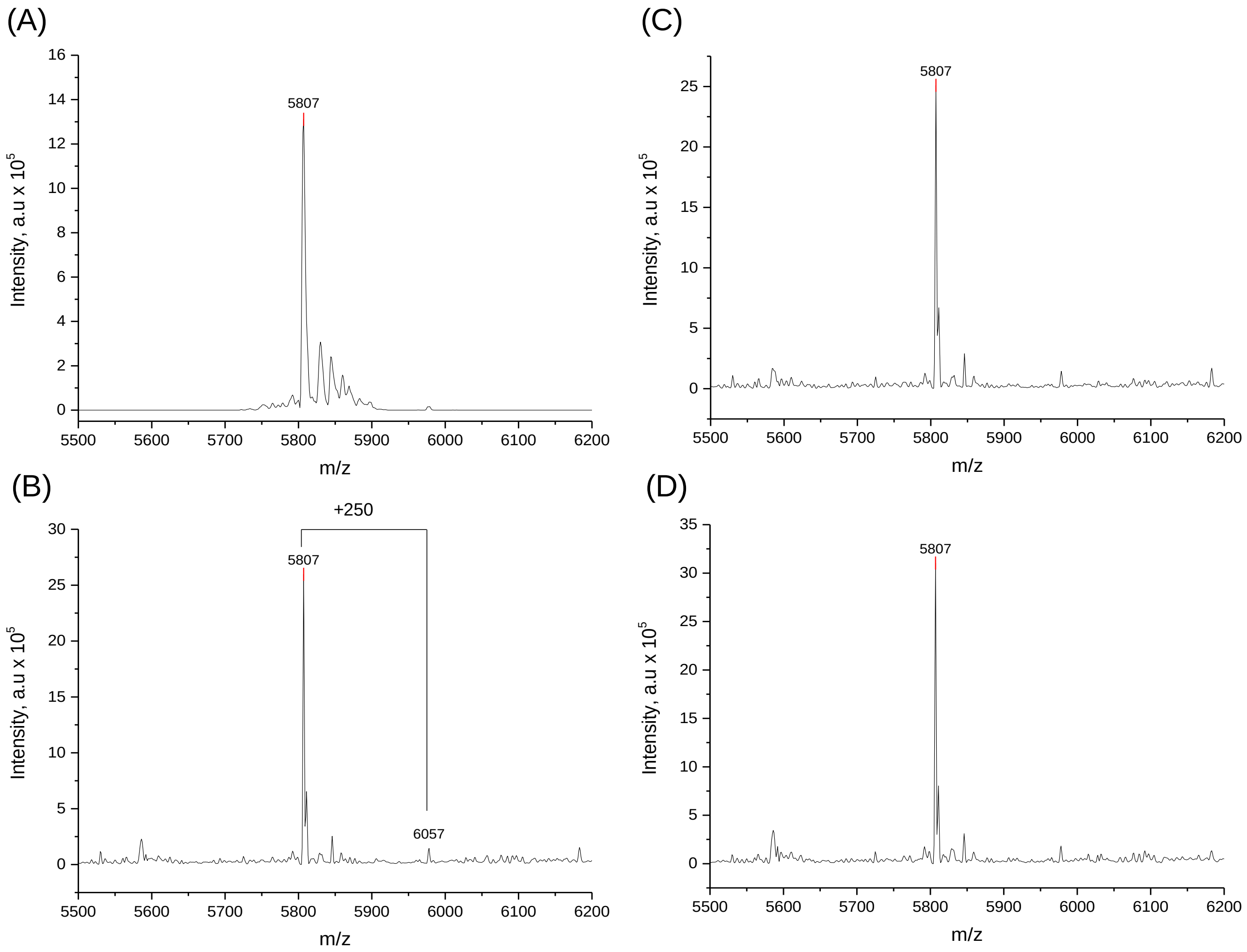

Figure S11. ESI-MS analyses of (A) insulin, and $CuCl_2$-treated Ftn-α-2x-**2**'s modified insulin products with (B) DBEM (**5**; MW = 248 Da), (C) 2-chloro-2,4,6-cycloheptatrien-1-one (**6**; MW = 140 Da) and (D) *N*-hydroxyethyl acylamide (**7**; MW = 115 Da) treatments, respectively, in which (A, C-D) single large peaks at 5,807 Da represent insulin alone, whereas (B) an additional significant peak at 6,057 Da represents insulin-1x-DBEM (CVR = 9.0%).

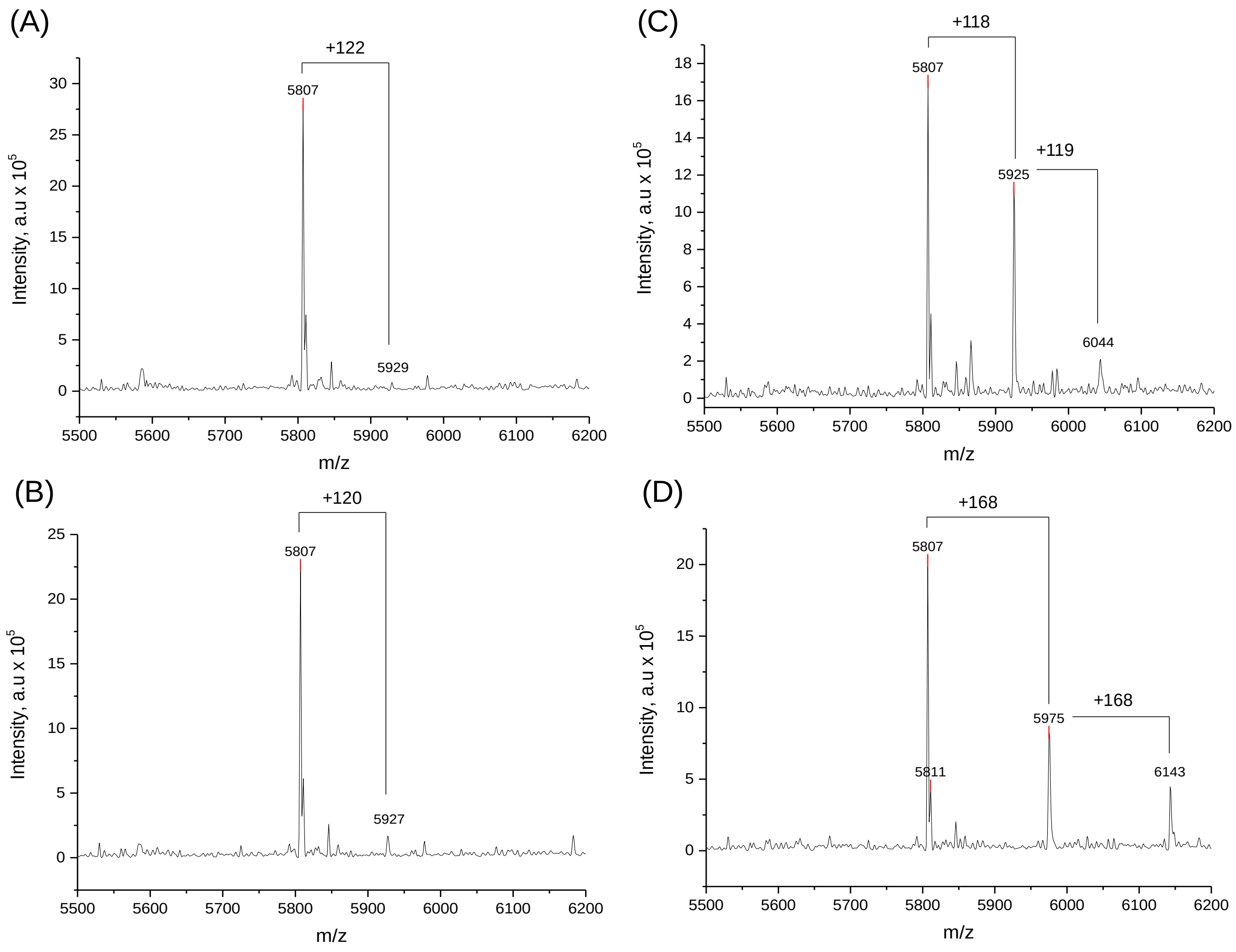

Figure S12. ESI-MS analyses of and $CuCl_2$-treated Ftn-α-2x-**2**'s modified insulin products with (A) 3-methylene-2-norbornanone (**8**; MW = 122 Da), (B) ethyl vinyl sulfone (**9**; MW = 120 Da), (C) divinyl sulfone (**10**; MW = 118 Da) and (D) phenyl vinyl sulfone (**11**; MW = 168 Da) treatments, respectively, in which the large peaks at 5,807 Da represent the insulin protein, whereas additional significant peaks represent (A) insulin-1x-**8** (MW = 5,929 Da; CVR = 3.0%), (B) insulin-1x-**9** (MW = 5,927 Da; CVR = 9%). (C) insulin-1x-**10** (MW = 5,925 Da; CVR = 37%) and insulin-2x-**10** (MW = 6,044 Da; CVR = 7%), (D) insulin-1x-**11** (MW = 5,975 Da; CVR = 24%) and insulin-2x-**11** (MW = 6,143 Da; CVR = 13%).

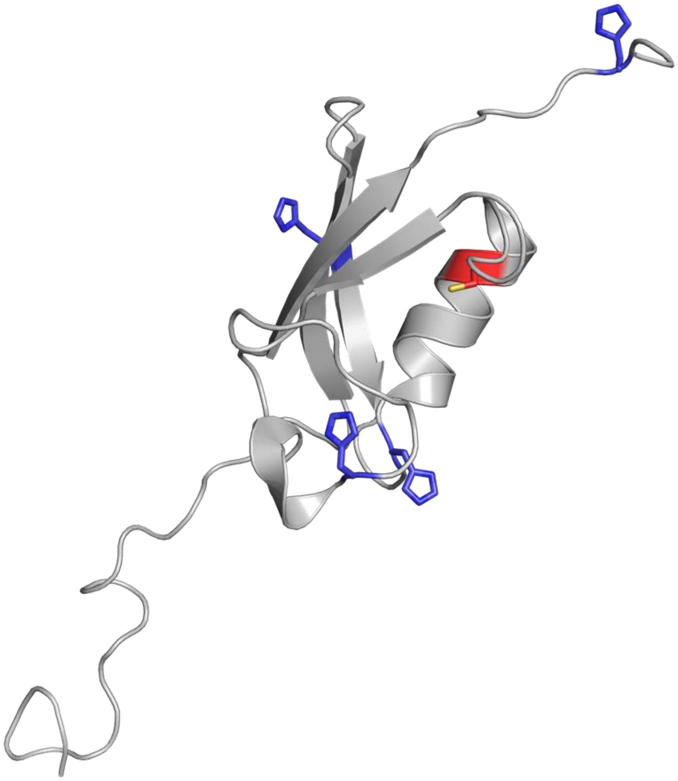

Figure S13. SUMO1 (PDB code: 1A5R) contains 96 amino acids with three histidine (blue) and one cysteine (red) residues.

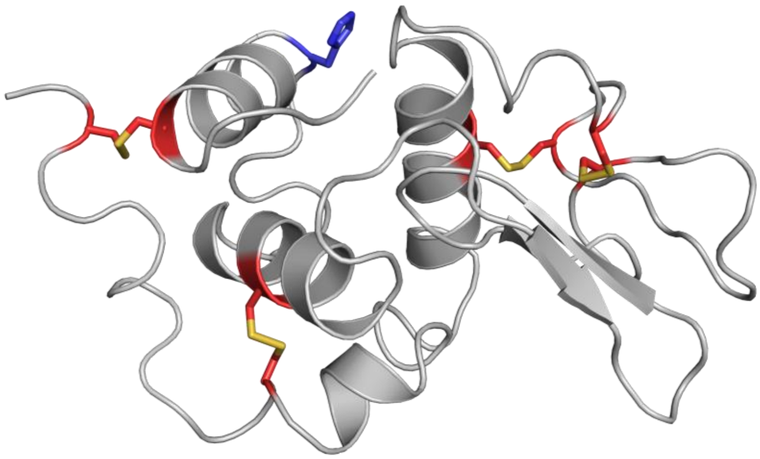

Figure S14. The lysozyme (PDB code: 132L) contains 147 amino with one histidine (blue) and nine cysteine (red) residues, with only eight of the latter shown in this N-terminally truncated protein for crystallization.

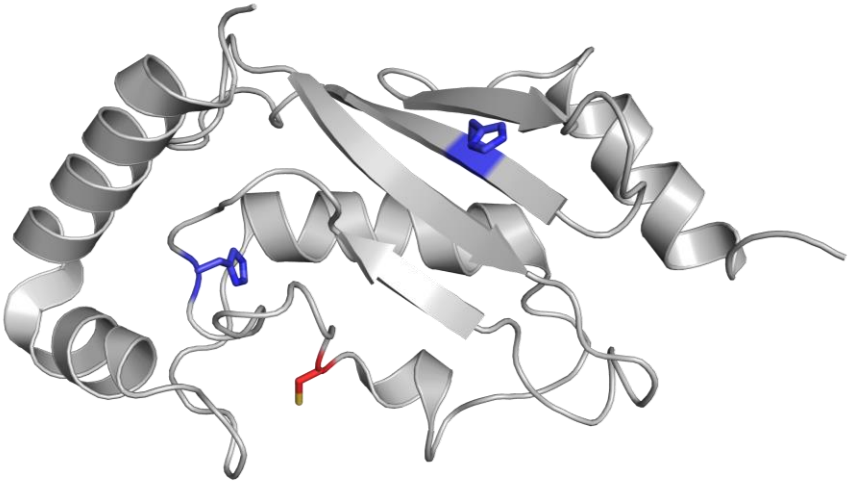

Figure S15. UBE2N (PDB code: 1J7D_chainB) contains 152 amino acids with two histidine (blue) and one cysteine (red) residues.

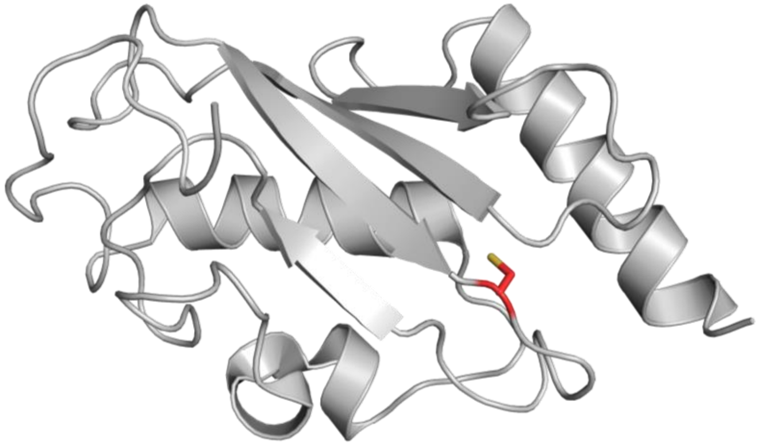

Figure S16. UBE2V2 (PDB code: 1J74) contains 145 amino acids with no histidine and one cysteine (red) residue.

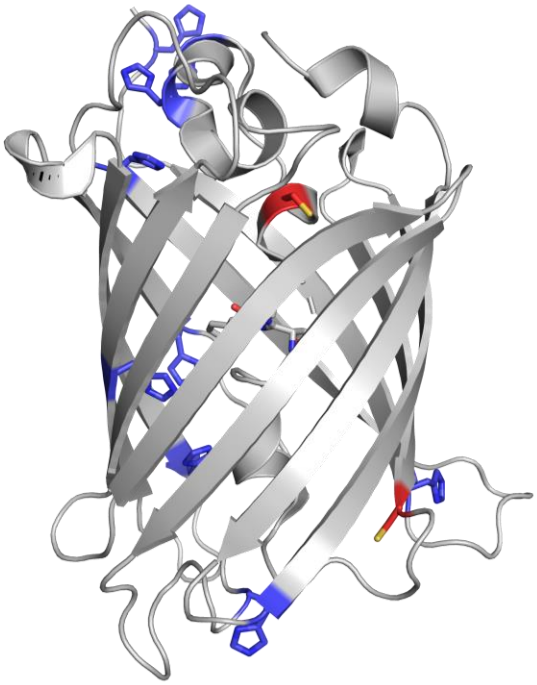

Figure S17. sfGFP contains (PDB code: 2B3P) 242 amino acids with ten histidine (blue) and two cysteine (red) residues.

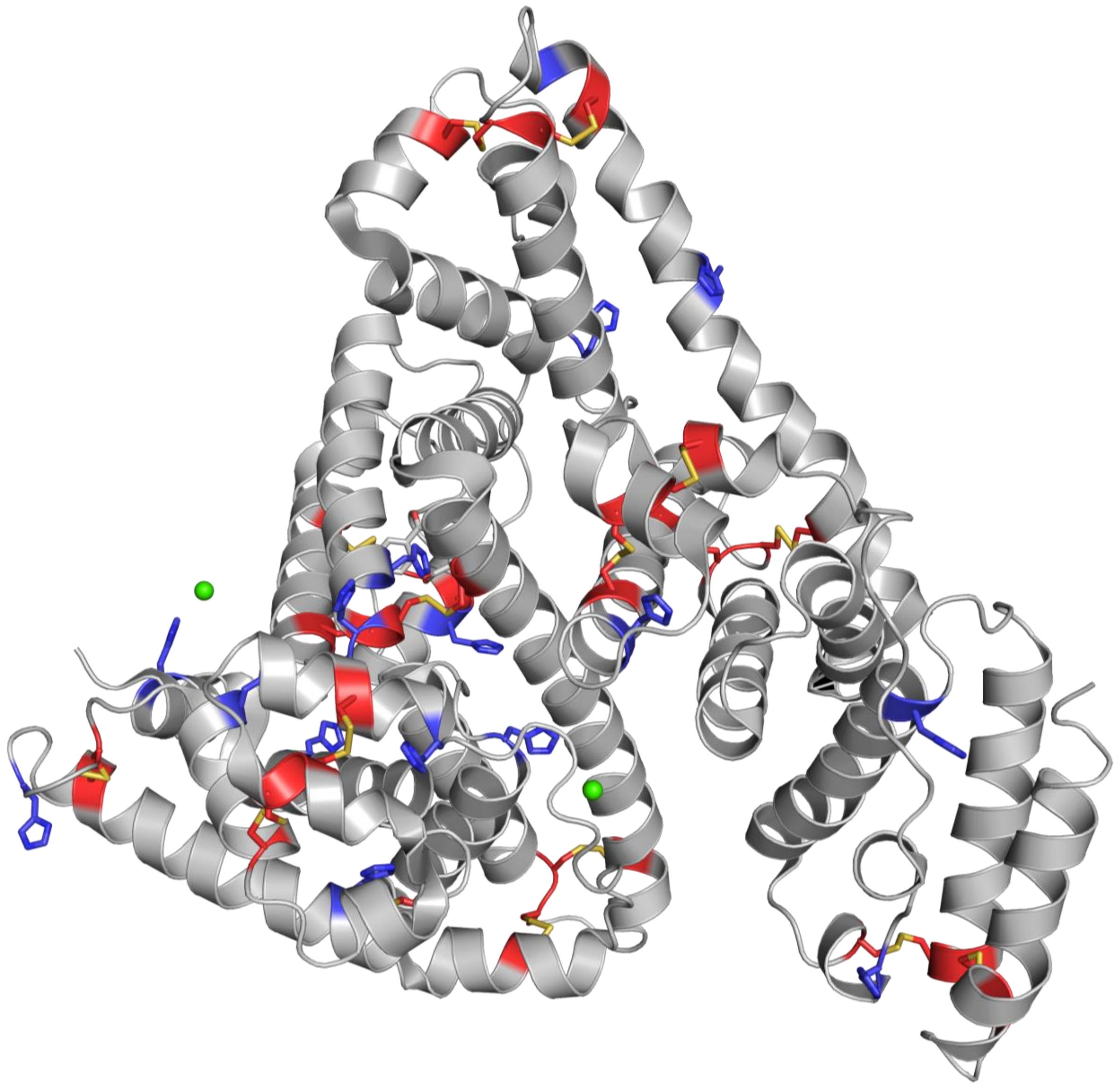

Figure S18. BSA (PDB code: 6QS9_chainA) contains 607 amino acids with 16 histidine (blue) and 35 cysteine (red) residues.

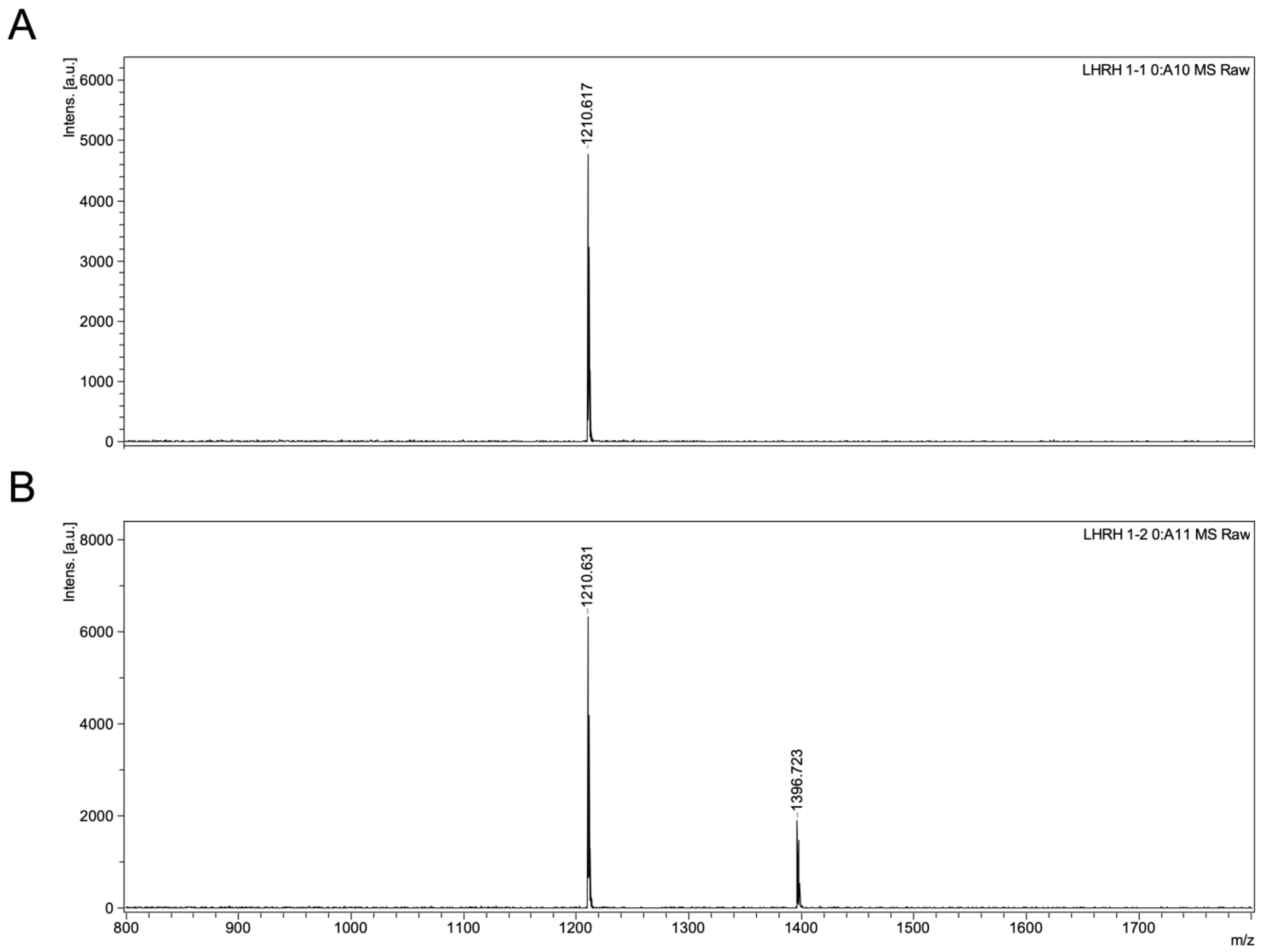

Figure S19. MALDI-TOF-MS analyses of (A) [D-Ala^6, *N*-Me-Leu^7]-LH-RH (MW = 1210.4 Da), and (B) $CuCl_2$-treated Ftn-2x-**2**'s modified [D-Ala^6, *N*-Me-Leu^7]-LH-RH product, in which a single large peak at 1,210.6 Da is observed in the former, representing the LH-RH analog alone, and the additional significant peak at 1,396.7 Da in the latter represents [D-Ala^6, *N*-Me-Leu^7]-LH-RH-1x-DEEM (MW = 1,396.6 Da; CVR = 25%).

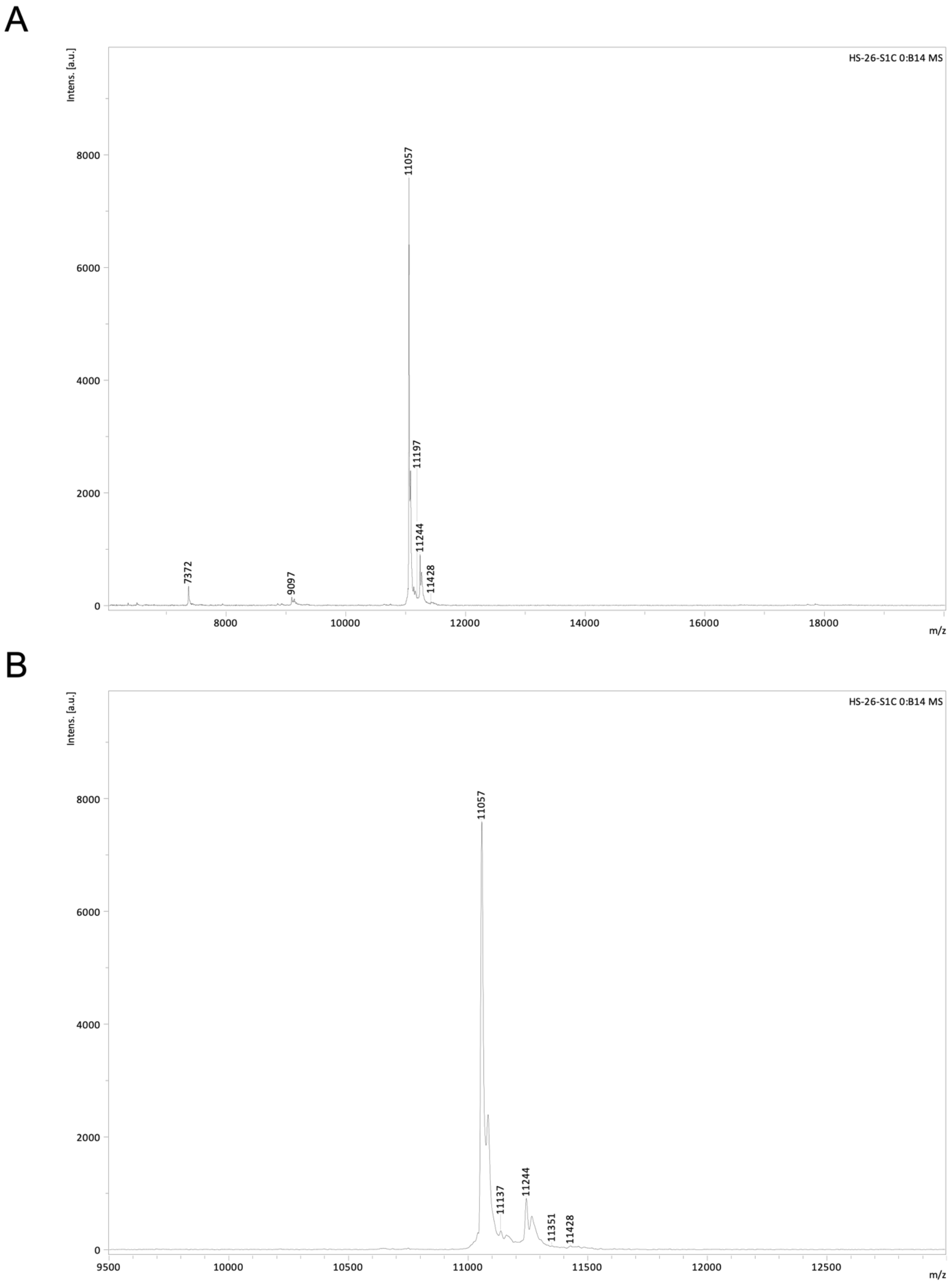

Figure S20. (A) MALDI-TOF-MS analysis of $CuCl_2$-treated Ftn-2x-**2**'s modified SUMO1 product, zoomed in, (B) with only two significant peaks identified at 11,057 Da and 11,244 Da, representing SUMO1 (MW = 11,058 Da) and SUMO1-1x-DEEM (MW = 11,244 Da; CVR = 9.1%), respectively.

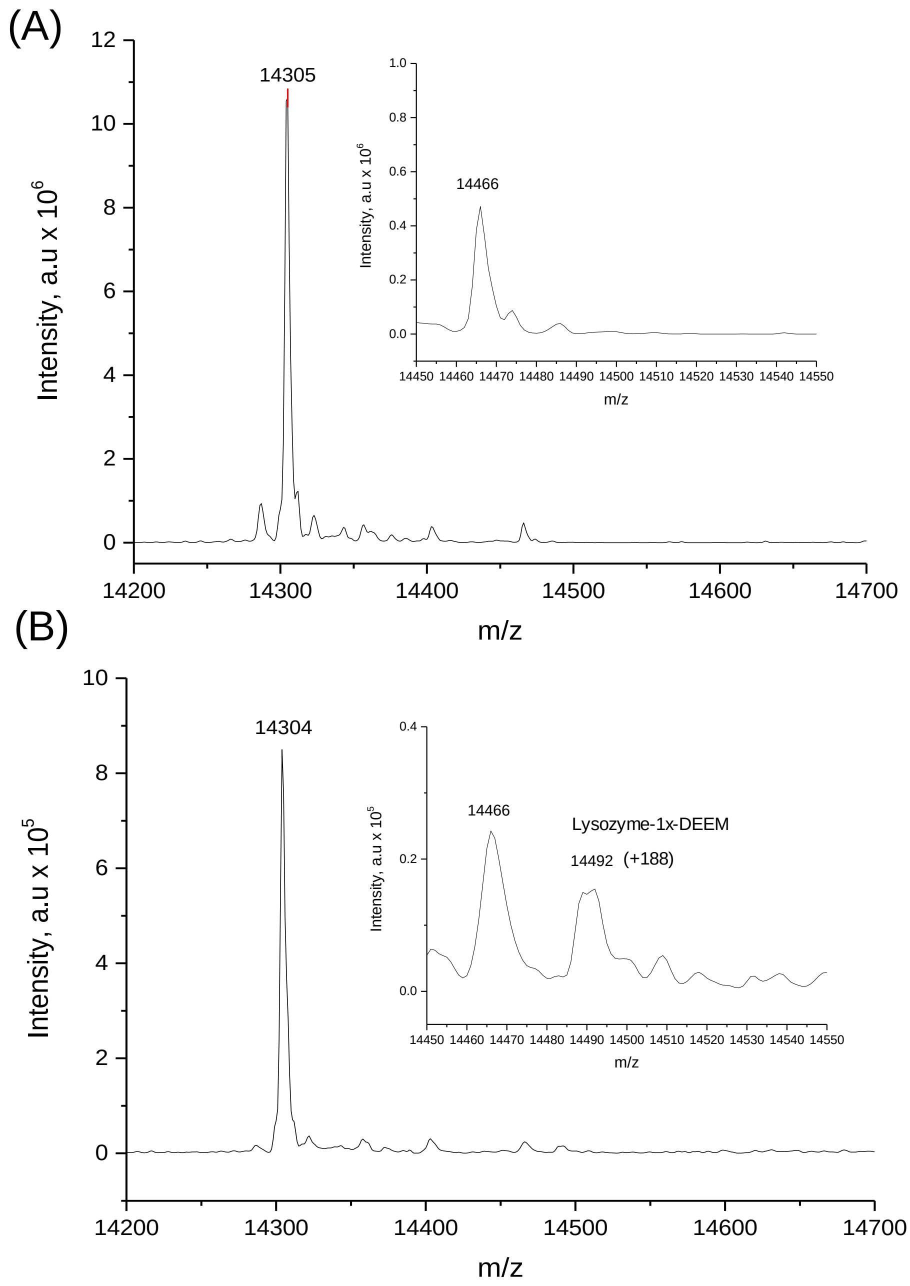

Figure S21. ESI-MS analyses of (A) lysozyme and (B) $CuCl_2$-treated Ftn-α-2x-**2**'s modified lysozyme product with spectrums zoomed in, in which a single large peak at 14,305 Da is observed in the former, representing the lysozyme protein alone, and an additional significant peak at 14,492 Da in the latter represents lysozyme-1x-DEEM (CVR = 1.6%).

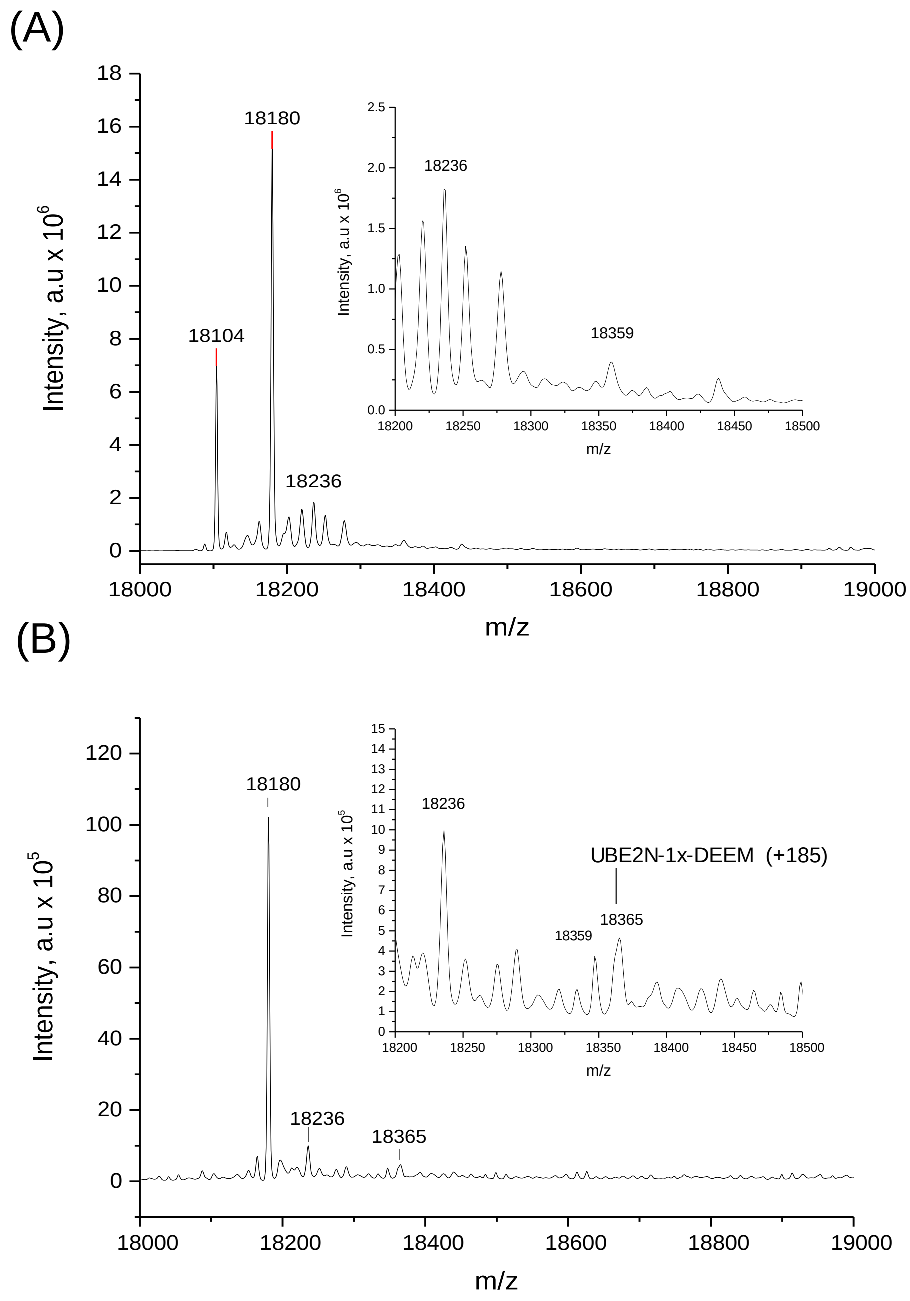

Figure S22. ESI-MS analyses of (A) UBE2N and (B) $CuCl_2$-treated Ftn-α-2x-**2**'s modified UBE2N product with spectrums zoomed in, in which a single large peak at 18,180 Da is observed in the former, representing the UBE2N protein alone, and the additional significant peak at 18,365 Da in the latter represents UBE2N-1x-DEEM (CVR = 4.0%).

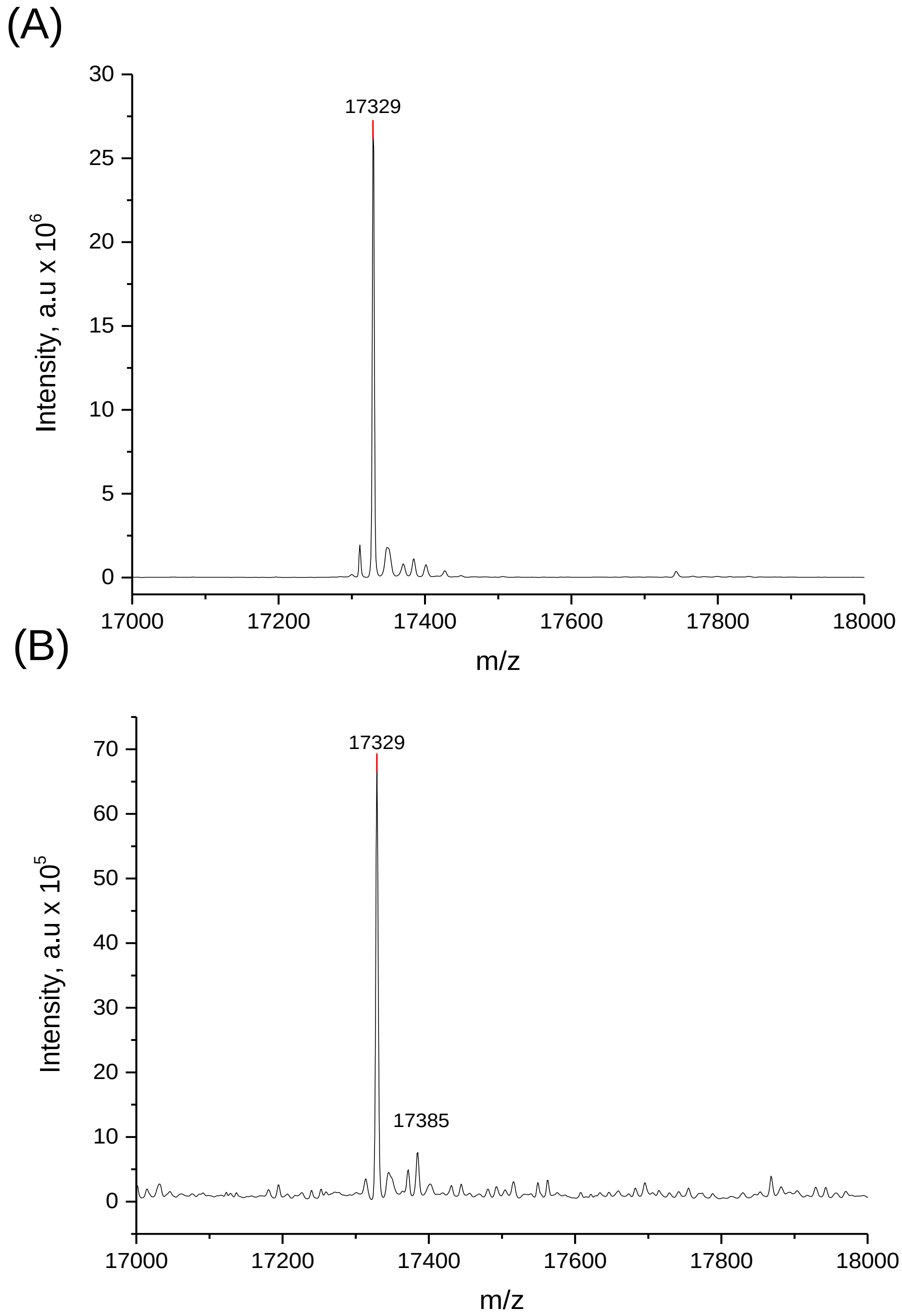

Figure S23. ESI-MS analyses of (A) UBE2V2 and (B) $CuCl_2$-treated Ftn-α-2x-**2**'s modified UBE2V2 product, in which a single large peak at 17,329 Da is observed in the former, representing the UBE2V2 protein alone, and the additional significant peak at 17,385 Da in the latter represents a modified UBE2V2 product with an unknown 56 Da adduct, respectively, with no DEEM-modified product.

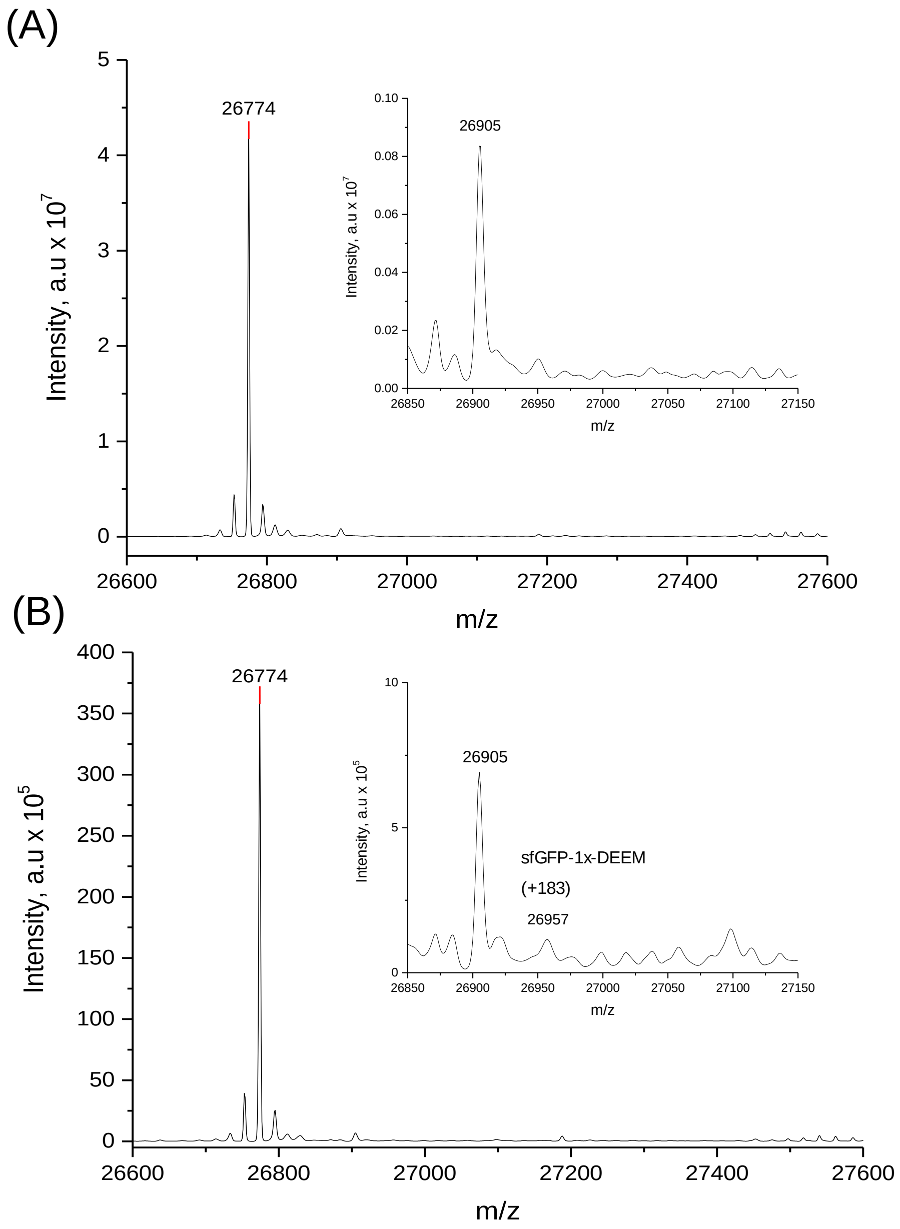

Figure S24. ESI-MS analyses of (A) sfGFP and (B) $CuCl_2$-treated Ftn-α-2x-**2**'s modified sfGFP product with spectrums zoomed in, in which a single large peak at 26,774 Da is observed in the former, representing the sfGFP protein alone, and an additional significant peak at 26,957 Da in the latter represents sfGFP-1x-DEEM (CVR = 0.30%).

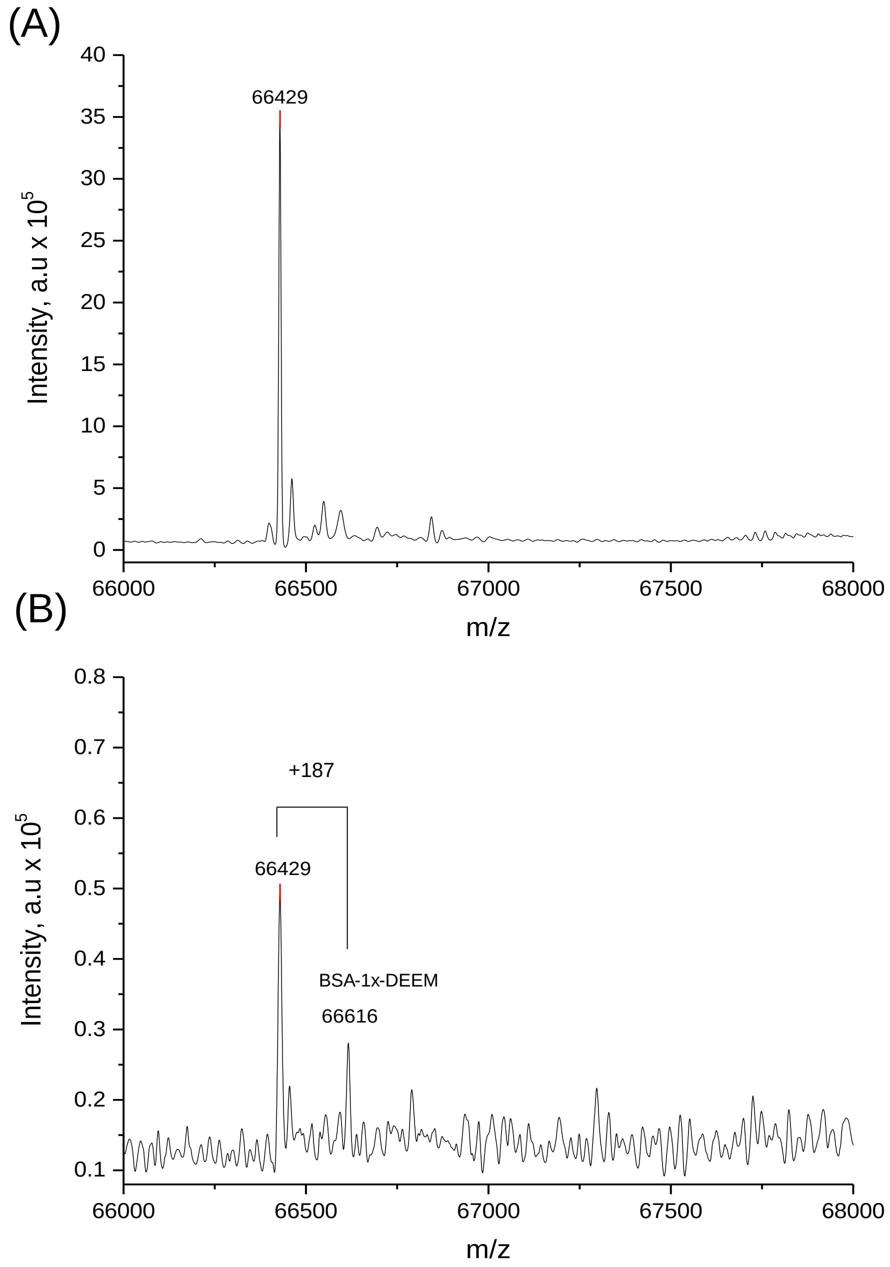

Figure S25. ESI-MS analyses of (A) BSA and (B) $CuCl_2$-treated Ftn-α-2x-**2**'s modified BSA product with spectrums zoomed in, in which a single large peak at 66,429 Da is observed in the former, representing the BSA protein alone, and an additional significant peak at 66,616 Da in the latter represents BSA-1x-DEEM (CVR = 36%).

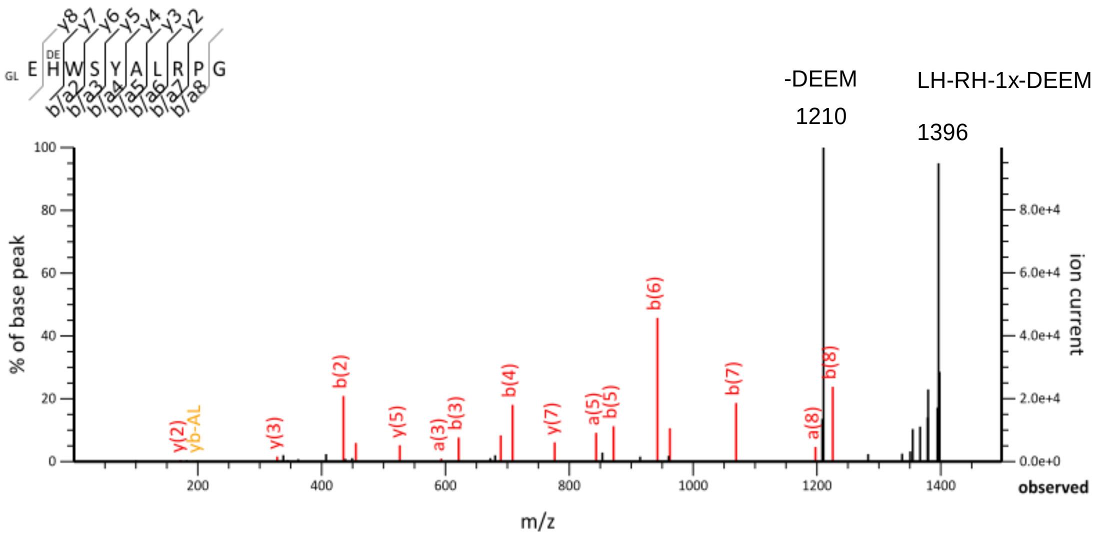

▼1 LHRH-1 59 LHRH-1

		Score	Mass	Matches	Sequences
1.1	LHRH-1	59	1228	1 (1)	1 (1) LHRH-1

▼*1 peptide matches (1 non-duplicate, 0 duplicate)*

Auto-fit to window

Query	Dupes	Observed	Mr(expt)	Mr(calc)	ppm	M	Score	Expect	Rank	U	Peptide
1		1396.7161	1395.7088	1395.6935	11.0	0	59	1.1e-06	▶1	U	-.EHWSYALRPG.- + DEEM (CH); Glu->pyro-Glu (N-term E)

Monoisotopic mass of neutral peptide Mr(calc): 1395.6935
Fixed modifications: Amidated (C-term),Methyl (L) (apply to specified residues or termini only)
Variable modifications:
N-term : Glu->pyro-Glu (N-term E)
H2 : DEEM (CH)
Ions Score: 59 **Expect:** 1.1e-06
Matches : 18/127 fragment ions using 22 most intense peaks (help)

#	Immon.	a	a*	a0	b	b*	b0	d	Seq.	v	w	y	y*	y0	#
1	102.0550	84.0444		66.0338	112.0393		94.0287	26.0389	E						10
2	296.1605	407.1925		389.1819	***435.1874***		417.1769		H	1017.5265		1285.6688	1268.6422	1267.6582	9
3	159.0917	**593.2718**		575.2613	***621.2667***		603.2562		W	831.4472		***962.5207***	945.4941	944.5101	8
4	60.0444	680.3039		662.2933	***708.2988***		690.2882	664.3089	S	744.4151	743.4199	***776.4413***	759.4148	758.4308	7
5	136.0757	**843.3672**		825.3566	***871.3621***		853.3515		Y	581.3518		***689.4093***	672.3828		6
6	44.0495	914.4043		896.3937	***942.3992***		924.3886		A	510.3147		***526.3460***	509.3194		5
7	100.1121	1041.5040		1023.4934	***1069.4989***		1051.4884	985.4414	L	383.2150	382.2197	***455.3089***	438.2823		4
8	129.1135	**1197.6051**	1180.5786	1179.5946	***1225.6000***	1208.5735	1207.5895	1112.5411	R	227.1139	226.1186	***328.2092***	311.1826		3
9	70.0651	1294.6579	1277.6313	1276.6473	1322.6528	1305.6262	1304.6422	1268.6422	P	130.0611	129.0659	***172.1081***			2
10	30.0338								G			75.0553			1

Seq	ya	yb	Seq	ya	yb	Seq	ya	yb
HW	482.2398	510.2347	HWS	569.2718	597.2667	WS	246.1237	274.1186
WSY	409.1870	437.1819	WSYA	480.2241	508.2191	WSYAL	607.3239	635.3188
SY	223.1077	251.1026	SYA	294.1448	322.1397	SYAL	421.2445	449.2395
SYALR	577.3457	605.3406	SYALRP	674.3984		YA	207.1128	235.1077
YAL	334.2125	362.2074	YALR	490.3136	518.3085	YALRP	587.3664	615.3613
AL	171.1492	199.1441	ALR	327.2503	355.2452	ALRP	424.3031	452.2980
LR	256.2132	284.2081	LRP	353.2660	381.2609	RP	226.1662	254.1612

Figure S26. MALDI-TOF-MS/MS analysis of $CuCl_2$-treated Ftn-2x-**2**'s modified [D-Ala^6, *N*-Me-Leu^7]-LH-RH product revealed DEEM modification to be carried out site-specifically on its H2. The two significant peaks at m/z = 1,210 and m/z = 1,396 represents DEEM and [D-Ala^6, *N*-Me-Leu^7]-LH-RH-1x-DEEM, respectively.

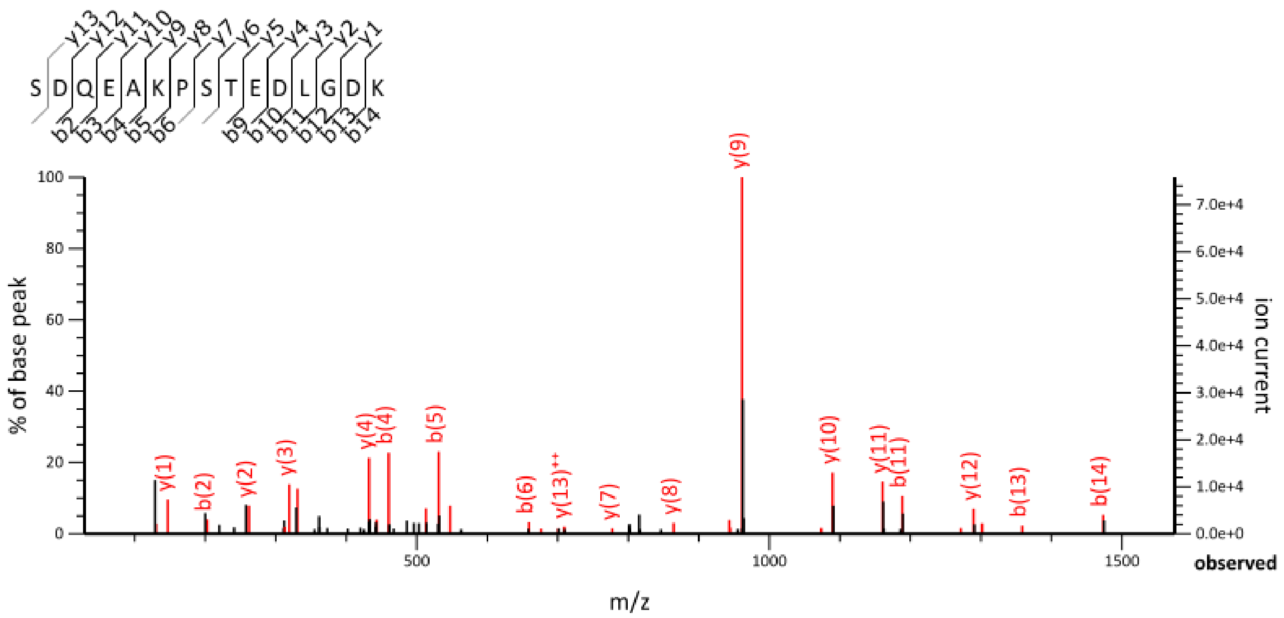

Monoisotopic mass of neutral peptide Mr(calc): 1618.7322
Ions Score: 84 **Expect:** 3.6e-09
Matches : 33/162 fragment ions using 57 most intense peaks (help)

#	b	b++	b*	b*++	b0	b0++	Seq.	y	y++	y*	y*++	y0	y0++	#
1	88.0393	44.5233			70.0287	35.5180	S							15
2	203.0662	102.0368			185.0557	93.0315	D	1532.7075	766.8574	1515.6809	758.3441	1514.6969	757.8521	14
3	331.1248	166.0661	314.0983	157.5528	313.1143	157.0608	Q	1417.6805	709.3439	1400.6540	700.8306	1399.6700	700.3386	13
4	460.1674	230.5873	443.1409	222.0741	442.1569	221.5821	E	1289.6220	645.3146	1272.5954	636.8013	1271.6114	636.3093	12
5	531.2045	266.1059	514.1780	257.5926	513.1940	257.1006	A	1160.5794	580.7933	1143.5528	572.2800	1142.5688	571.7880	11
6	659.2995	330.1534	642.2729	321.6401	641.2889	321.1481	K	1089.5422	545.2748	1072.5157	536.7615	1071.5317	536.2695	10
7	756.3523	378.6798	739.3257	370.1665	738.3417	369.6745	P	961.4473	481.2273	944.4207	472.7140	943.4367	472.2220	9
8	843.3843	422.1958	826.3577	413.6825	825.3737	413.1905	S	864.3945	432.7009	847.3680	424.1876	846.3840	423.6956	8
9	944.4320	472.7196	927.4054	464.2063	926.4214	463.7143	T	777.3625	389.1849	760.3359	380.6716	759.3519	380.1796	7
10	1073.4746	537.2409	1056.4480	528.7276	1055.4640	528.2356	E	676.3148	338.6610	659.2883	330.1478	658.3042	329.6558	6
11	1188.5015	594.7544	1171.4750	586.2411	1170.4909	585.7491	D	547.2722	274.1397	530.2457	265.6265	529.2617	265.1345	5
12	1301.5856	651.2964	1284.5590	642.7831	1283.5750	642.2911	L	432.2453	216.6263	415.2187	208.1130	414.2347	207.6210	4
13	1358.6070	679.8072	1341.5805	671.2939	1340.5965	670.8019	G	319.1612	160.0842	302.1347	151.5710	301.1506	151.0790	3
14	1473.6340	737.3206	1456.6074	728.8073	1455.6234	728.3153	D	262.1397	131.5735	245.1132	123.0602	244.1292	122.5682	2
15							K	147.1128	74.0600	130.0863	65.5468			1

Figure S27. ESI-MS/MS analysis of the N-terminal peptide (S^{1}DQEAKPSTEDLGDK^{15}) of $CuCl_2$-treated Ftn-2x-**2**'s modified SUMO1 product with DEEM modification.

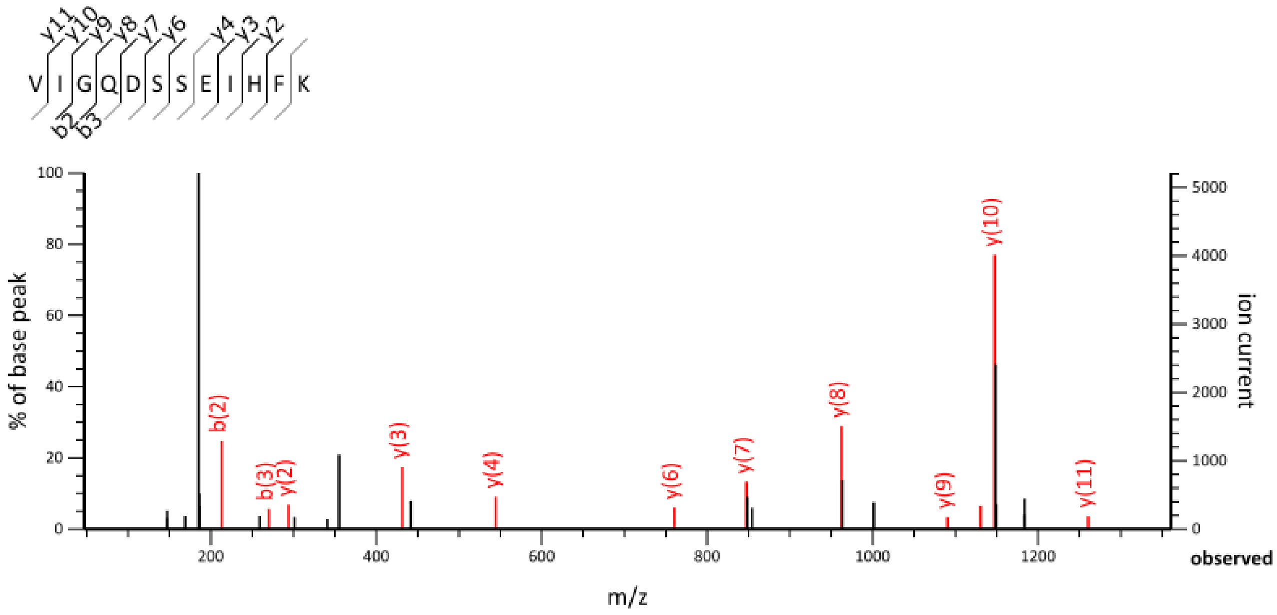

Monoisotopic mass of neutral peptide Mr(calc): 1358.6830
Ions Score: 77 **Expect:** 2e-08
Matches : 12/110 fragment ions using 17 most intense peaks (help)

#	b	b^{++}	b*	b^{*++}	b^{0}	b^{0++}	Seq.	y	y^{++}	y*	y^{*++}	y^{0}	y^{0++}	#
1	100.0757	50.5415					V							12
2	**213.1598**	107.0835					I	***1260.6219***	630.8146	1243.5953	622.3013	1242.6113	621.8093	11
3	**270.1812**	135.5942					G	***1147.5378***	574.2726	1130.5113	565.7593	1129.5273	565.2673	10
4	398.2398	199.6235	381.2132	191.1103			Q	***1090.5164***	545.7618	1073.4898	537.2485	1072.5058	536.7565	9
5	513.2667	257.1370	496.2402	248.6237	495.2562	248.1317	D	***962.4578***	481.7325	945.4312	473.2193	944.4472	472.7272	8
6	600.2988	300.6530	583.2722	292.1397	582.2882	291.6477	S	***847.4308***	424.2191	830.4043	415.7058	829.4203	415.2138	7
7	687.3308	344.1690	670.3042	335.6558	669.3202	335.1638	S	***760.3988***	380.7030	743.3723	372.1898	742.3883	371.6978	6
8	816.3734	408.6903	799.3468	400.1771	798.3628	399.6850	E	673.3668	337.1870	656.3402	328.6738	655.3562	328.1817	5
9	929.4575	465.2324	912.4309	456.7191	911.4469	456.2271	I	***544.3242***	272.6657	527.2976	264.1525			4
10	1066.5164	533.7618	1049.4898	525.2485	1048.5058	524.7565	H	***431.2401***	216.1237	414.2136	207.6104			3
11	1213.5848	607.2960	1196.5582	598.7828	1195.5742	598.2907	F	***294.1812***	147.5942	277.1547	139.0810			2
12							K	147.1128	74.0600	130.0863	65.5468			1

Figure S28. ESI-MS/MS analysis of the H34-containing peptide ($V^{25}IGQDSSEIH^{34}FK^{36}$) of $CuCl_2$-treated Ftn-2x-**2**'s modified SUMO1 product with DEEM modification.

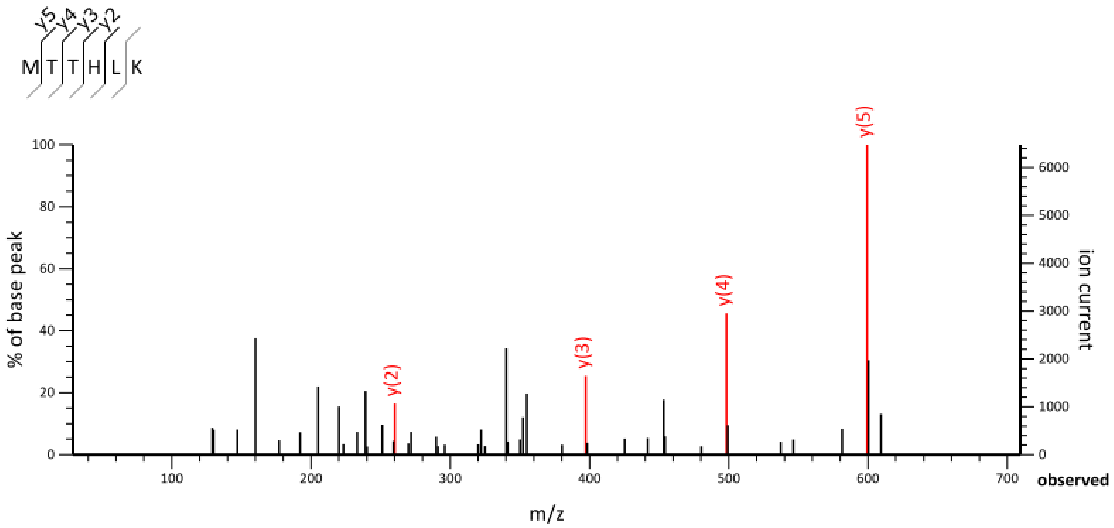

Monoisotopic mass of neutral peptide Mr(calc): 729.3843
Ions Score: 21 **Expect:** 0.0071
Matches : 4/42 fragment ions using 10 most intense peaks (help)

#	b	b^{++}	b^{0}	b^{0++}	Seq.	y	y^{++}	y*	y^{*++}	y^{0}	y^{0++}	#
1	132.0478	66.5275			M							6
2	233.0954	117.0514	215.0849	108.0461	T	***599.3511***	300.1792	582.3246	291.6659	581.3406	291.1739	5
3	334.1431	167.5752	316.1326	158.5699	T	***498.3035***	249.6554	481.2769	241.1421	480.2929	240.6501	4
4	471.2020	236.1047	453.1915	227.0994	H	***397.2558***	199.1315	380.2292	190.6183			3
5	584.2861	292.6467	566.2755	283.6414	L	***260.1969***	130.6021	243.1703	122.0888			2
6					K	147.1128	74.0600	130.0863	65.5468			1

Figure S29. ESI-MS/MS analysis of the H42-containing peptide ($M^{39}TTH^{42}LK^{44}$) of $CuCl_2$-treated Ftn-2x-**2**'s modified SUMO1 product with DEEM modification.

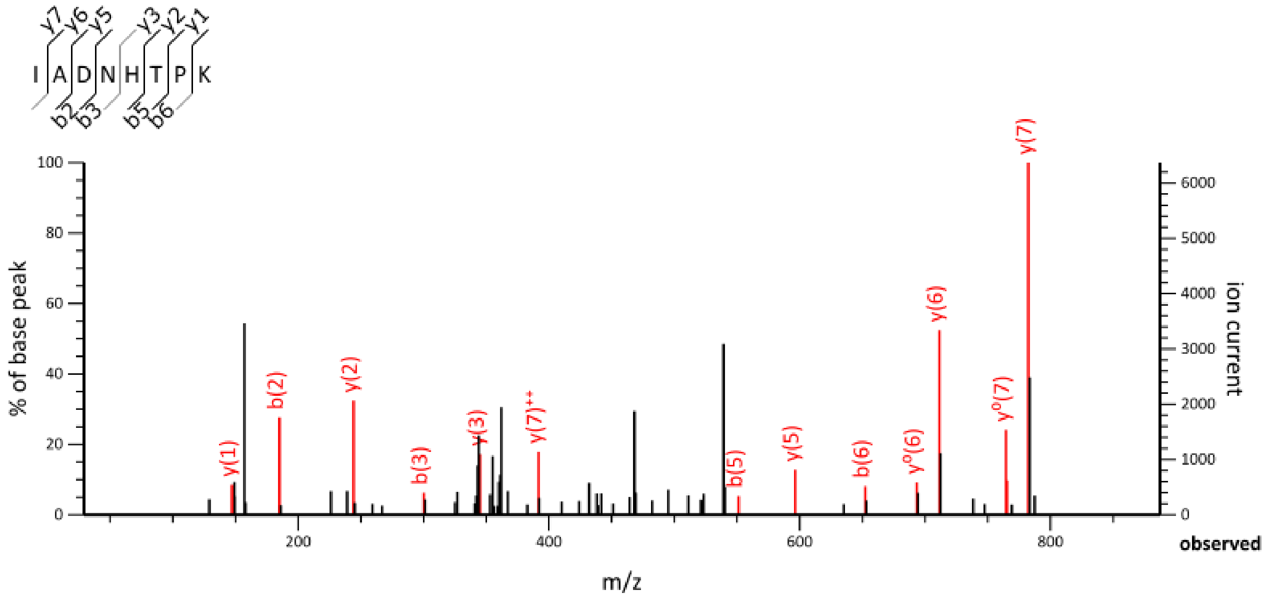

Monoisotopic mass of neutral peptide Mr(calc): 894.4559
Ions Score: 35 **Expect:** 0.00032
Matches : 14/70 fragment ions using 28 most intense peaks (help)

#	b	b++	b*	b*++	b0	b0++	Seq.	y	y++	y*	y*++	y0	y0++	#
1	114.0913	57.5493					I							8
2	**185.1285**	93.0679					A	***782.3791***	**391.6932**	765.3526	383.1799	764.3686	382.6879	7
3	**300.1554**	150.5813			282.1448	141.5761	D	***711.3420***	356.1747	694.3155	347.6614	693.3315	347.1694	6
4	414.1983	207.6028	397.1718	199.0895	396.1878	198.5975	N	***596.3151***	298.6612	579.2885	290.1479	578.3045	289.6559	5
5	**551.2572**	276.1323	534.2307	267.6190	533.2467	267.1270	H	482.2722	241.6397	465.2456	233.1264	464.2616	232.6344	4
6	**652.3049**	326.6561	635.2784	318.1428	634.2944	317.6508	T	***345.2132***	173.1103	328.1867	164.5970	327.2027	164.1050	3
7	749.3577	375.1825	732.3311	366.6692	731.3471	366.1772	P	***244.1656***	122.5864	227.1390	114.0731			2
8							K	***147.1128***	74.0600	130.0863	65.5468			1

Figure S30. ESI-MS/MS analysis of the H74-containing peptide (I[70]ADNH[74]TPK[77]) of $CuCl_2$-treated Ftn-2x-**2**'s modified SUMO1 product with DEEM modification.

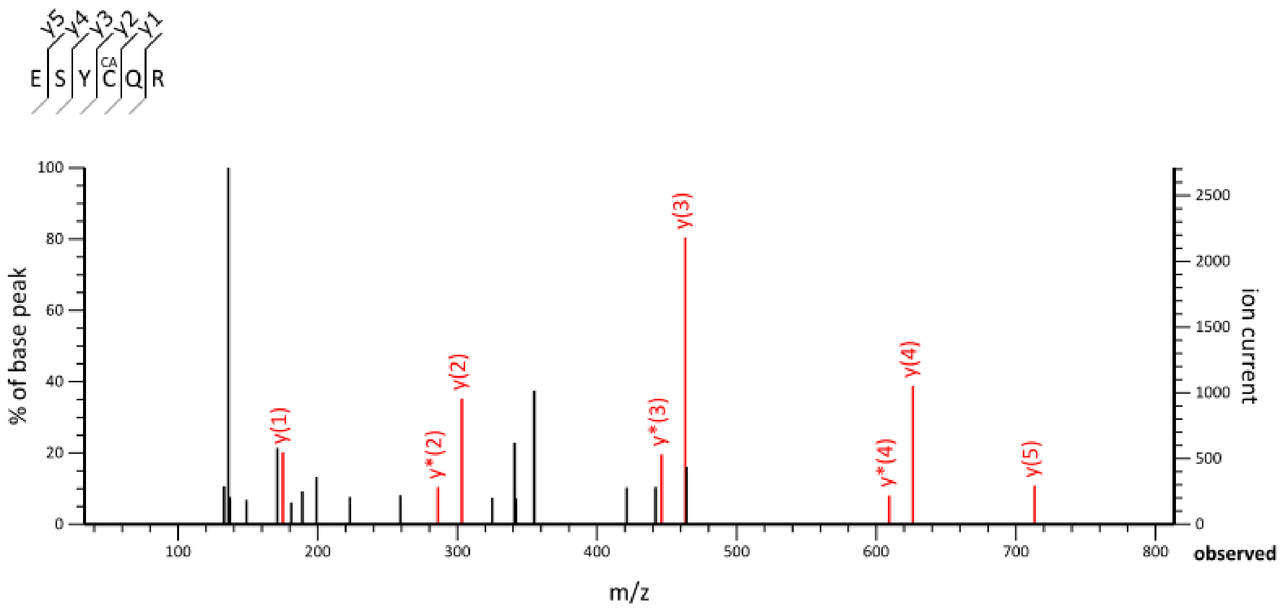

Monoisotopic mass of neutral peptide Mr(calc): 841.3388
Variable modifications:
C4 : Carbamidomethyl (C)
Ions Score: 33 **Expect:** 0.00054
Matches : 8/44 fragment ions using 15 most intense peaks (help)

#	b	b++	b*	b*++	b0	b0++	Seq.	y	y++	y*	y*++	y0	y0++	#
1	130.0499	65.5286			112.0393	56.5233	E							6
2	217.0819	109.0446			199.0713	100.0393	S	***713.3035***	357.1554	696.2770	348.6421	695.2930	348.1501	5
3	380.1452	190.5763			362.1347	181.5710	Y	***626.2715***	313.6394	609.2450	305.1261			4
4	540.1759	270.5916			522.1653	261.5863	C	***463.2082***	232.1077	446.1816	223.5945			3
5	668.2345	334.6209	651.2079	326.1076	650.2239	325.6156	Q	***303.1775***	152.0924	286.1510	143.5791			2
6							R	***175.1190***	88.0631	158.0924	79.5498			1

Figure S31. ESI-MS/MS analysis of the C51-containing peptide (E[48]SYC[51]QR[53]) of $CuCl_2$-treated Ftn-2x-**2**'s modified SUMO1 product with DEEM modification.

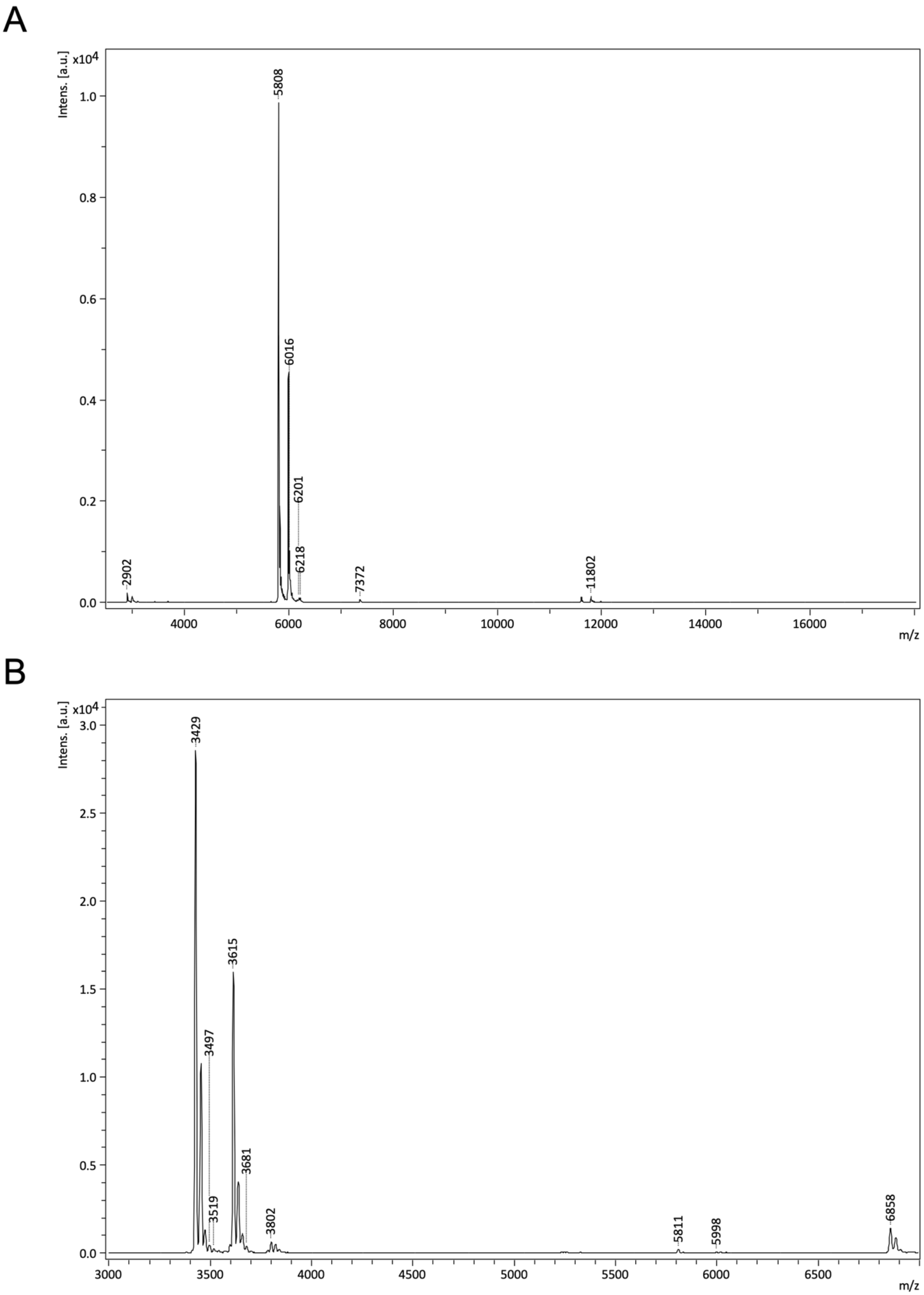

Figure S32. MALDI-TOF-MS analyses of the DTT-digested, 20% (v/v) acetonitrile and 0.1% (v/v) formic acid-dissolved (A) insulin B-chain (MW = 3,430 Da) and (B) modified insulin product's B-chain of $CuCl_2$-treated Ftn-2x-**2**, revealing three notable peaks in the latter at 3,429 Da, 3,615 Da and 3,802 Da, which represents insulin B chain, insulin B chain-1x-DEEM (MW = 3,616 Da) and insulin B chain-2x-DEEM (MW = 3,802 Da), respectively, with the last being only in trace amounts.

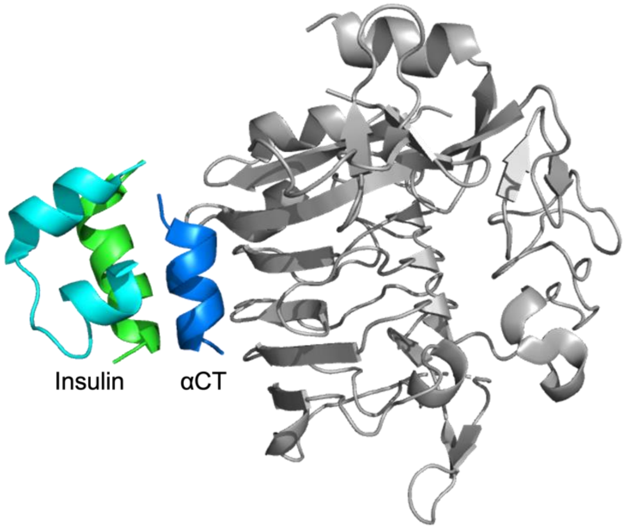

Figure S33. The αCT peptide (blue) isolated from the insulin receptor's (PDB code: 3W11) water-soluble ectodomain is known to build contacts with both insulin A- and B-chains (green and cyan, respectively), and therefore may serve as an insulin target recognition peptide (TRP).

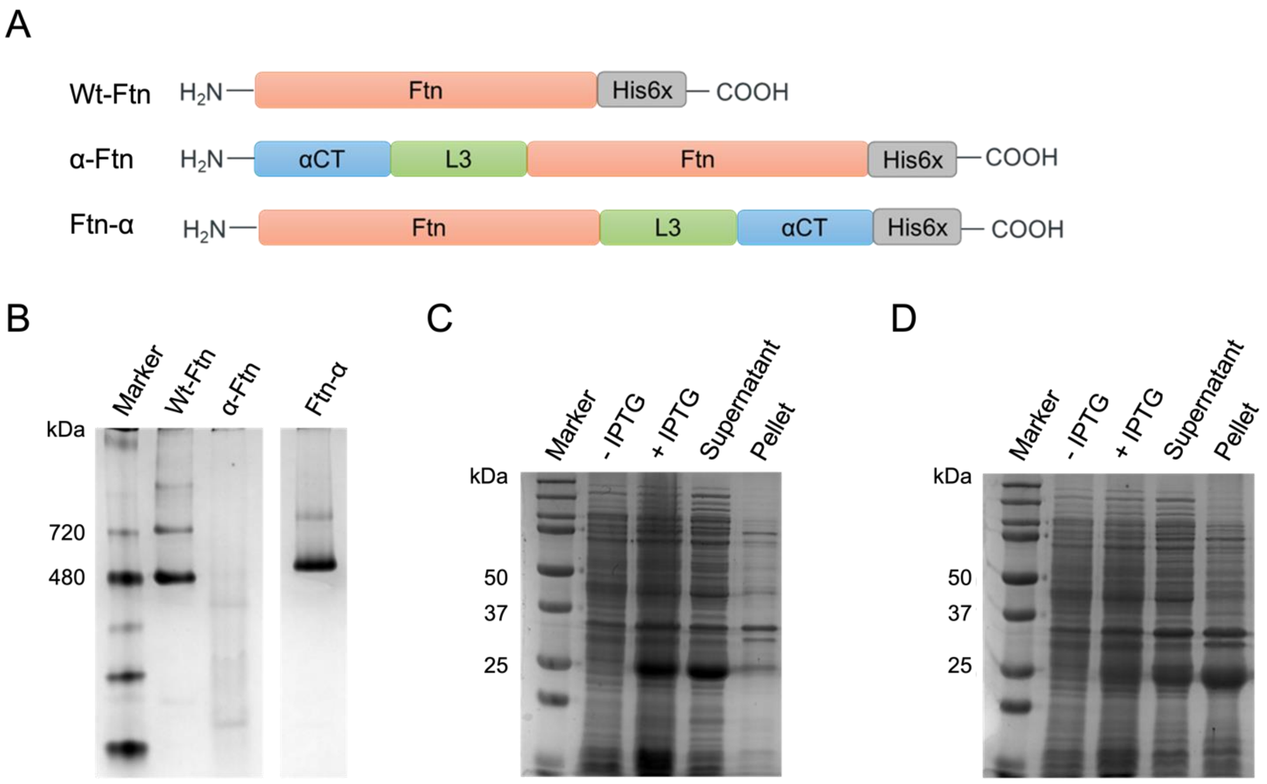

Figure S34. (A) Wild-type ferritin (wt-Ftn) (MW = 531 kDa), α-Ftn (MW = 624 kDa) and Ftn-α (MW = 624 kDa) were subjected to (B) 7.5% native PAGE to examine their self-assembling capacities; *E. coli* cell lysates expressing (C) α-Ftn and (D) Ftn-α were further subjected to individual 12% SDS-PAGE solubility tests, respectively.

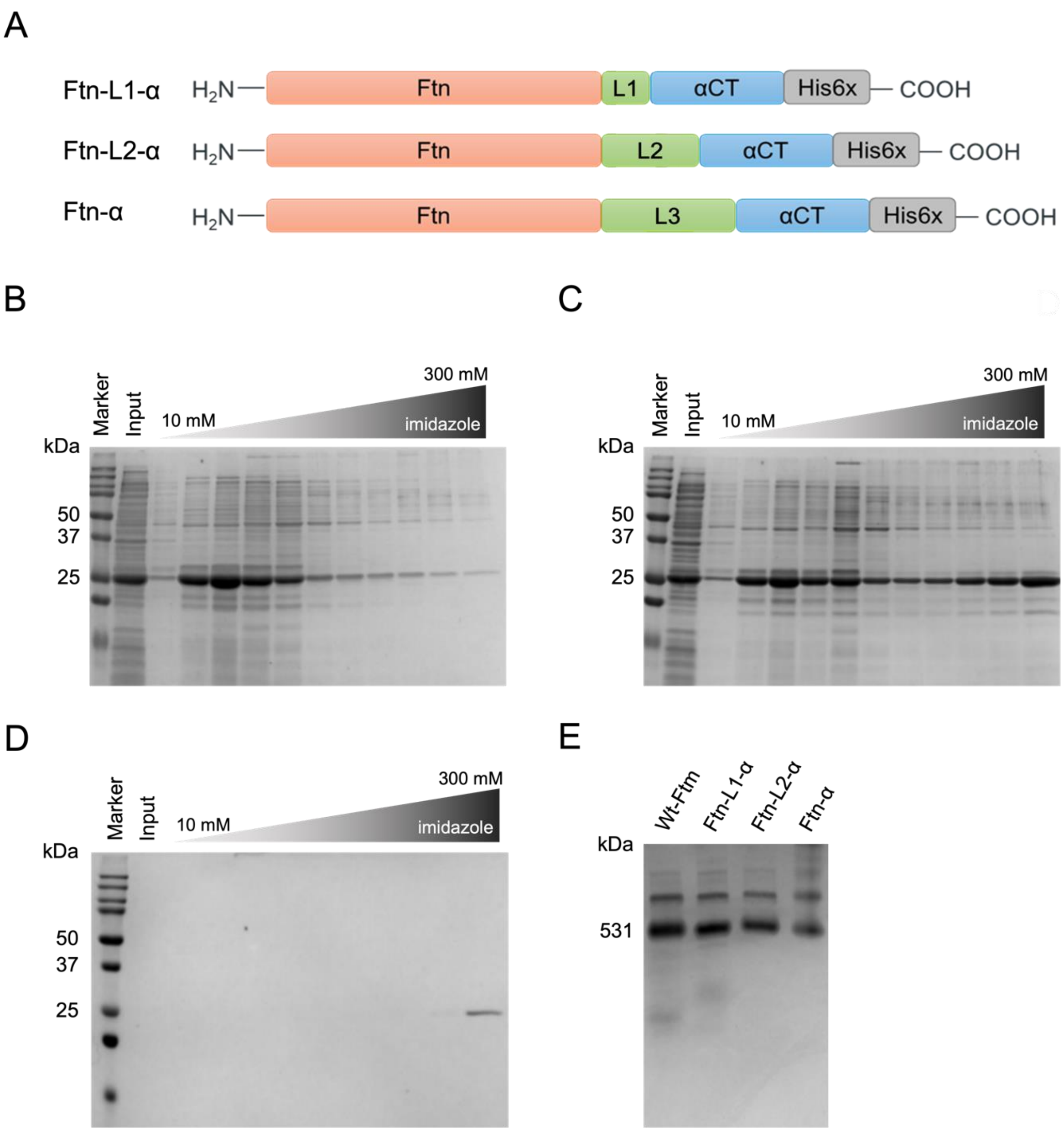

Figure S35. Gradient elution profiles (10-300 mM) of (A) the C-terminally fused-αCT Ftn variants with linkers of varying lengths: Ftn-L1-α (MW = 609 kDa), Ftn-L2-α (MW = 616 kDa) and Ftn-α (MW = 624 kDa) were subjected to (B-D) 12% SDS-PAGE to examine the 6xHis-tags' accessibility for optimal purification, and (E) 7.5% native PAGE to examine each variant's self-assembling capacity.

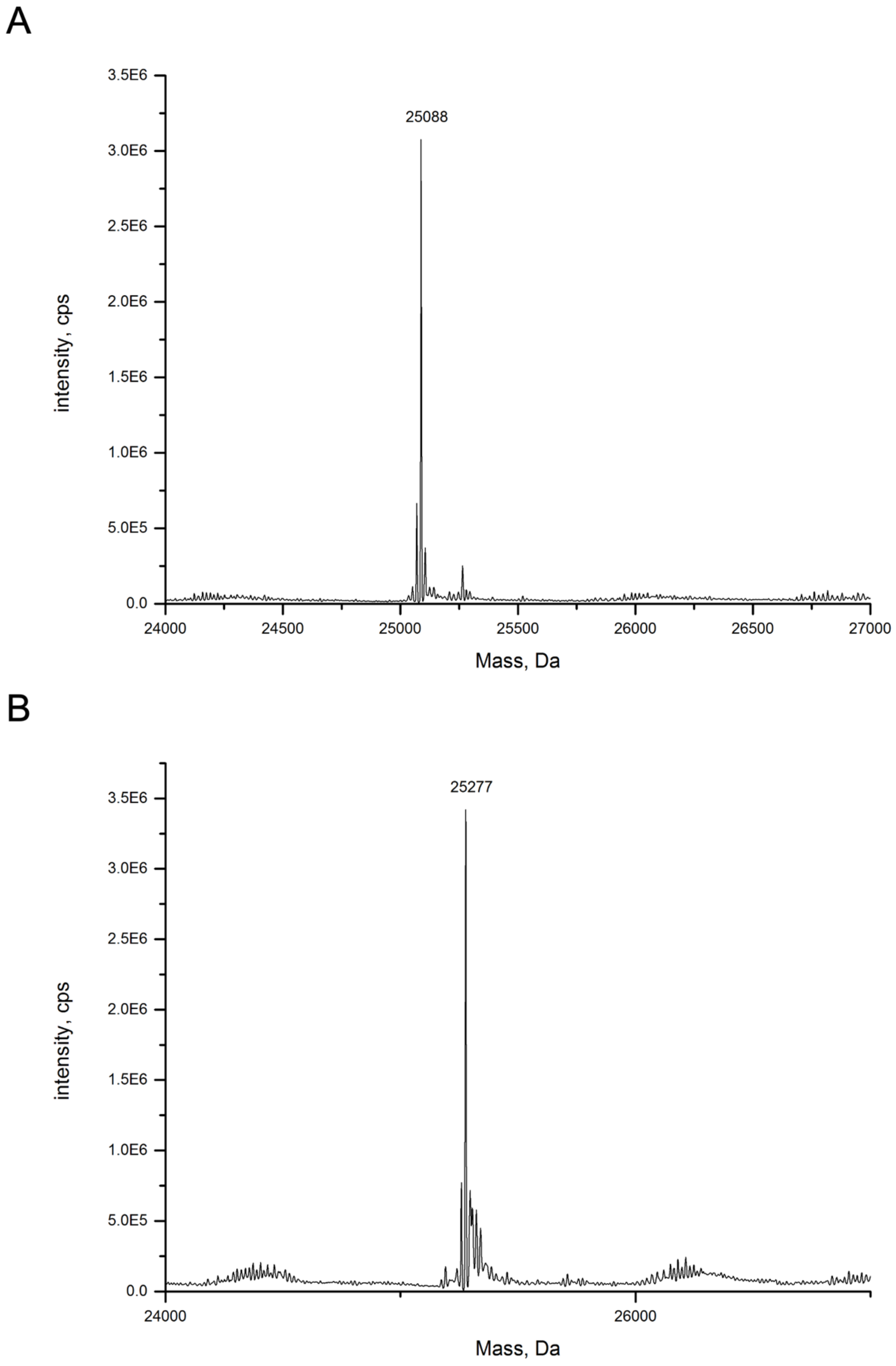

Figure S36. ESI-MS analyses of (A) F-α-1x-**3** (MW = 25,089 kDa) and (B) F-α-2x-**2** (MW = 25,274 kDa) verifying homogenous histidine analog incorporation to be carried out for both variants via the *Methanosarcina mazei* (*Mm*) PylRS-N346A/C348A•tRNA^{Pyl} pairs in *E. coli* BL21(DE3).

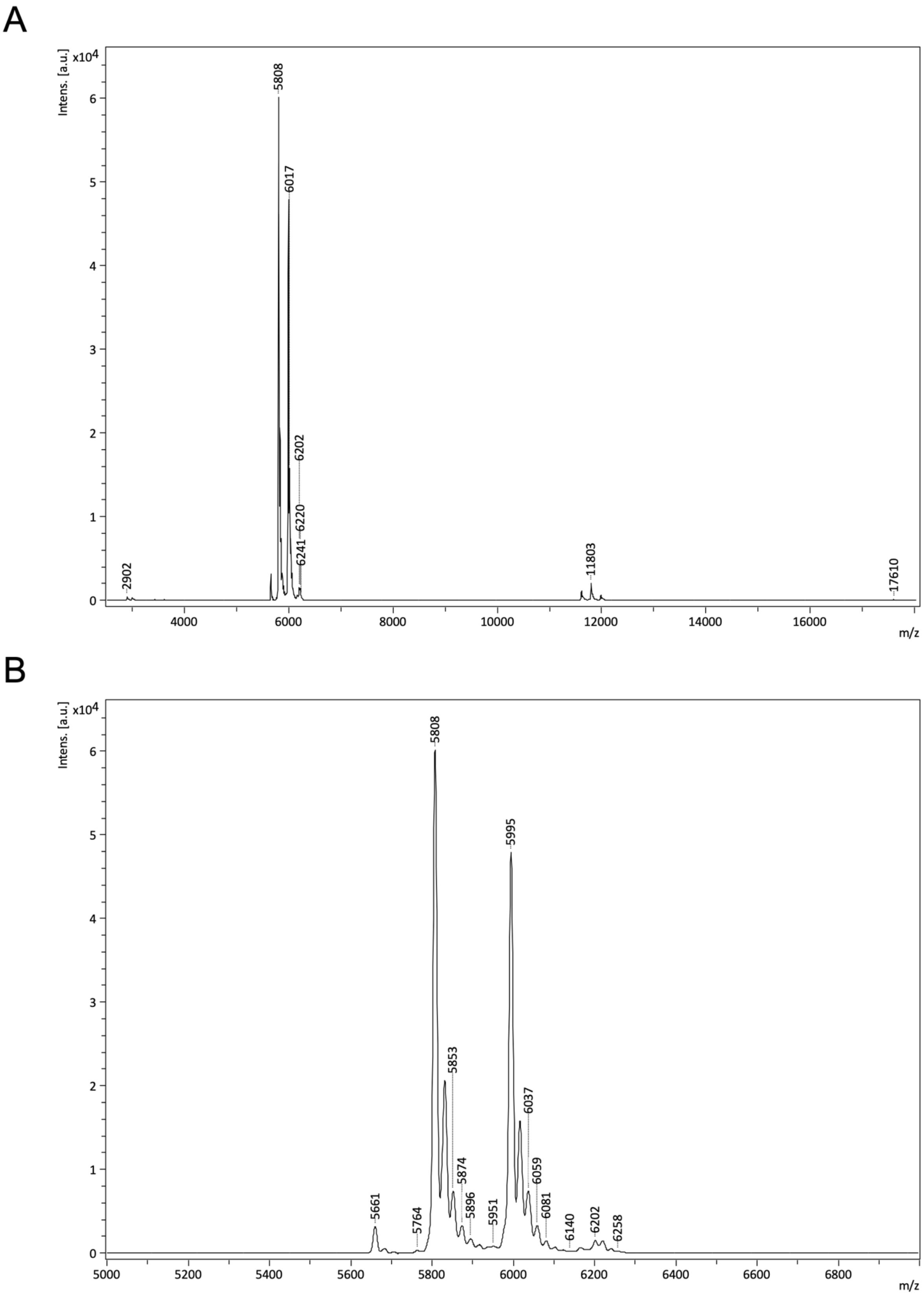

Figure S37. (A) MALDI-TOF-MS analysis of $CuCl_2$-treated Ftn-α-1x-**3**'s modified insulin product, (B) zoomed in, with only two significant peaks identified, representing insulin (MW = 5,808 Da) and insulin-1x-DEEM (MW = 5,994 Da; CVR = 46%, turnover number (TON) = 9.2 $hr^{-1}ball^{-1}$), respectively.

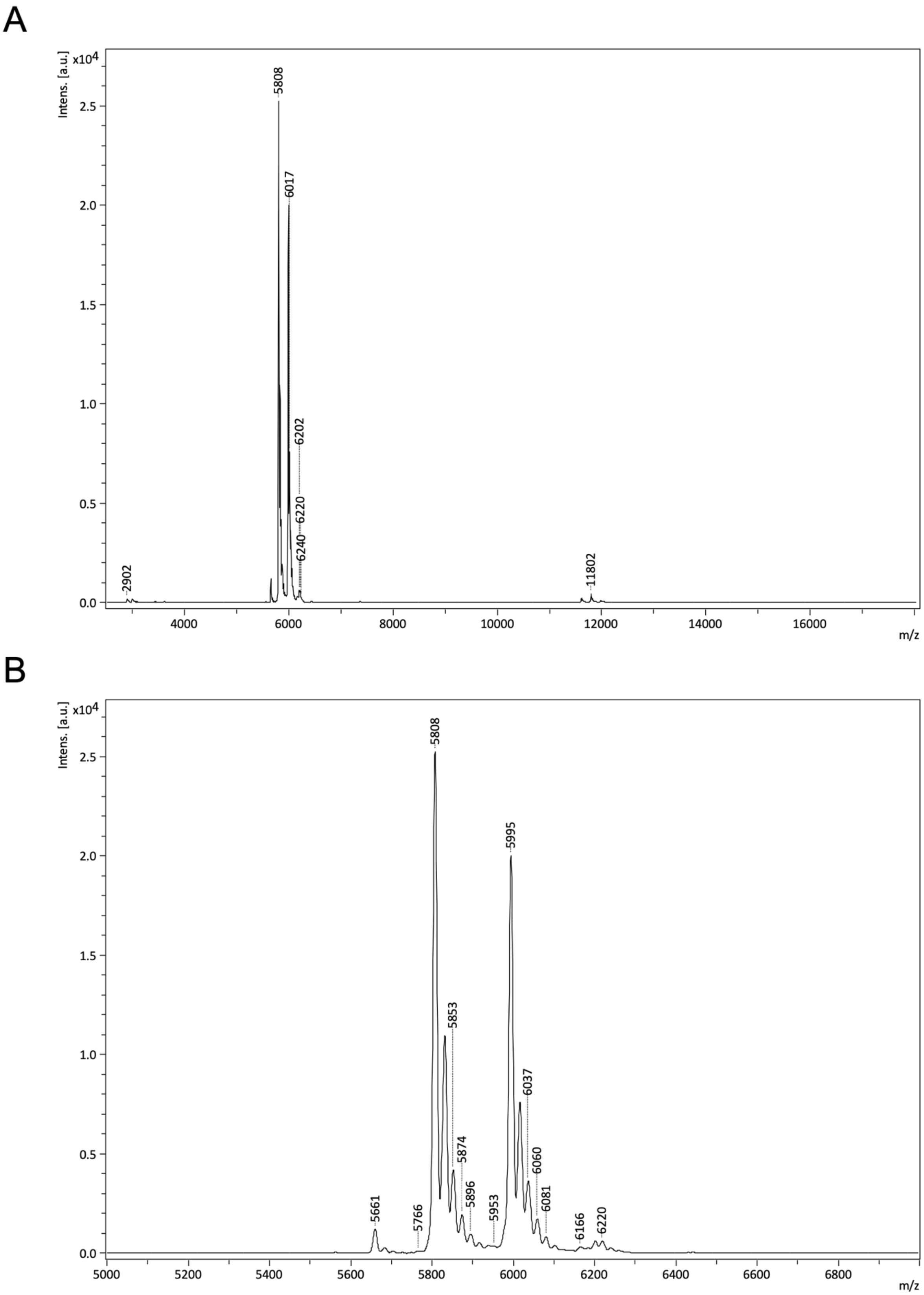

Figure S38. (A) MALDI-TOF-MS analysis of $CuCl_2$-treated Ftn-α-2x-**2**'s modified insulin product, (B) zoomed in, also with only two significant peaks identified, representing insulin (MW = 5,808 Da) and insulin-1x-DEEM (MW = 5,995 Da), respectively, with near quantitative conversion (CVR = ~100%, TON = 24 $hr^{-1}ball^{-1}$).

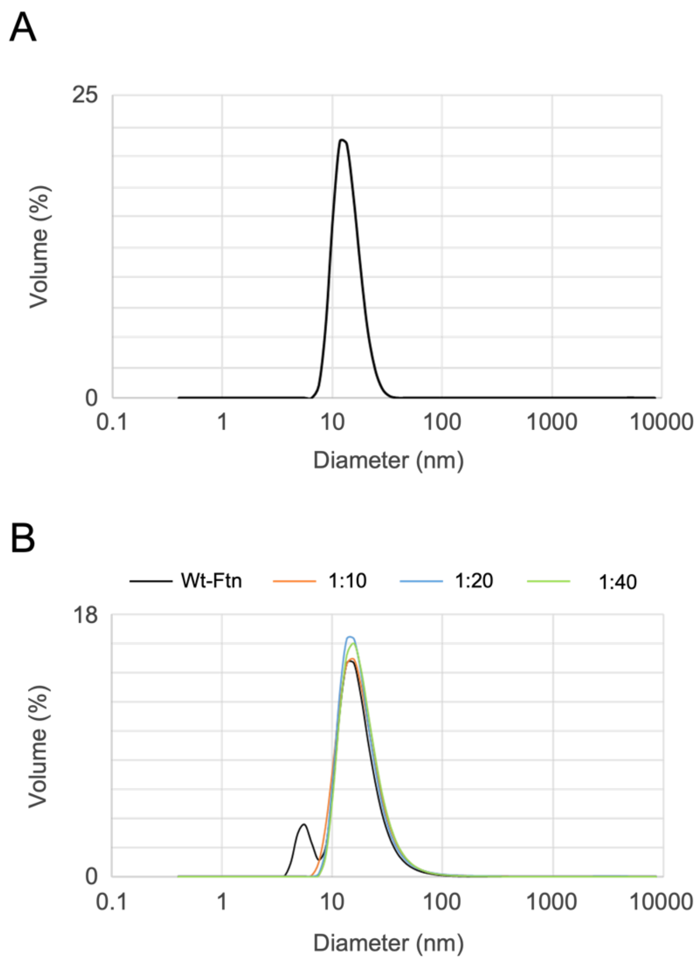

Figure S39. DLS analysis of (A) wt-Ftn as the control, and (B) mixtures of [F-α-2x-**2**]:[insulin] = 1:10, 1:20 and 1:40, revealing the variant to share similar morphology in terms of size and shape with wt-Ftn.

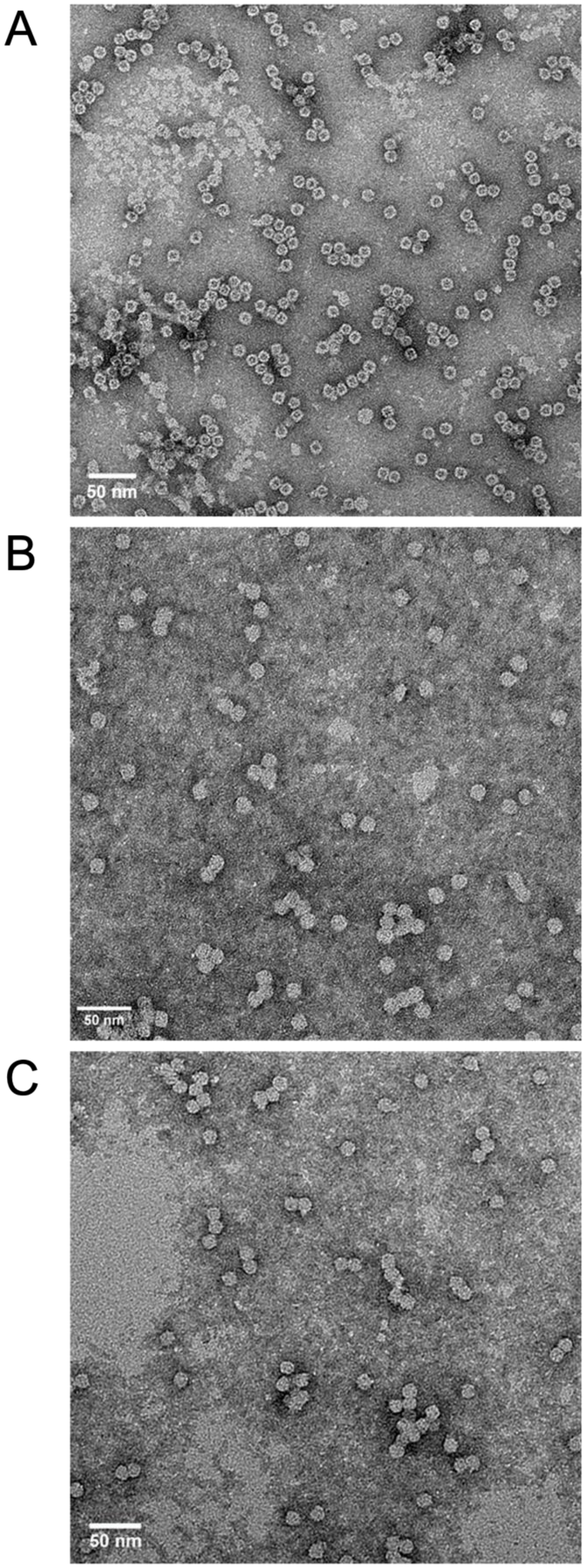

Figure S40. FEG-TEM images of (A) wt-Ftn, (B) F-α-2x-**2** and (C) mixture of [F-α-2x-**2**]:[insulin] = 1:20, revealing that the F-α-2x-**2** variant remains assembled when mixed with its insulin substrate.
